# CKMT1 Counteracts VDAC1 Oligomerization‐Induced Neuronal Pyroptosis Through Steric Hindrance in Ischemic Stroke

**DOI:** 10.1002/advs.78611

**Published:** 2026-11-05

**Authors:** Xiaoyu Wang, Sai Shi, Jianghui Wu, Jingge Zhang, Shuqin Li, Yuyan Hu, Haifeng Yin, Chenguang Zhao, Dong Ma, Xiaohui Xian, Lizhe Liu, Ling Zhao, Xiyun Liu, Zhikai Yu, Lihua Dong, Wenbin Li, Lingyan Zhang, Min Zhang

**Affiliations:** ^1^ Department of Pathophysiology Hebei Medical University Shijiazhuang People's Republic of China; ^2^ Department of Medical and Pharmaceutical Informatics Hebei Medical University Shijiazhuang People's Republic of China; ^3^ Hebei Key Laboratory of Critical Disease Mechanism and Intervention Shijiazhuang People's Republic of China; ^4^ The Key Laboratory of Neural and Vascular Biology Hebei Medical University Shijiazhuang People's Republic of China; ^5^ Foreign Languages Department Hebei Medical University Shijiazhuang People's Republic of China; ^6^ Department of Biochemistry and Molecular Biology Hebei Medical University Shijiazhuang People's Republic of China; ^7^ Department of Obstetrics The Fourth Hospital of Hebei Medical University Shijiazhuang People's Republic of China; ^8^ Transformation Center for Scientific and Technological Achievements Hebei University of Chinese Medicine Shijiazhuang People's Republic of China; ^9^ Undergraduate Student in Pediatrics Hebei Medical University Shijiazhuang People's Republic of China; ^10^ Hebei Key Laboratory of Forensic Medicine Hebei Medical University Shijiazhuang People's Republic of China

**Keywords:** absent in melanoma 2 (AIM2), creatine kinase mitochondrial 1 (CKMT1), ischemic stroke, mitochondrial DNA (mtDNA), pyroptosis, voltage‐dependent anion channel 1 (VDAC1) oligomerization

## Abstract

Ischemic stroke is one of the leading causes of death and disability worldwide; yet effective therapeutic options remain limited. Cytosolic leakage of mitochondrial DNA (mtDNA) is a critical driver of ischemic brain injury. Voltage‐dependent anion channel 1 (VDAC1), embedded in the mitochondrial outer membrane, promotes mtDNA leakage through oligomerization. However, the regulatory mechanisms underlying VDAC1 oligomerization in ischemic stroke remain poorly understood. This study demonstrates that ischemic stroke promotes VDAC1 oligomerization, leading to mtDNA leakage, activating the absent in melanoma 2 (AIM2) inflammasome, and subsequent neuronal pyroptosis. Pharmacological inhibition of VDAC1 oligomerization using VBIT‐4 effectively disrupts this cascade. Creatine kinase mitochondrial 1 (CKMT1) is identified as a novel endogenous inhibitor of VDAC1 oligomerization. CKMT1 inhibits VDAC1 oligomerization through a dual‐region synergistic mechanism. Specifically, the N‐terminal domain of CKMT1 (residues R75 and E121) forms a stable interface with the C‐terminal domain of VDAC1 (residues R218 and E189), whereas the CKMT1 signal peptide (residues 1–39) sterically hinders VDAC1 dimerization, thereby blocking oligomerization. Moreover, neuronal overexpression of CKMT1 significantly attenuates neuronal apoptosis, whereas CKMT1 knockdown aggravates neuronal injury. Collectively, these findings demonstrate that CKMT1 protects against cerebral ischemic injury by suppressing the VDAC1–mtDNA–AIM2 axis and identify CKMT1 as a novel therapeutic target for ischemic stroke.

## Graphical abstract

CKMT1 inhibits VDAC1 oligomerization via a dual‐region mechanism: its NTD interacts with the VDAC1 C‐terminal domain, while residues 1–39 sterically hinder dimerization, thereby reducing mtDNA leakage and AIM2 inflammasome activation, suppressing neuronal pyroptosis and protecting against cerebral ischemic injury.

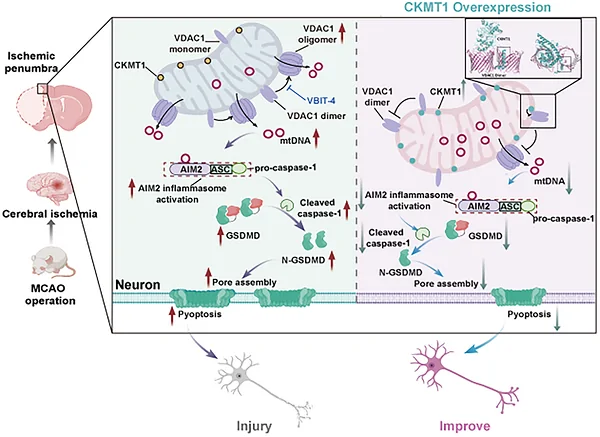

## 1 Introduction

As the global population ages, stroke has become the second leading cause of death worldwide. Ischemic strokes, caused by cerebral artery occlusion, account for more than 70% of all stroke cases [1]. Although reperfusion is essential for salvaging the ischemic penumbra and restoring neurological function, it can paradoxically induce cerebral ischemia‐reperfusion injury (CIRI). The underlying mechanisms of CIRI include excitotoxicity, energy metabolism dysfunction, oxidative stress, and inflammatory immune responses [2, 3, 4, 5]. Despite advances in therapeutic strategies, the molecular mechanisms regulating CIRI remain incompletely understood, and effective clinical interventions are still lacking.

Mitochondria regulate neuronal energy metabolism, modulate oxidative stress, and determine cell fate in the central nervous system [6, 7]. Mitochondrial dysfunction plays a critical role in the development and progression of ischemic stroke. Ischemic stroke disrupts the mitochondrial membrane and may induce the leakage of mitochondrial DNA (mtDNA) into the cytosol [8, 9]. Cytosolic mtDNA acts as a danger‐associated molecular pattern (DAMP), thereby triggering pattern recognition receptor (PRR)‐mediated inflammatory responses [10, 11]. For example, in models of nonalcoholic fatty liver disease and perfluorooctane sulfonate (PFOS)‐induced systemic inflammation, mtDNA activates absent in melanoma 2 (AIM2), leading to caspase‐1 activation and subsequent gasdermin D‐mediated pyroptosis [8, 9, 10, 11]. However, whether and how mtDNA leakage contributes to neuronal pyroptosis in ischemic stroke remains unclear.

Previous studies have shown that mtDNA leakage may involve the formation of Bax/Bak oligomeric pores [12], opening of the mitochondrial permeability transition pore (mPTP) [13, 14], and structural abnormalities in mitochondrial cristae [15]. Voltage‐dependent anion channel 1 (VDAC1), a major channel protein in the mitochondrial outer membrane, regulates energy metabolism and transmembrane metabolite transport [16, 17, 18, 19, 20]. Furthermore, VDAC1 oligomerization has been shown to drive mtDNA leakage in several pathological conditions, including Alzheimer's disease, systemic lupus erythematosus, and liver fibrosis [19, 21, 22, 23]. A recent study reported that ceramides trigger VDAC1 oligomerization in astrocytes during brain ischemia, leading to mtDNA release and cGAS/STING‐mediated neuroinflammation [24]. However, the regulation of VDAC1 oligomerization in neurons during ischemic stroke remains largely unexplored.

Therefore, this study aimed to investigate the molecular mechanism underlying VDAC1 oligomerization after ischemic stroke and to identify creatine kinase mitochondrial 1 (CKMT1) as a key regulatory protein. CKMT1, located in the mitochondrial intermembrane space, is a crucial enzyme involved in maintaining energy homeostasis [25, 26]. Under physiological conditions, CKMT1 forms a transmembrane complex with VDAC1 and adenine nucleotide translocase (ANT) to maintain ATP homeostasis [27]. Beyond its physiological role, CKMT1 has been implicated in several pathological models [25, 26, 28, 29, 30, 31, 32]. In ulcerative colitis, CKMT1 deficiency promotes excessive mitochondrial reactive oxygen species (ROS) production and opening of the mPTP, leading to apoptosis [31]. In acute myeloid leukemia, CKMT1 sustains mitochondrial ATP production, and its inhibition induces tumor cell apoptosis [25]. However, the role and mechanism of CKMT1 in ischemic stroke‐induced injury have not been investigated.

The present study demonstrates for the first time that CKMT1 directly inhibits VDAC1 oligomerization through steric hindrance, thereby reducing mtDNA leakage, attenuating AIM2‐mediated neuronal pyroptosis, and alleviating ischemic brain injury. These findings uncover a protective role for CKMT1 and identify the CKMT1–VDAC1–mtDNA axis as a potential therapeutic target for ischemic stroke.

## 2 Results

### 2.1 Ischemic Stroke Aggravates Neuronal Injury by Promoting VDAC1 Oligomerization‐Mediated mtDNA Leakage

Zea‐Longa scoring indicated that ischemic stroke caused pronounced neurological deficits in rats subjected to MCAO (Figure 1A). TTC staining revealed extensive cerebral infarction (Figure 1B,C). Nissl staining showed marked cortical neuronal damage in the MCAO group (Figure 1D, red arrows). Transmission electron microscopy (TEM) revealed intact mitochondrial structures with normal outer membrane and cristae morphology in the sham group (Figure 1E, yellow arrows). In contrast, mitochondria in the ischemic penumbra of MCAO rats exhibited remarkable morphological alterations characterized by swelling and cristae loss (Figure 1E, red arrows). Quantitative PCR (qPCR) analysis revealed an approximately fourfold increase in cytosolic mtDNA levels in the ischemic penumbra (Figure 1F).

**FIGURE 1:**
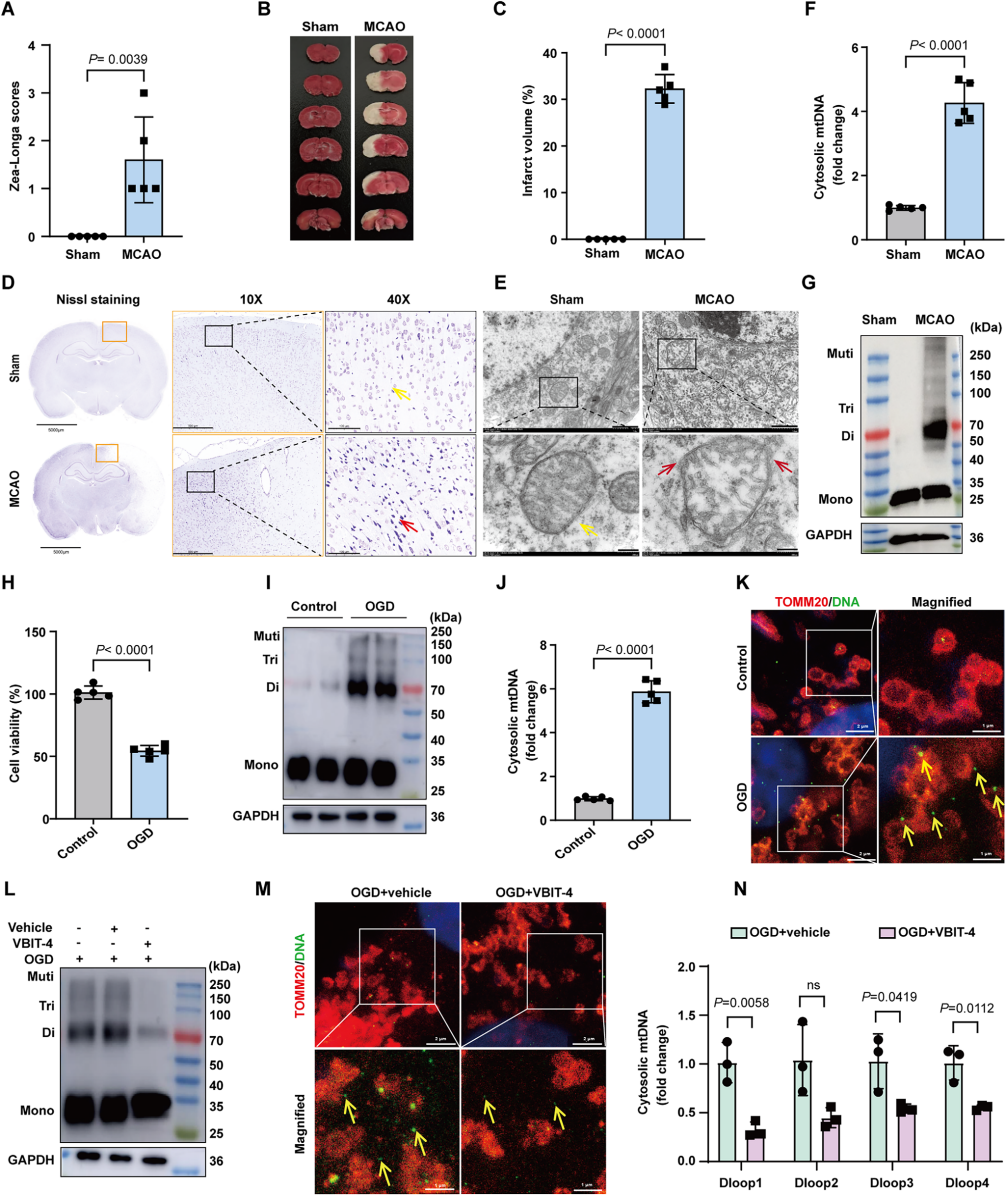
Cerebral ischemia induces VDAC1 oligomerization‐mediated mtDNA leakage in vivo and in vitro. (A) Neurological deficit scores (Zea‐Longa scale) in rats subjected to MCAO. (B) Representative TTC‐stained coronal brain sections (red, viable tissue; white, infarcted tissue). (C) Quantification of cerebral infarct volume (%) from (B). (D) Nissl staining evaluating neuronal damage in the cortical penumbra. Scale bars: 5000 µm (left), 500 µm (middle), 100 µm (right). (E) Representative transmission electron microscopy (TEM) images showing mitochondrial ultrastructure in the ischemic penumbra. Red arrows indicate disrupted cristae, whereas yellow arrows indicate intact mitochondria. Scale bars: 1 µm (top), 200 nm (bottom). (F) Cytosolic mitochondrial DNA (mtDNA) levels in the ischemic penumbra were quantified by qPCR using a D‐loop‐targeting primer and normalized to nuclear *Tert*. (G) Analysis of VDAC1 oligomerization in mitochondrial fractions isolated from the cortical penumbra of rats after MCAO using a cross‐linking assay with 200 µm ethylene glycol bis (succinimidyl succinate) (EGS), followed by immunoblotting. Mono, monomer; Di, dimer; Tri, trimer; Multi, multimer. (H) Cell viability of PC12 cells exposed to OGD was assessed by CCK‐8 assay. (I) Analysis of VDAC1 oligomerization in PC12 cells after OGD using cross‐linking with 1 mm EGS, followed by immunoblotting. Mono, monomer; Di, dimer; Tri, trimer; Multi, multimer. (J) Cytosolic mtDNA levels were measured by qPCR in PC12 cells after OGD. (K) STED super‐resolution microscopy images showing mtDNA (green) leakage from mitochondria in PC12 cells after OGD (TOMM20, red). Yellow arrows indicate leaking mtDNA. Scale bars: 2 µm (main panels), 1 µm (insets). (L) Effect of the VDAC1 oligomerization inhibitor VBIT‐4 (20 µm, 30 min pretreatment) on VDAC1 oligomerization in PC12 cells after OGD, assessed by cross‐linking assay. (M) Effect of VBIT4 on mtDNA leakage from mitochondria in PC12 cells after OGD, visualized by STED microscopy. Scale bars: 2 µm (main panels), 1 µm (insets). (N) Cytosolic mtDNA levels in PC12 cells quantified by qPCR using four independent primer sets targeting the D‐loop region and normalized to *Tert*. Data are presented as mean ± SD. Statistical significance was determined using a two‐tailed unpaired Student's t‐test (A, C, F, H, J) or one‐way ANOVA followed by Tukey's post hoc test (N). The number of biologically independent replicates (n) for each panel is as follows: *n* = 5 (A–C, F–J, L); *n* = 3 (D, E, K, M, N). Exact *P* values are shown in the figure panels.

To determine whether VDAC1 oligomerization contributes to mtDNA leakage in vivo, mitochondria were isolated from the cortical penumbra of rats after MCAO. Mitochondrial purity was confirmed by immunoblotting for VDAC1 and GAPDH (Figure S1A). Ethylene glycol bis (succinimidyl succinate) (EGS) cross‐linking assays demonstrated a marked increase in VDAC1 oligomerization in the ischemic penumbra (Figure 1G). These results indicate that ischemic stroke promotes VDAC1 oligomerization in vivo, which is accompanied by mitochondrial structural alterations and aberrant cytosolic mtDNA accumulation.

We next evaluated the role of VDAC1 oligomerization in mtDNA leakage using OGD‐treated PC12 cells. CCK‐8 assay showed that OGD significantly reduced cell viability (Figure 1H). Protein cross‐linking analysis revealed a marked increase in VDAC1 oligomerization following OGD (Figure 1I). qPCR analysis further demonstrated a significant elevation in cytosolic mtDNA levels after OGD (Figure 1J). Immunofluorescence analysis showed the relocalization of mtDNA (green, DNA staining) from mitochondria (red, TOMM20 staining) into the cytosol (Figure 1K). Pretreatment with VBIT‐4, an inhibitor of VDAC1 oligomerization [33], improved cell viability after OGD in a dose‐dependent manner, with 20 µm providing the strongest protective effect (Figure S2A). Pretreatment with 20 µm VBIT‐4 also suppressed OGD‐induced VDAC1 oligomerization (Figure 1L). Immunofluorescence analysis further showed that VBIT‐4 significantly reduced cytosolic mtDNA accumulation (Figure 1M). Among the D‐loop1–4 primer sets evaluated for amplification performance, D‐loop1 showed the best efficiency, stability, and mitochondrial specificity, which was therefore used for subsequent analyses. VBIT‐4 pretreatment significantly reduced cytosolic mtDNA levels (Figure 1N).

Collectively, these results indicate that OGD induces VDAC1 oligomerization‐dependent mtDNA leakage, thereby aggravating neuronal injury. Combined with the in vivo data, the present study demonstrates that ischemic stroke promotes VDAC1 oligomerization, which subsequently mediates mtDNA leakage and neuronal injury.

### 2.2 OGD‐Mediated Cytosolic mtDNA Leakage Triggers Neuronal Pyroptosis Through AIM2 Inflammasome Activation

ChIP‐qPCR analysis revealed significant enrichment of mtDNA in AIM2 complexes in PC12 cells after OGD (Figure 2A), whereas enrichment of nuclear genomic DNA (Rn18s) was minimal (Figure 2B), suggesting that cytosolic mtDNA may serve as a key ligand for AIM2 inflammasome activation. To further validate this hypothesis, the effects of cytosolic mtDNA on AIM2 inflammasome activation were assessed using ethidium bromide (EtBr, 250 ng/mL, 48 h), which depletes cytosolic mtDNA. Pretreatment with EtBr significantly reduced cytosolic mtDNA levels (Figure 2C and Figure S2B) and improved cell viability in PC12 cells after OGD (Figure 2D and Figure S2C).

**FIGURE 2:**
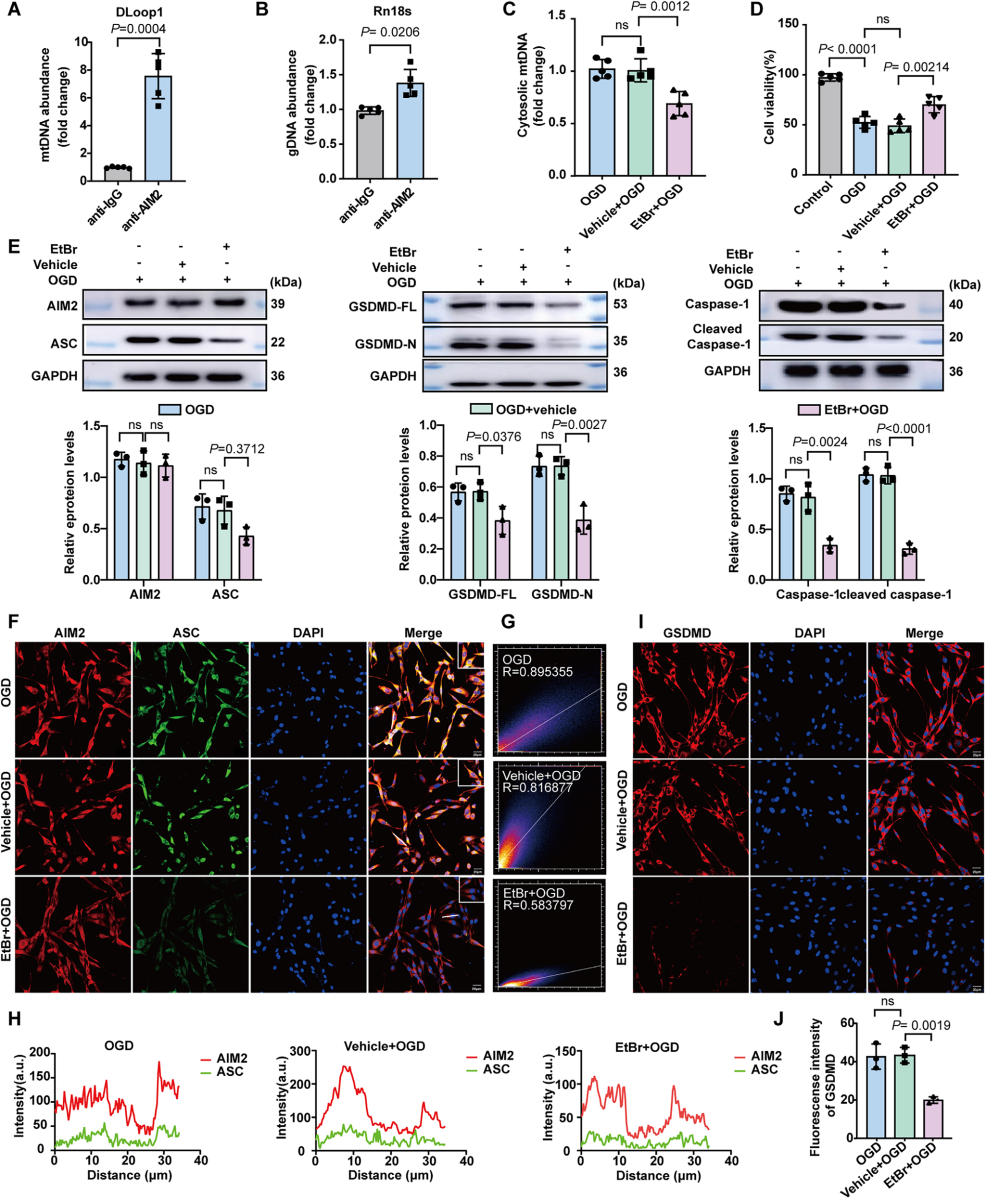
OGD‐mediated cytosolic mtDNA leakage induces neuronal pyroptosis via the AIM2 inflammasome in vitro. (A, B) Enrichment of mtDNA (A) or gDNA (B) in anti‐AIM2 immunoprecipitates from PC12 cells after OGD, assessed by ChIP‐qPCR. Relative enrichment was calculated based on %Input and normalized to the mean %Input of IgG control. (C) Viability of mtDNA‐depleted PC12 cells pretreated with 250 ng/mL EtBr for 48 h and subsequently subjected to OGD, assessed by CCK‐8 assay. (D) Validation of mtDNA depletion by quantifying cytosolic mtDNA levels in EtBr‐pretreated PC12 cells using qPCR. (E) Western blot analysis of AIM2 inflammasome components (AIM2 and ASC) and pyroptosis‐related proteins (full‐length GSDMD, cleaved GSDMD, pro‐caspase‐1, and cleaved caspase‐1) in PC12 cells after OGD with or without EtBr pretreatment. (F) Representative immunofluorescence images showing AIM2 (red) and ASC (green) in PC12 cells after OGD with or without EtBr pretreatment. Nuclei were stained with DAPI (blue). Scale bar: 20 µm. (G) Quantification of AIM2–ASC colocalization using Pearson's correlation coefficient determined by ImageJ. (H) Fluorescence intensity profiles along the white line in (F) were analyzed using ImageJ. (I, J) Representative immunofluorescence images (I) and quantitative analysis (J) of GSDMD (green) in PC12 cells after OGD with or without EtBr pretreatment. Scale bar: 20 µm. Data are presented as mean ± SD. Statistical significance was determined using a two‐tailed unpaired Student's t‐test (A, B) or one‐way ANOVA followed by Tukey's post hoc test (C–E, G, and J). Sample sizes (n) represent biologically independent cultures: *n* = 5 for A–D and *n* = 3 for E–J. Exact *P* values are shown in the figure panels.

Western blot analysis showed that EtBr pretreatment markedly downregulated ASC and pyroptosis‐related proteins, including GSDMD‐FL, GSDMD‐N, caspase‐1, and cleaved caspase‐1 after OGD, whereas AIM2 protein expression remained largely unchanged (Figure 2E). Immunofluorescence staining further confirmed that EtBr significantly reduced AIM2–ASC colocalization (Figure 2F–H) and attenuated GSDMD signal (Figure 2I,J). Collectively, these findings suggest that OGD‐induced mtDNA leakage into the cytosol activates AIM2 inflammasome‐mediated neuronal pyroptosis.

### 2.3 Inhibition of VDAC1 Oligomerization Attenuates Neuronal Pyroptosis by Reducing mtDNA Leakage In vivo

To evaluate the therapeutic effects of VBIT‐4 on cerebral ischemic injury in rats, VBIT‐4 was administered 30 min before MCAO by contralateral intracerebroventricular injection (Figure 3A). VBIT‐4 pretreatment significantly reduced neuronal cytosolic mtDNA leakage (Figure 3B) and attenuated cerebral ischemic injury in MCAO rats (Figure 3C,D).

**FIGURE 3:**
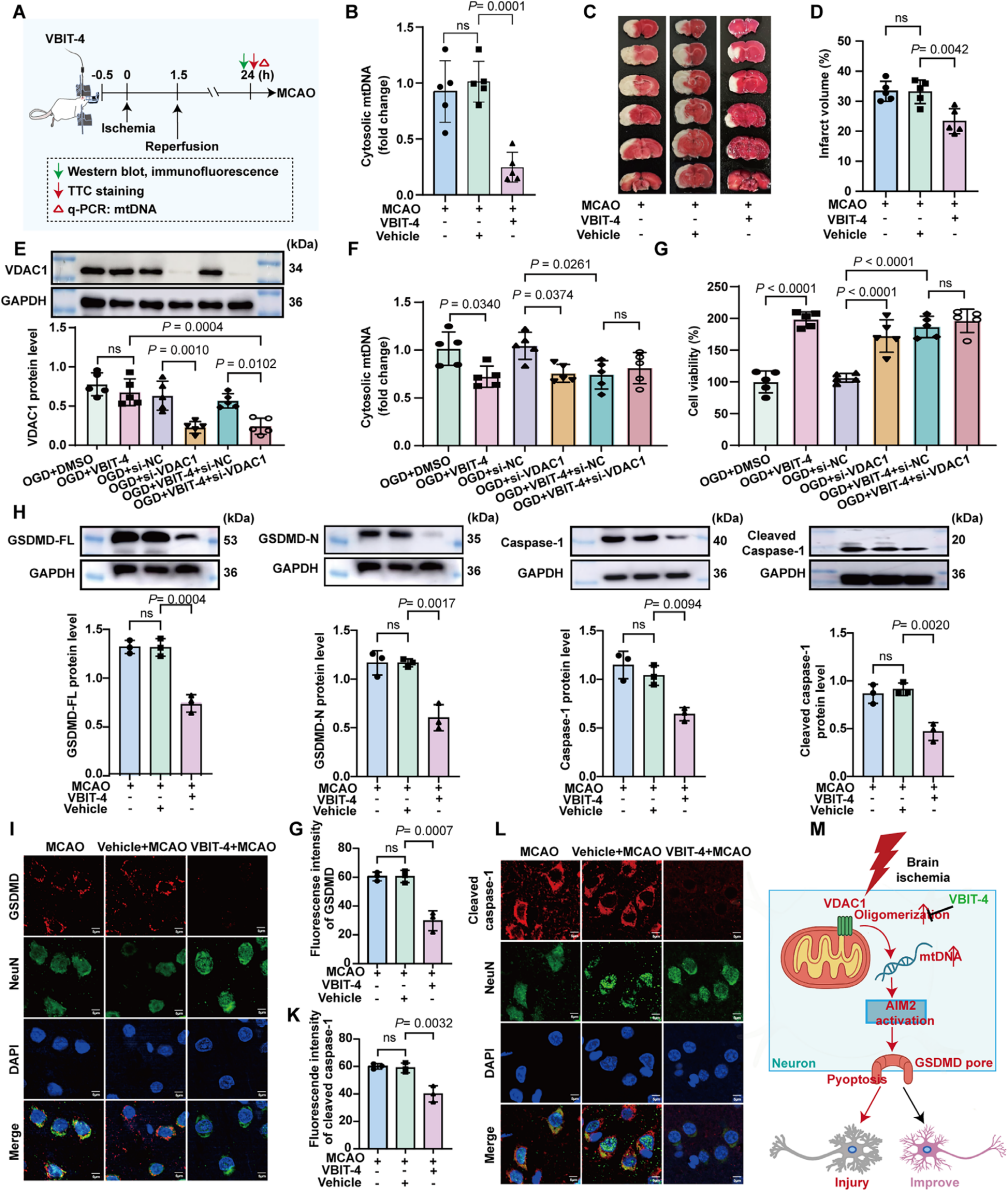
VBIT4 attenuates neuronal pyroptosis by reducing cytosolic mtDNA leakage in vivo and in vitro. (A) Experimental design and timeline of ischemic stroke induction, VBIT‐4 pretreatment, and analyses in rats. VBIT‐4 (10 µm, 5 µL) or vehicle was administered via unilateral intracerebroventricular injection 30 min before MCAO. (B) Cytosolic mtDNA levels in the cortical penumbra of rats after MCAO were quantified by qPCR. (C) Representative TTC‐stained coronal brain sections from rats after MCAO. (D) Quantification of infarct volume (%) from (C). (E) Western blot analysis of VDAC1 protein expression in PC12 cells after OGD. (F) Cytosolic mtDNA levels in PC12 cells after OGD were quantified by qPCR. (G) Cell viability in PC12 cells after OGD was assessed using the CCK‐8 assay. (H) Western blot analysis of pyroptosis‐related proteins (GSDMD, N‐GSDMD, pro‐caspase‐1, and cleaved caspase‐1) in the cortical penumbra of MCAO rats. (I–L) Representative immunofluorescence images and quantitative analysis of GSDMD (I, J) and cleaved caspase‐1(K, L) in neurons within the cortical penumbra after MCAO. Scale bar: 5 µm. (M) Schematic representation of the neuroprotective mechanism of VBIT‐4. VBIT‐4 inhibits VDAC1 oligomerization and mtDNA leakage, thereby mitigating AIM2 inflammasome‐mediated neuronal pyroptosis. Data are presented as mean ± SD. Statistical significance was determined using one‐way ANOVA followed by Tukey's post‐hoc test (B, D–H, J, and L). The number of biologically independent replicates (n) for each panel is as follows: *n* = 5 for B–H (B–D, H: animal tissues; E–G: independent cultures) and *n* = 3 for I–L (animal tissues). Exact *P* values are shown in the figure panels.

To further determine whether VDAC1 expression or oligomerization is responsible for mtDNA leakage after OGD, we compared the effects of VBIT‐4 and si‐VDAC1 treatment. Western blot analysis showed that VBIT‐4 did not significantly alter VDAC1 expression, whereas si‐VDAC1 reduced VDAC1 levels (Figure 3E). Both VBIT‐4 and si‐VDAC1 significantly decreased cytosolic mtDNA levels and improved cell viability after OGD (Figure 3F,G). Notably, si‐VDAC1 did not reduce mtDNA release when VDAC1 oligomerization was inhibited by VBIT‐4 (Figure 3F). Thus, VDAC1 oligomerization, rather than VDAC1 expression, is required for mtDNA leakage after OGD.

In the MCAO model, VBIT‐4 pretreatment significantly downregulated pyroptosis‐related proteins, including GSDMD‐FL, GSDMD‐N, caspase‐1, and cleaved caspase‐1 (Figure 3H). Immunofluorescence staining revealed markedly reduced signals for GSDMD (Figure 3I,J) and cleaved caspase‐1 (Figure 3K,L) in neurons within the ischemic penumbra. Taken together with the in vitro findings demonstrating that VBIT‐4 suppresses VDAC1 oligomerization (Figure 1L–N), we hypothesized that VBIT‐4 exerts neuroprotective effects by suppressing VDAC1 oligomerization and mtDNA leakage, thereby mitigating AIM2 inflammasome‐mediated neuronal pyroptosis (Figure 3M).

### 2.4 Identification of VDAC1‐Interacting Proteins

To identify proteins interacting with VDAC1, immunoprecipitation (IP) coupled with LC‐MS analysis was performed in primary cortical neurons subjected to OGD (Figure 4A). Mouse IgG was used as a mock control to exclude non‐specific interactions. A total of 681, 703, and 664 proteins were identified in the control, OGD, and IgG groups, respectively (Figure 4B). Venn diagram analysis revealed that 321 proteins were shared among the three groups (fold change > 1.5, *P* < 0.05) (Figure 4B and Data S2).

**FIGURE 4:**
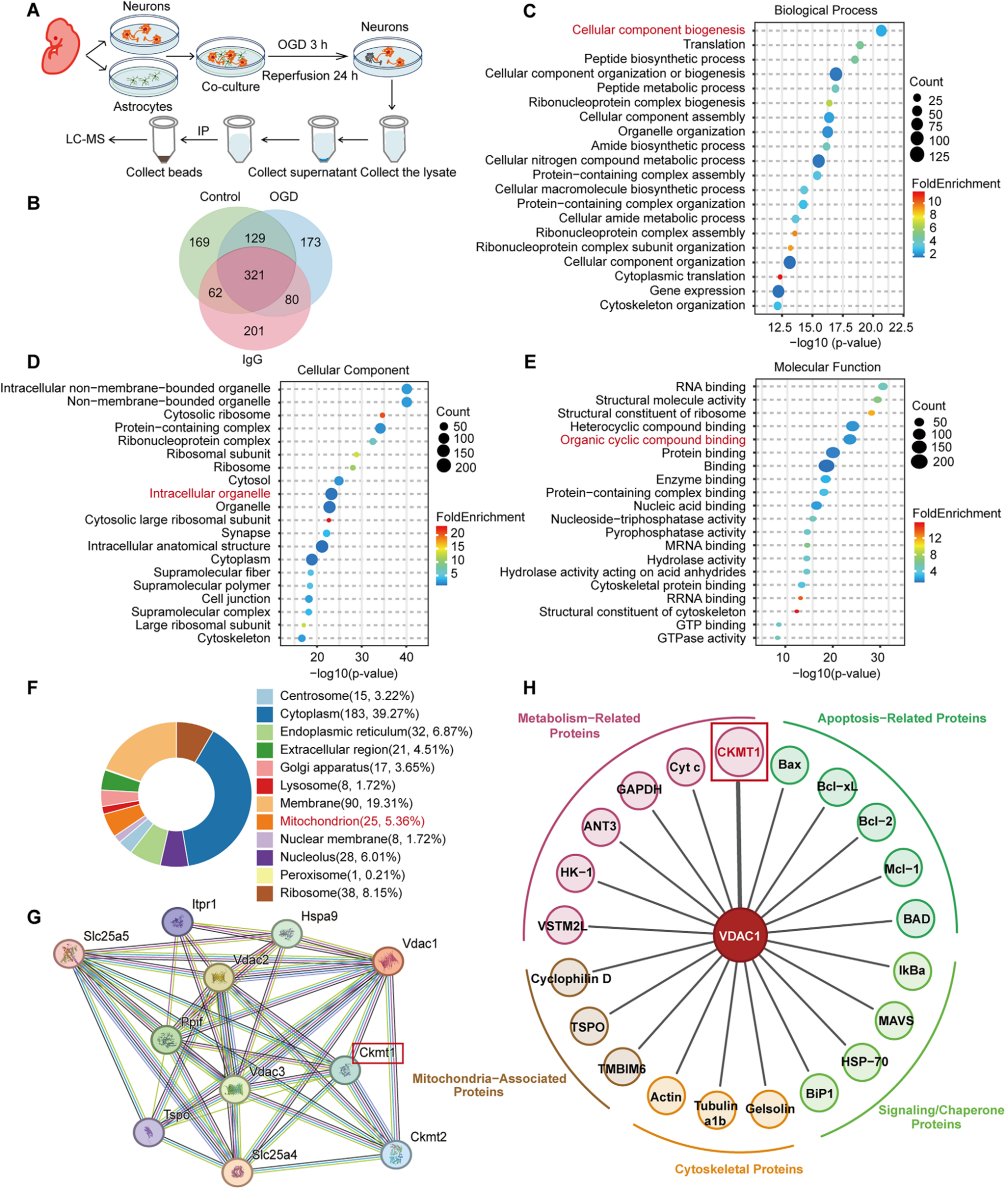
CKMT1 is a VDAC1‐interacting protein in primary neurons. (A) Experimental workflow for the co‐culture of primary cortical neurons with astrocytes from neonatal rats. Following 2 d of co‐culture and exposure to OGD/R, neurons were collected 24 h after reoxygenation for CoIP/MS to identify VDAC1‐binding partners. (B) Venn diagrams showing the numbers of unique and shared proteins identified in primary neurons among the Control, OGD, and IgG groups. (C–E) Gene Ontology (GO) enrichment analysis of the 321 shared proteins. Functional categories are shown for biological process (C), cellular component (D), and molecular function (E). (F) Subcellular localization analysis of the 321 shared proteins. (G) Protein interaction network of VDAC1 interactors generated using the STRING database, displaying the top 10 high‐confidence interactors with VDAC1 (interaction score ≥ 0.987). (H) Experimentally validated VDAC1 interaction network. Twenty‐one representative proteins with confirmed physical interactions with VDAC1 are shown and grouped according to functional category.

Gene Ontology (GO) enrichment analysis was performed using clusterProfiler (version 4.14.4) to characterize the functional profile of proteins co‐immunoprecipitated with VDAC1. Biological process analysis showed significant enrichment in cytoplasmic translation, peptide biosynthesis, and cellular component biogenesis (Figure 4C). Cellular component analysis demonstrated enrichment in the cytosolic ribosomes, intracellular organelles, and cytoplasm (Figure 4D). Molecular function analysis revealed enrichment in rRNA binding, organic cyclic compound binding, and pyrophosphatase activity (Figure 4E).

Based on the mitochondrial localization of VDAC1, a targeted screen for mitochondrial‐localized interactors identified 25 candidate proteins (Figure 4F). A VDAC1‐interaction network was generated using the STRING database, and the top 10 candidates included Slc25a4, Vdac2, Vdac3, Ppif, Hspa9, Ckmt1, Ckmt2, Itpr1, Tspo, and Slc25a5 (Figure 4G). Additionally, we summarized 21 experimentally validated VDAC1‐interacting proteins from the over 100 proteins reported by Shoshan‐Barmatz et al. [17, 18, 19, 34, 35, 36] (Figure 4H). These proteins involve key regulators of metabolism, apoptosis, and cytoskeletal organization [17, 18, 19, 34, 35, 36]. By intersecting the above 21 experimentally validated proteins with the mitochondrial‐localized proteins among the 10 proteins predicted by STRING and those enriched in specific GO categories, CKMT1 was identified as the core candidate for the VDAC1‐interacting protein.

### 2.5 Ischemic Stroke Upregulates CKMT1 and VDAC1 Expression, and Induces Disassembly of the CKMT1 High‐Molecular‐Mass Complex

Western blot analysis revealed that CKMT1 protein levels significantly increased at 24 and 48 h after reperfusion, peaked at 24 h, and returned to baseline levels at 72 h in rats subjected to MCAO. Interestingly, the protein levels of VDAC1 and the pyroptosis executors (GSDMD‐FL and GSDMD‐N) increased from 24 to 72 h and peaked at 72 h after reperfusion (Figure 5A). The protein levels of CKMT1 and VDAC1 were significantly elevated at 24 h after OGD in both primary cortical neurons and PC12 cells (Figure 5B,C). Immunofluorescence staining confirmed that CKMT1 expression was markedly elevated in the neurons within the rat ischemic penumbra after MCAO (Figure 5D,E). In addition, CKMT1 enzymatic activity was significantly enhanced in the ischemic penumbra and primary cortical neurons following MCAO/OGD (Figure 5F–H).

**FIGURE 5:**
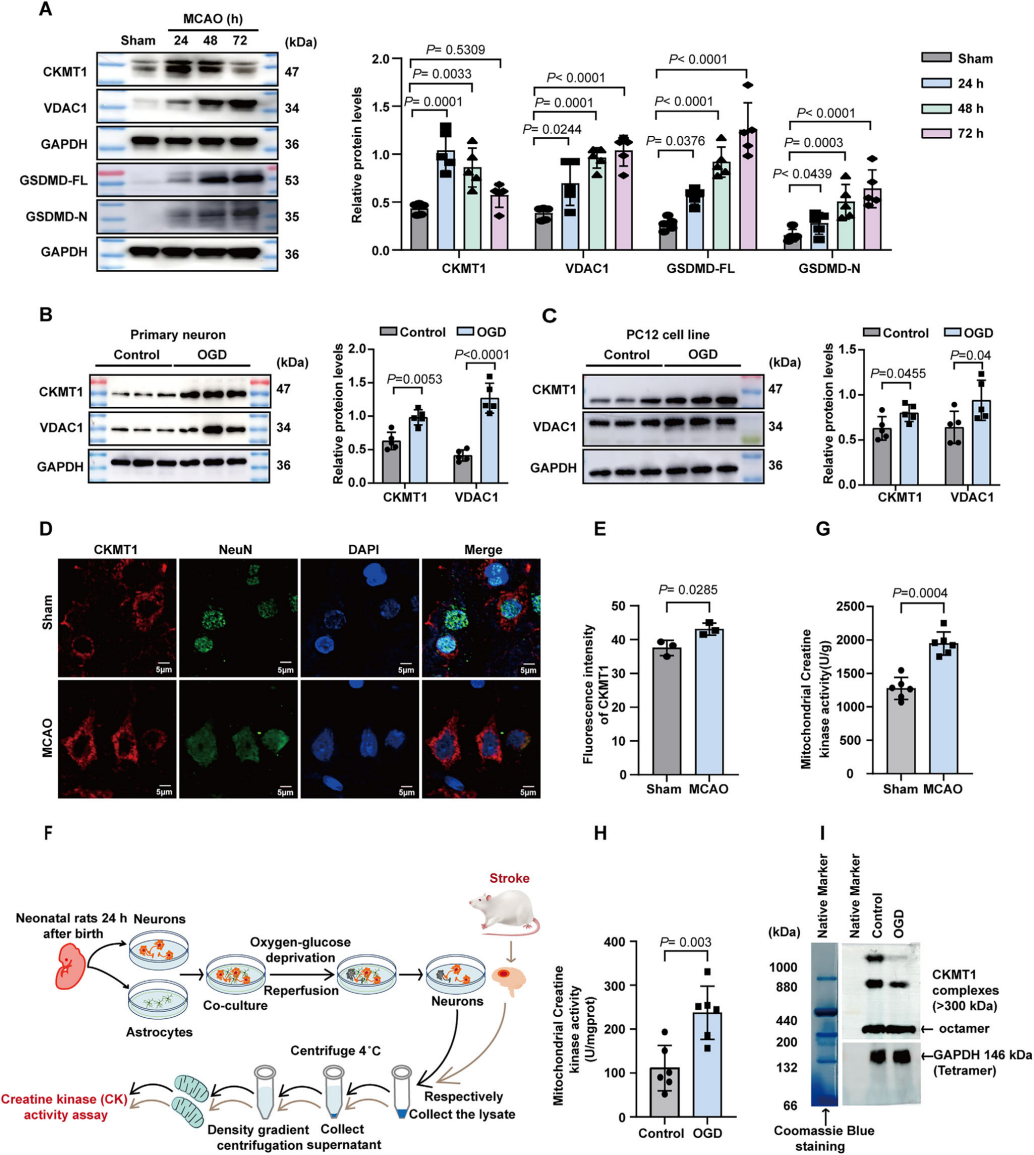
Ischemic stroke upregulates CKMT1 and VDAC1 expression, and promotes disassembly of the CKMT1 high‐molecular‐mass complex. (A) Western blot analysis of CKMT1, VDAC1, GSDMD‐FL, and GSDMD‐N protein levels in the cortical penumbra of rats at 24, 48, and 72 h after MCAO. (B, C) Western blot analysis of CKMT1 and VDAC1 protein levels in primary cortical neurons (B) and PC12 cells (C) after OGD. (D) Representative immunofluorescence images showing CKMT1 (red) and the neuronal marker NeuN (green) in the cortical penumbra of rats after MCAO. Scale bar: 5 µm. (E) Quantification of CKMT1 mean fluorescence intensity from (D). (F) Schematic diagram illustrating the experimental workflow for measuring CKMT1 enzymatic activity in primary cortical neurons and rat cortical tissue. (G) CKMT1 enzymatic activity in the cortical penumbra of rats after MCAO. (H) CKMT1 enzymatic activity in primary neurons after OGD. (I) Native PAGE analysis of CKMT1 high‐molecular‐mass complexes in PC12 cells after OGD. The CKMT1 octamer (∼336 kDa) and high‐molecular‐mass complex (>480 kDa) are presented. Data are presented as mean ± SD. Statistical significance was determined using a two‐tailed unpaired Student's t‐test (B–E, G, H). The number of biologically independent replicates (n) for each panel is as follows: *n* = 5 for A (animal tissue) and B, C, and I (independent cultures); *n* = 3 for D and E (animal tissue); *n* = 6 for G (animal tissue) and H (independent cultures). Exact *P* values are shown in the figure panels.

Previous studies have reported that CKMT1 regulates permeability transition pores through a high‐molecular‐mass complex distinct from classical VDAC1‐mediated pores [27]. Therefore, we examined whether OGD altered the assembly state of CKMT1 complexes. Native PAGE analysis revealed that the CKMT1 octamer (∼336 kDa) remained stable in PC12 cells after OGD, whereas the high‐molecular‐mass complex (>480 kDa) underwent disassembly (Figure 5I). Combined with the CKMT1 expression shown in Figure 5A, these findings indicate that ischemic stroke upregulates CKMT1 expression while simultaneously promoting the disassembly of the CKMT1 high‐molecular‐mass complex.

### 2.6 Enhanced Interaction Between VDAC1 and CKMT1 after MCAO/OGD

The interaction between CKMT1 and VDAC1 was examined using reciprocal Co‐IP assays. The results showed that the interaction was significantly enhanced in the cortical penumbra of rats following MCAO (Figure 6A,B). Immunofluorescence staining further revealed significantly increased co‐localization of CKMT1 and VDAC1 in the ischemic cortex (Figure 6C–E). In primary cortical neurons, OGD induced significant neuronal injury (Figure 6F). Immunofluorescence analysis similarly demonstrated a pronounced increase in the co‐localization of VDAC1 and CKMT1 in neurons after OGD (Figure 6G–I).

**FIGURE 6:**
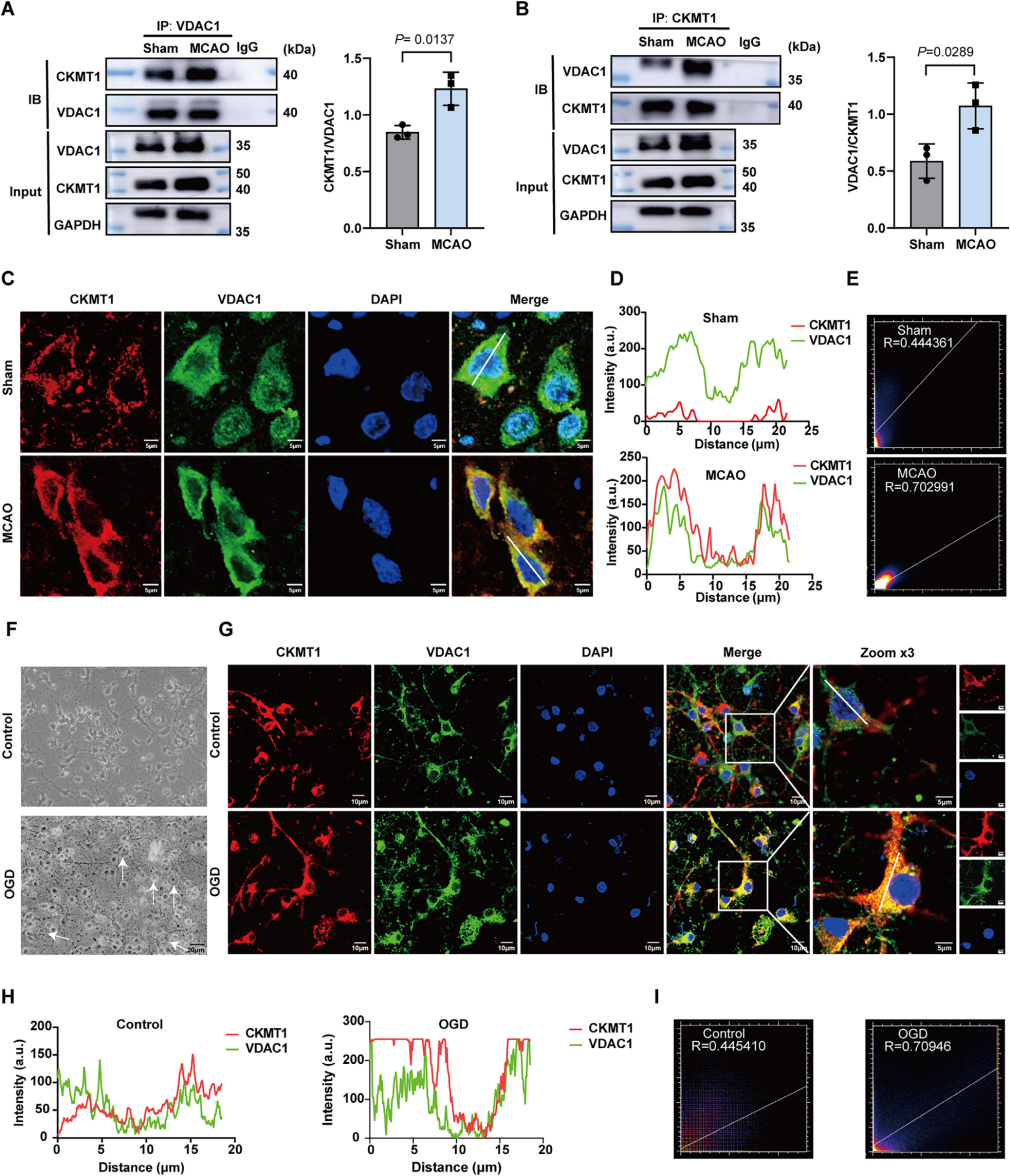
Enhanced CKMT1–VDAC1 interaction and colocalization after brain ischemia in vivo and in vitro. (A, B) Enhanced endogenous interaction between CKMT1 and VDAC1 in the cortical penumbra of rats after MCAO. Cortical lysates from rats subjected to MCAO were immunoprecipitated with anti‐VDAC1 (A) or anti‐CKMT1 (B) antibodies, followed by western blotting. Quantitative analyses are shown on the right. (C) Representative immunofluorescence images showing CKMT1 (red) and VDAC1 (green) in cortical neurons within the ischemic penumbra after MCAO. Scale bar: 5 µm. (D) Fluorescence intensity profiles along the white line in (C) were analyzed by ImageJ. (E) Quantification of CKMT1–VDAC1 colocalization using Pearson's correlation coefficient determined by ImageJ. (F) Bright‐field images of primary cortical neurons after OGD. White arrows indicate damaged neurons. Scale bar: 100 µm. (G) Representative immunofluorescence images of CKMT1 and VDAC1 in primary cortical neurons after OGD. Scale bars: 10 µm (overview), 5 µm (3 × zoom). (H) Fluorescence intensity profiles of the merged images across the white line in (G) were analyzed using ImageJ. (I) Quantification of CKMT1–VDAC1 colocalization in primary neurons after OGD using Pearson's correlation coefficient determined by ImageJ. Data are presented as mean ± SD. Statistical significance was determined using a two‐tailed unpaired Student's t‐test (A, B, E, I). The number of biologically independent replicates (n) for each panel is as follows: *n* = 5 for A and B (animal tissue); *n* = 3 for C–I (independent cultures). Exact *P* values are shown in the figure panels.

### 2.7 CKMT1 Specifically and Directly Interacts with VDAC1

To determine whether CKMT1 directly interacts with VDAC1, we first identified three CKMT1‐specific peptides in primary neurons after OGD using IP coupled with LC/MS analysis (Figure 7A), suggesting a physical interaction between CKMT1 and VDAC1. To exclude potential interference from other proteins, pcDNA3.1‐HA‐CKMT1 and pcDNA3.1‐Flag‐VDAC1 plasmids were constructed and co‐transfected into HEK293T cells to validate a direct CKMT1–VDAC1 interaction. Co‐expression of HA‐CKMT1 and Flag‐VDAC1 in HEK293T cells was confirmed by immunoblotting with anti‐CKMT1 and anti‐VDAC1 antibodies (Figure S3A). Subsequent Co‐IP confirmed a stable interaction between CKMT1 and VDAC1 in this overexpression system using anti‐HA or anti‐Flag antibodies (Figure 7B,C).

**FIGURE 7:**
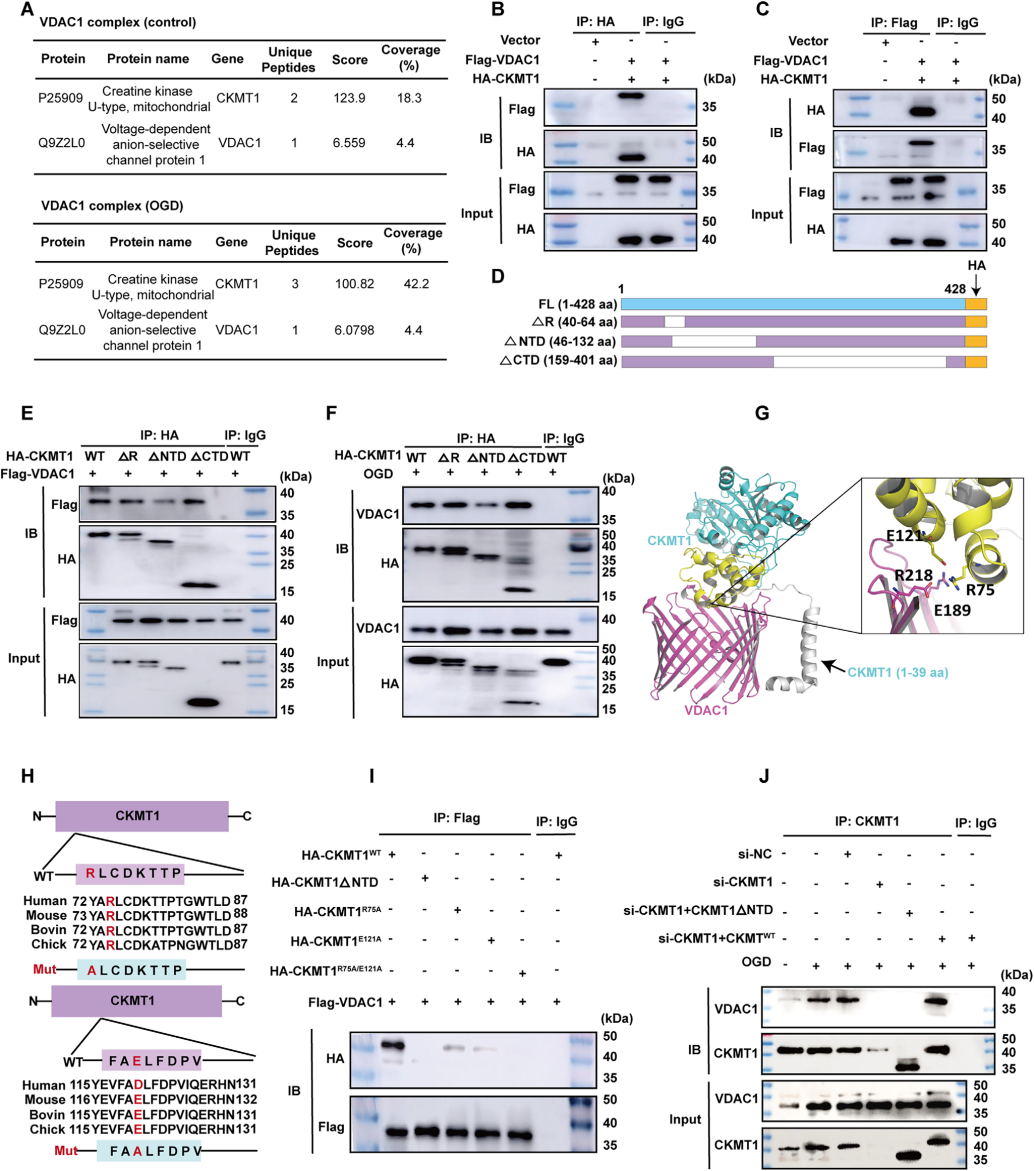
CKMT1 directly interacts with VDAC1. (A) CKMT1‐specific peptides identified in primary neurons after OGD by IP‐LC‐MS analysis. (B, C) Co‐immunoprecipitation assays confirming the direct interaction between CKMT1 and VDAC1 in HEK293T cells co‐transfected with HA‐CKMT1 and Flag‐VDAC1. Lysates were immunoprecipitated with anti‐HA (B) or anti‐Flag (C) antibodies, followed by western blotting with the indicated antibodies. (D) Schematic representation of full‐length CKMT1 (CKMT1‐FL) and its truncated mutants (ΔR, ΔNTD, and ΔCTD). (E) HEK293T cells were co‐transfected with Flag‐VDAC1 and either CKMT1‐FL or the truncated mutants (ΔR, ΔNTD, and ΔCTD). Cell lysates were immunoprecipitated with anti‐HA antibodies to determine the VDAC1 binding region of CKMT1. (F) PC12 cells were transfected with CKMT1‐FL or the truncated mutants (ΔR, ΔNTD, and ΔCTD), followed by OGD treatment. Cell lysates were immunoprecipitated with anti‐HA antibodies to determine the VDAC1 binding region of CKMT1. (G) Predicted three‐dimensional interaction model between CKMT1 (yellow) and VDAC1 generated using AlphaFold Server. The model predicts that the NTD of CKMT1 docks onto VDAC1 and potentially forms salt bridges involving CKMT1‐E121/VDAC1‐R218 and CKMT1‐R75/VDAC1‐E189. (H) Conservation analysis of the CKMT1–VDAC1 interaction site across species and schematic of the CKMT1 mutants (R75A, E121A, R75A/E121A) generated based on the rat CKMT1 sequence. (I) Validation of critical interaction residues. HEK293T cells were co‐transfected with Flag‐VDAC1 and either CKMT1‐FL, the ΔNTD mutant, or the indicated point mutants (R75A, E121A, R75A/E121A). Cell lysates were immunoprecipitated with anti‐Flag antibodies to determine the key site for the interaction between CKMT1 and VDAC1. (J) PC12 cells were transfected with si‐CKMT1 to suppress endogenous CKMT1 expression, and subsequently co‐transfected with either CKMT1‐ΔNTD or CKMT1‐FL plasmids for 48 h before OGD treatment. Cell lysates were immunoprecipitated with an anti‐CKMT1 antibody and immunoblotted for VDAC1 to evaluate the role of the N‐terminal domain in CKMT1–VDAC1 binding after OGD. Sample sizes (n) represent biologically independent cultures: *n* = 5 for B and C; *n* = 3 for E, F, I, and J.

To identify the domain responsible for this interaction, Flag‐VDAC1 was co‐expressed in HEK293T cells with either full‐length CKMT1 (CKMT1‐FL) or truncated mutants (ΔR, ΔNTD, ΔCTD) (Figure 7D). Co‐IP assays showed that VDAC1 interacted with CKMT1‐FL and specifically bound to the N‐terminal domain (NTD, residues 46–132 aa) (Figure 7E). The NTD of CKMT1 also played a major role in mediating its interaction with VDAC1 in PC12 cells following OGD (Figure 7F). AlphaFold structural analysis suggested that VDAC1 directly interacts with the NTD of CKMT1 through electrostatic interactions. This interaction is stabilized by critical salt bridges involving key residue pairs in CKMT1‐E121/VDAC1‐R218 and CKMT1‐R75/VDAC1‐E189, which are predicted to promote complex stability (Figure 7G). Conservation analysis revealed that R75 is highly conserved across species, whereas E121 shows interspecies variability. Notably, this residue corresponds to aspartate (D) in human CKMT1 (Figure 7H). Functional validation using site‐directed mutagenesis (R75A, E121A, and R75A/E121A) of CKMT1 demonstrated that mutation of either residue markedly weakened the binding between CKMT1 and VDAC1 (Figure 7I). Collectively, these findings indicate that the NTD (46–132 aa) of CKMT1 is the principal domain mediating direct binding to VDAC1, with R75 and E121 being the key stabilizing residues.

To further determine whether the N‐terminal domain of CKMT1 mediates VDAC1 binding during OGD, si‐CKMT1 was co‐transfected with either CKMT1‐ΔNTD or CKMT1‐FL in PC12 cells. OGD significantly increased the amount of VDAC1 co‐immunoprecipitated with CKMT1. This OGD‐induced enhancement of the CKMT1–VDAC1 interaction was abolished by si‐CKMT1. Importantly, restoring CKMT1 expression with CKMT1‐FL rescued the interaction, whereas CKMT1‐ΔNTD failed to do so (Figure 7J). These results demonstrate that the enhanced CKMT1–VDAC1 interaction during OGD depends on the N‐terminal domain of CKMT1.

### 2.8 CKMT1 Suppresses VDAC1 Oligomerization via Steric Hindrance

The effects of CKMT1 knockdown and overexpression on VDAC1 oligomerization were assessed in PC12 cells. High transfection efficiency of the small interfering RNA (siRNA) was confirmed by enhanced green fluorescent protein (EGFP) (Figure 8A). qRT‐PCR and western blot analyses demonstrated that transfection with CKMT1‐specific siRNA significantly downregulated CKMT1 expression in PC12 cells (Figure S4A,B). CKMT1 knockdown exacerbated VDAC1 oligomerization and mtDNA leakage, thereby reducing cell viability following OGD (Figure 8B–D). These effects were partially reversed by treatment with the VDAC1 oligomerization inhibitor VBIT‐4 (Figure 8E). Subsequently, a CKMT1 overexpression plasmid was successfully transfected into PC12 cells (Figure 8F). qRT‐PCR and western blot analyses confirmed significant upregulation of CKMT1 expression (Figure S4C,D). Further analysis demonstrated that CKMT1 overexpression significantly attenuated OGD‐induced VDAC1 oligomerization, mtDNA leakage, and cellular injury (Figure 8G–I).

**FIGURE 8:**
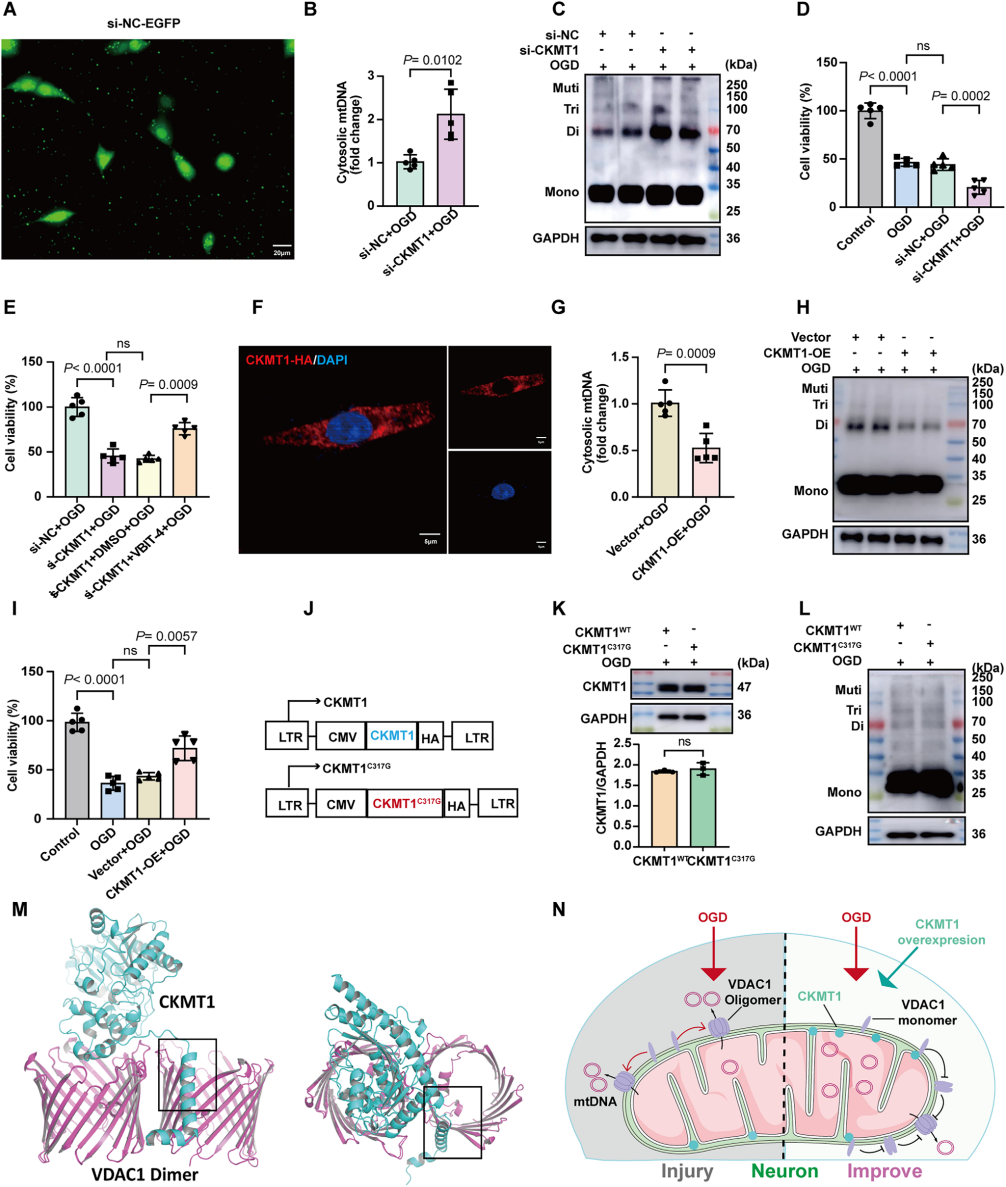
CKMT1 inhibits VDAC1 oligomerization via steric hindrance to reduce mtDNA leakage and neuronal injury after OGD. (A) Representative immunofluorescence image of PC12 cells 48 h after transfection with siNC. Scale bar: 20 µm. (B) Effect of CKMT1 knockdown on cytosolic mtDNA levels in PC12 cells after OGD, assessed by qPCR. (C) Effect of CKMT1 knockdown on VDAC1 oligomerization by cross‐linking assay. (D) Effect of CKMT1 knockdown on cell viability assessed using the CCK‐8 assay. (E) Effect of the VDAC1 oligomerization inhibitor VBIT‐4 on cell viability assessed using the CCK‐8 assay. (F) Immunofluorescence staining showing HA‐CKMT1 (red) and nuclei stained with DAPI (blue) in PC12 cells transfected with HA‐CKMT1 plasmid. Scale bar: 5 µm. (G) Effect of CKMT1 overexpression on cytosolic mtDNA levels in PC12 cells after OGD. (H) Effect of CKMT1 overexpression on VDAC1 oligomerization assessed by a cross‐linking assay. (I) Effect of CKMT1 overexpression on cell viability. (J) Schematic of WT CKMT1 and the catalytic‐dead mutant C317G. (K) Western blot analysis of CKMT1 protein expression in PC12 cells transfected with WT or C317G mutant plasmids after OGD. (L) Effect of transfection with CKMT1 WT or C317G mutant plasmids on VDAC1 oligomerization in PC12 cells after OGD, assessed by a cross‐linking assay. (M) Structural model illustrating docking of the CKMT1 signal peptide (amino acids 1–39) at the VDAC1 dimer interface, suggesting a potential steric hindrance mechanism. (N) Effect of CKMT1 Δ1‐39 on VDAC1 oligomerization in PC12 cells after OGD, assessed by cross‐linking assay. (O) Effect of CKMT1 Δ1‐39 on cell viability assessed using the CCK‐8 assay. (P) Schematic illustration of CKMT1‐mediated inhibition of VDAC1 oligomerization. CKMT1 overexpression inhibits VDAC1 dimerization and subsequent oligomerization, thereby reducing mtDNA leakage and attenuating cellular injury. Data are presented as mean ± SD. Statistical significance was determined using a two‐tailed unpaired Student's t‐test (B, G, K) or one‐way ANOVA followed by Tukey's post hoc test (D, E, I, and O). Sample sizes (n) represent biologically independent cultures: *n* = 5 for B–E, G–I, L, N, and O; *n* = 3 for A, F, and K. Exact *P* values are shown in the figure panels.

To investigate whether the enzymatic activity of CKMT1 regulates VDAC1 oligomerization, a catalytic site mutant (C317G) was constructed (Figure 8J). Western blot analysis revealed no significant difference in CKMT1 expression between the C317G mutant and wild‐type (WT) in PC12 cells after OGD (Figure 8K). Protein cross‐linking assays demonstrated that both WT and C317G CKMT1 exerted comparable inhibitory effects on VDAC1 oligomerization (Figure 8L), indicating that CKMT1‐mediated regulation of VDAC1 oligomerization is independent of its catalytic activity.

Based on the interaction between VDAC1 and the NTD (46–132 aa) of CKMT1, structural modeling further revealed that residues 1–39 of CKMT1 occupied a key spatial position at the VDAC1 dimerization interface, thereby sterically hindering VDAC1 dimerization by blocking β‐barrel assembly and obstructing subsequent oligomerization (Figure 8M). To further validate the functional role of residues 1–39, a CKMT1‐Δ1‐39 mutant was constructed and transfected into PC12 cells (Figure S5A). Overexpression of CKMT1‐WT significantly suppressed OGD‐induced VDAC1 oligomerization, whereas CKMT1‐Δ1‐39 failed to inhibit the oligomerization (Figure 8N). Consistently, CKMT1‐WT improved cell viability after OGD, while CKMT1‐Δ1‐39 aggravated cellular injury (Figure 8O). IP‐LC‐MS analysis identified peptides from residues 1–39 of CKMT1 within VDAC1 complexes (Figure S6A), further supporting the proposed steric blockade mechanism mediated by CKMT1 in a VDAC1 dimer. Collectively, these data demonstrate that CKMT1 inhibits VDAC1 oligomerization through a “dual‐region synergy” mechanism, in which the NTD binds to VDAC1, while residues 1–39 sterically hinder VDAC1 dimerization, thereby suppressing its oligomerization and protecting neurons against OGD‐induced neuronal injury (Figure 8P).

### 2.9 Neuron‐Specific Overexpression of CKMT1 Attenuates Ischemic Brain Injury In vivo

To investigate the role of CKMT1 in ischemic brain injury, CKMT1 was specifically overexpressed in rat neurons prior to MCAO (Figure 9A). Western blot analysis confirmed significant upregulation of CKMT1 protein expression (Figure S7A). In the MCAO model, neuron‐specific overexpression of CKMT1 significantly reduced cerebral infarct volume (Figure 9B), suppressed the expression of AIM2 and pyroptosis‐related proteins (Figure 9C), and decreased the number of damaged neurons in the cortical penumbra (Figure 9D). Behavioral assessments further demonstrated that CKMT1 overexpression improved spontaneous locomotor activity, as evidenced by increased total travel distance and locomotion time (Figure 9E,F), and ameliorated forelimb asymmetry in the cylinder test (Figure 9G). Collectively, these findings indicate that CKMT1 overexpression alleviates cerebral ischemic injury and improves motor function, thereby exerting neuroprotective effects in rats.

**FIGURE 9:**
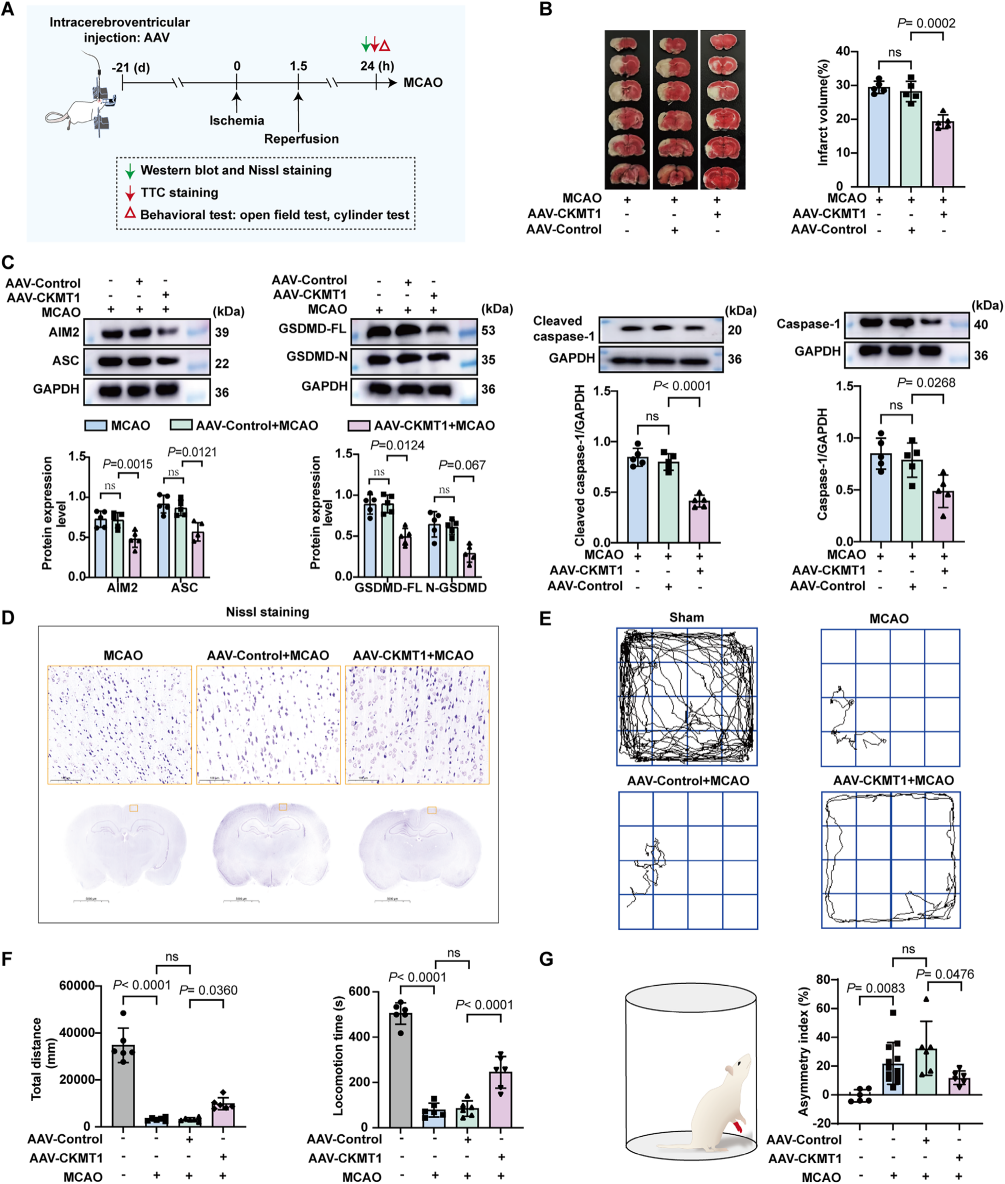
Neuron‐specific overexpression of CKMT1 attenuates ischemic brain injury in vivo. (A) Schematic timeline of the experimental procedure involving intracerebroventricular injection of neuron‐specific adeno‐associated virus (AAV)‐CKMT1 or AAV‐control 21 days before MCAO, followed by subsequent analyses. (B) Representative TTC‐stained brain sections and quantification of infarct volume in rats after MCAO. (C) Western blot analysis of AIM2 inflammasome components (AIM2 and ASC) and pyroptosis‐related proteins (GSDMD, N‐GSDMD, pro‐caspase‐1, and cleaved caspase‐1) in the cortical penumbra of rats after MCAO. (D) Nissl staining evaluating neuronal damage in the cortical penumbra after MCAO. Scale bars: 100 µm (upper panel), 5 mm (lower panel). (E) Representative locomotor trajectories of rats after MCAO in the open‐field test. (F) Quantification of total distance traveled and locomotion time in the open‐field test. (G) Asymmetry index derived from forelimb use during the cylinder test in rats after MCAO. Data are presented as mean ± SD. Statistical significance was determined using one‐way ANOVA followed by Tukey's post hoc test (B, C, F, G). The number of biologically independent replicates (n) for each panel is as follows: *n* = 5 for B and C (animal tissue); *n* = 3 for D (animal tissue); *n* = 6 for E–G (animal tissue). Exact *P* values are shown in the figure panels.

## 3 Discussion

The present study demonstrates that CKMT1 exerts a neuroprotective effect against cerebral ischemic injury by directly binding to VDAC1 and inhibiting its oligomerization. Neuron‐specific overexpression of CKMT1 attenuated VDAC1 oligomerization, thereby suppressing pathological mtDNA leakage into the cytosol, inhibiting AIM2 inflammasome activation and subsequent pyroptosis, and ultimately ameliorating ischemic brain injury (Figure 10).

**FIGURE 10:**
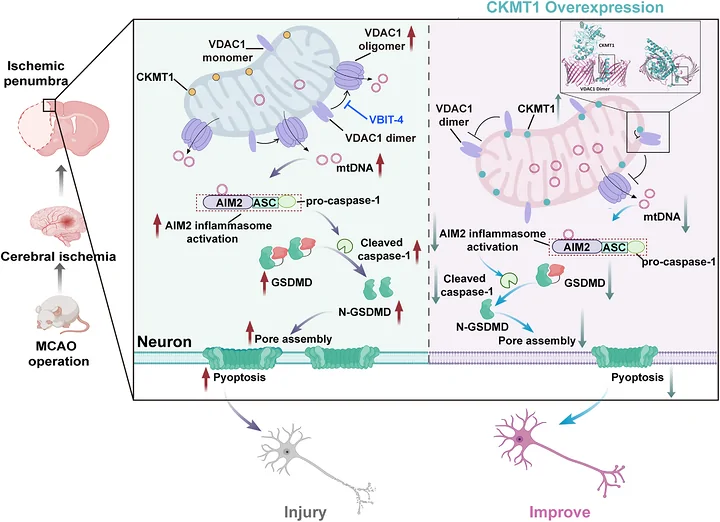
Schematic illustration of the protective mechanism of CKMT1 against ischemic brain injury through direct binding to VDAC1. CKMT1 overexpression inhibits VDAC1 oligomerization, thereby reducing mtDNA leakage and suppressing AIM2 inflammasome activation. This ultimately attenuates neuronal pyroptosis and protects against ischemic brain injury (Created with BioRender.com).

Mitochondrial dysfunction is a central pathological event in ischemic brain injury. VDAC1 is a key channel protein embedded in the mitochondrial outer membrane (MOM) that regulates mitochondrial membrane permeability and mediates downstream damage signaling through conformational changes under pathological conditions [2, 6, 7, 17, 19, 37, 38]. In sepsis and neurodegenerative diseases, VDAC1 oligomerization forms transmembrane pores that facilitate mtDNA leakage into the cytosol, thereby inducing pyroptosis [22, 23, 24, 39]. Inhibition of VDAC1 oligomerization or reduction of its expression has been shown to alleviate cellular damage [37, 38, 40, 41].

In the present study, we demonstrated that ischemic stroke induced neurological deficits, structural alteration of the MOM, and cytosolic accumulation of mtDNA in neurons after MCAO. Notably, VDAC1 oligomerization was significantly enhanced in the rat cortical penumbra after MCAO. Further in vitro models using OGD‐treated cells showed significant upregulation of VDAC1 expression and oligomerization, accompanied by mtDNA leakage into the cytosol. Pretreatment with VBIT‐4 markedly attenuated OGD‐induced mtDNA leakage and improved neuronal survival. Similarly, si‐VDAC1 reduced mtDNA leakage and improved neuronal survival. Importantly, when VDAC1 oligomerization was already inhibited by VBIT‐4, si‐VDAC1 failed to further reduce mtDNA leakage, indicating that VDAC1 oligomerization, rather than VDAC1 expression, is the dominant driver of mtDNA release. These findings suggest that increased VDAC1 expression expands the pool of monomers available for oligomerization, whereas oligomer formation directly mediates mtDNA leakage.

Collectively, these findings indicate that ischemic stroke aggravates neuronal injury by promoting VDAC1 oligomerization, thereby facilitating mtDNA leakage from mitochondria. Consistent with our results, Qi et al. reported that elevated long‐chain ceramide levels promote VDAC1 oligomerization in astrocytes, leading to mtDNA leakage and ischemic brain injury [24]. VDAC1 has also been implicated in neuroinflammation associated with Alzheimer's disease [42, 43] and epilepsy [44, 45]. However, changes in VDAC1 expression following ischemic stroke remain controversial. Wan et al. reported increased VDAC1 expression in neurons after OGD, consistent with our findings [38], whereas Yao et al. observed downregulation of VDAC1‐3 in the hippocampal CA1 region after global ischemic stroke [46]. These discrepancies may reflect differences in ischemic models (focal vs. global ischemia) and the brain regions examined.

Based on these findings, we further investigated downstream pathways activated by cytosolic mtDNA leakage. We found that mtDNA was significantly enriched in AIM2‐containing complexes in neurons after OGD, indicating a specific interaction between mtDNA and AIM2. Moreover, OGD‐induced upregulation of ASC and other pyroptosis‐related proteins was markedly inhibited by ethidium bromide (EtBr), which depleted cytosolic mtDNA, whereas AIM2 expression remained unaffected. EtBr pretreatment also significantly reduced AIM2‐ASC colocalization. These findings indicate that cytosolic mtDNA activates the AIM2 inflammasome by promoting its assembly. This observation is consistent with the canonical activation mechanism of the AIM2 inflammasome, in which cytosolic DNA binds the AIM2 HIN domain to initiate inflammasome formation [8, 11, 47, 48]. Previous studies have shown that mtDNA activates the AIM2 inflammasome in models of nonalcoholic fatty liver disease and PFOS‐induced multiple organ inflammation [11, 49]. In addition, AIM2 and ASC contribute to acute ischemic brain injury [50], and AIM2 inhibition alleviates brain injury and cognitive deficits [51, 52, 53]. Although previous reports demonstrated that oxidized mtDNA release during ischemic stroke activates the cGAS–STING pathway and drives neuroinflammation [54], whether mtDNA leakage also activates the AIM2 inflammasome to mediate brain ischemic injury remained unclear.

We further investigated the effects of inhibiting VDAC1 oligomerization on mtDNA leakage and neuronal pyroptosis in vivo. Pretreatment with VBIT‐4 markedly attenuated ischemic neuronal damage, accompanied by significant reductions in cytosolic mtDNA levels and pyroptosis‐related protein expression. These findings are consistent with previous studies showing that VBIT‐4 suppresses VDAC1 oligomerization, reduces mtDNA leakage, and alleviates inflammatory responses in lupus‐like disease [23, 55]. Together, these findings establish VDAC1 oligomerization as a key pathological mechanism across multiple diseases [19, 34, 35, 46, 47, 48]. Importantly, our study demonstrates for the first time that ischemia‐induced neuronal VDAC1 oligomerization promotes mtDNA leakage, activates AIM2 inflammasome‐mediated pyroptosis, and ultimately exacerbates ischemic brain injury.

To elucidate the upstream regulatory mechanism of VDAC1 oligomerization, IP‐LC‐MS was conducted to identify proteins interacting with VDAC1 in OGD‐treated primary cortical neurons. Extensive evidence from Shoshan‐Barmatz et al. has demonstrated that VDAC1 physically interacts with more than 100 proteins, including actin, tubulin, Bcl‐2, Bcl‐xL, and hexokinase [17, 18, 19, 34]. By integrating the experimentally validated VDAC1 interactors with our IP‐LC‐MS data and STRING‐predicted interaction network, we identified CKMT1 as a core candidate for further investigation.

Under physiological conditions, CKMT1 forms a mitochondrial transmembrane energy complex with VDAC1 and ANT to maintain cellular energy balance [27]. Dolder et al. further demonstrated that CKMT1 forms a complex with VDAC1 at mitochondrial contact sites to regulate energy homeostasis and mPTP‐induced oxidative damage [56]. However, the role of CKMT1 in cerebral ischemic injury has remained unclear. In the present study, CKMT1 expression was significantly increased after MCAO/OGD, and both the interaction and co‐localization of VDAC1 and CKMT1 were markedly enhanced. These findings suggest that CKMT1 directly regulates VDAC1 function through direct binding.

To determine the molecular basis of this interaction, we mapped the CKMT1 binding interface using structural domain analysis. CKMT1 contains three domains: a region (R, 40–64 aa) involved in binding with cardiolipin and anchoring in the inner mitochondrial membrane, an N‐terminal domain (NTD, 46–132 aa) containing catalytic sites, and a C‐terminal domain (CTD, 159–401 aa) that completes the catalytic core [56, 57]. Our data identified the NTD (46–132 aa) as the principal domain responsible for VDAC1 binding. OGD enhanced the CKMT1–VDAC1 interaction, whereas si‐CKMT1 abolished this enhancement. Importantly, re‐expression of CKMT1 lacking the NTD failed to restore the interaction, confirming that the NTD is required for OGD‐induced CKMT1–VDAC1 binding.

Native PAGE analysis further revealed that the CKMT1 high‐molecular‐mass complex disassembled during OGD. This is consistent with a previous report by Datler et al., showing that CKMT1 forms a high‐molecular‐mass complex distinct from the canonical octamer, which disintegrates to regulate mPTP during cytotoxic stress [27]. Notably, disassembly of the CKMT1 complex in our study was accompanied by increased CKMT1–VDAC1 binding and inhibition of VDAC1 oligomerization, suggesting that disintegrated CKMT1 exerts an endogenous protective effect by increasing interaction with VDAC1.

A point‐mutation experiment further identified CKMT1 (R75 and E121) as key residues that form stabilizing salt bridges with VDAC1 (R218 and E189) through electrostatic complementation. Conservation analysis demonstrated that R75 is highly conserved across species, whereas E121 is more variable, suggesting that R75 may be indispensable for this interaction, while E121 may modulate species‐specific binding affinity. Together, these findings identify a direct binding interface between CKMT1 and VDAC1 and provide a structural basis for further mechanistic investigations and therapeutic targeting.

CKMT1 has been implicated in multiple pathological conditions [25, 26, 28, 29, 30, 31, 32]. For example, peptidyl arginine deiminase 4 (PAD4)‐mediated citrullination of CKMT1 impairs mitochondrial function and exacerbates inflammation in bowel disease [32], while CKMT1 deficiency promotes excessive mitochondrial ROS and mPTP opening, leading to cell apoptosis in ulcerative colitis [31]. In acute myeloid leukemia, CKMT1 supports mitochondrial ATP production and cell survival [25]. However, its role in cerebral ischemic injury had not previously been investigated.

In OGD‐treated PC12 cells, CKMT1 knockdown markedly aggravated neuronal injury by enhancing VDAC1 oligomerization and mtDNA leakage. Conversely, neuron‐specific overexpression of CKMT1 significantly suppressed VDAC1 oligomerization and mtDNA leakage both in vitro and in vivo, thereby exerting robust neuroprotective effects. Interestingly, mutagenesis of the catalytic site demonstrated that CKMT1 enzymatic activity was not required for inhibition of VDAC1 oligomerization, indicating that this protective mechanism is structurally rather than enzymatically mediated.

To further elucidate the molecular mechanism underlying CKMT1‐mediated inhibition of VDAC1 oligomerization, we performed AlphaFold‐based structural modeling. After docking of the CKMT1 NTD onto the VDAC1 C‐terminal domain, the models revealed that residues 1–39 of CKMT1 occupy a critical spatial position at the VDAC1 dimerization interface. Deletion of residues 1–39 abolished the inhibitory effect of CKMT1 on VDAC1 oligomerization and eliminated its neuroprotective effect during OGD. These findings demonstrate that residues 1–39 are essential for CKMT1‐mediated suppression of VDAC1 oligomerization through steric hindrance.

Recent studies have shown that uncleaved signal peptides can directly regulate protein oligomerization under pathological conditions [34, 58], such as the SpA signal peptide inhibiting PepV multimerization [59], supporting our finding that the CKMT1 signal peptide (residues 1–39) sterically hinders VDAC1 dimerization. Thus, CKMT1 inhibits VDAC1 oligomerization through a dual‐region mechanism: the NTD forms a stable interaction interface with the VDAC1 C‐terminal domain, while residues 1–39 sterically obstruct VDAC1 dimer and oligomerization formation.

VDAC1 consists of a 19‐stranded β‐barrel pore lumen and a highly mobile N‐terminal α‐helix that plays a pivotal role in oligomerization and pore formation [60, 61, 62, 63]. Geula et al. identified β‐strands 8 and 16 as key oligomerization interfaces [57]. In contrast, our study demonstrates that the VDAC1 C‐terminal domain also functions as a critical regulatory site for oligomerization. Specifically, CKMT1 inhibits VDAC1 oligomerization by binding residues R218 and E189 and sterically blocking β‐barrel assembly. These findings therefore extend and complement the model proposed by Geula et al. [57].

Our findings are further supported by Gao et al. [36], who demonstrated that the mitochondrial protein V‐set and transmembrane domain‐containing protein 2‐like (VSTM2L) inhibits VDAC1 oligomerization by binding the VDAC1 C‐terminus and preventing dissociation from hexokinase 2, thereby alleviating ferroptosis in a prostate cancer model. Collectively, these observations highlight the complexity of VDAC1 oligomerization regulation.

Notably, CKMT1 and VDAC1 exhibited distinct temporal expression patterns after MCAO. CKMT1 expression increased rapidly and peaked at 24 h before declining to baseline by 72 h, whereas VDAC1 expression increased progressively and peaked at 72 h. These findings suggest that ischemic stroke triggers a transient compensatory upregulation of endogenous CKMT1 protein, but this response is insufficient to counteract sustained VDAC1 oligomerization and pyroptosis. Importantly, exogenous CKMT1 overexpression inhibited VDAC1 oligomerization and neuronal pyroptosis, suggesting that enhancing CKMT1 activity may represent a promising strategy for cerebral ischemic injury.

Nevertheless, some limitations should be acknowledged. Future studies should exploit the newly identified interaction sites to design synthetic small‐molecule peptides capable of specifically inhibiting VDAC1 oligomerization, thereby providing potential translational strategies for ischemic stroke treatment.

## 4 Conclusions

Our findings demonstrate that CKMT1 protects against cerebral ischemic injury by directly interacting with VDAC1 and inhibiting its oligomerization, thereby reducing mtDNA leakage and AIM2 inflammasome‐mediated neuronal pyroptosis. These findings establish CKMT1 as a promising therapeutic target for cerebral ischemic injury from both functional and structural perspectives. Furthermore, this work provides a precise molecular framework for the development of targeted therapies aimed at the CKMT1–VDAC1 complex and offers new strategic insights into the treatment of ischemic brain injury.

## 5 Methods

### 5.1 Animals

Male Sprague‐Dawley (SD) rats (8 weeks old, 250–280 g) were obtained from Beijing HFK Bioscience Co., Ltd. (Beijing, China). All animals were housed under specific pathogen‐free (SPF) conditions at a controlled temperature (20°C–26°C) and humidity (40%–60%) under a 12 h light/dark cycle. All experimental procedures involving animals were reviewed and approved by the Institutional Animal Care and Use Committee (IACUC) of Hebei Medical University (Approval No. LACUC‐Hebmu‐PD‐201918, Shijiazhuang, China).

For euthanasia, rats were exposed to gradually increasing concentrations of carbon dioxide (CO_2_) from compressed gas cylinders in strict accordance with the AVMA Guidelines for the Euthanasia of Animals. All efforts were made to minimize animal suffering, and no abuse or maltreatment occurred during the study period.

### 5.2 Middle Cerebral Artery Occlusion (MCAO) Model

A focal ischemic stroke model was established in SD rats by left MCAO, as previously described [64, 65]. Briefly, anesthesia was induced via intraperitoneal injection of 2,2,2‐tribromoethanol (15 mg/kg). Body temperature was maintained at 37°C ± 0.5°C throughout the procedure using a thermostatically controlled heating pad.

The left common, internal, and external carotid arteries were isolated and ligated. A silicon‐coated nylon suture (model 2634A4; Beijing Xinong Technology Co., Ltd.) was inserted into the internal carotid artery and advanced until mild resistance was detected (approximately 18–20 mm from the bifurcation) to occlude the origin of the middle cerebral artery. After 90 min of occlusion, the filament was withdrawn to restore blood flow. Sham‐operated animals underwent the same surgical procedure without filament insertion. After surgery, rats were allowed to recover on a 37°C heating pad until fully awake.

### 5.3 2, 3, 5‐Triphenyl Tetrazolium Chloride (TTC) Staining

At 24 h after reperfusion, rats were euthanized, and the brains were harvested. Brain tissues were cooled briefly at –20°C for 10–15 min and sectioned coronally into 2 mm slices. The slices were immersed in 2% TTC solution (Cat. No. SL7140, Coolaber, China) and incubated at 37°C in the dark for 20 min with gentle turning every 10 min. Subsequently, the sections were fixed in 4% paraformaldehyde (PFA) for 24 h. Viable tissue was stained red, whereas infarcted tissue remained pale.

### 5.4 Nissl Staining

Nissl staining was performed as previously described [66]. Briefly, serial 5 µm brain sections were stained with cresyl violet and immersed in 1% toluidine blue solution for 40 min. After rinsing with deionized water, the sections were differentiated in 70% alcohol for 5 min. Cortical regions from three animals per group were examined histologically. High‐resolution whole‐slide imaging was performed using a digital slide scanner (Pannoramic SCAN; 3D HISTECH; Hungary) for subsequent quantitative analysis.

### 5.5 Western Blot

Proteins were isolated from ischemic cortical penumbra or cultured cells after oxygen‐glucose deprivation (OGD). Briefly, samples were lysed in RIPA buffer and centrifuged at 12 000 rpm for 15 min at 4°C, after which the supernatants were collected. Protein concentrations were measured using a BCA protein assay kit.

After denaturation in 5 × loading buffer at 95°C for 5 min, equal amounts of protein (30 µg) were separated on 12% Tris‐Glycine gels and electrotransferred onto polyvinylidene difluoride (PVDF) membranes (Merck Millipore). Membranes were blocked with 5% skim milk at room temperature for 1.5 h and incubated overnight at 4°C with primary antibodies (details provided in Data S1). After washing with TBST, membranes were incubated with appropriate HRP‐conjugated secondary antibodies for 1 h at 37°C. Protein bands were visualized using an ECL kit and captured using an AI600 imaging system.

### 5.6 Cell Culture and OGD Model

OGD models were established in primary neuron‐astrocyte co‐cultures and PC12 cells to simulate ischemic stroke in vitro, as previously described [67]. Briefly, cells were seeded in 6‐ or 24‐well plates. For the co‐culture OGD model, cells were rinsed with PBS, cultured in glucose‐free medium, and exposed to hypoxic conditions (gas mixture: 94% N_2_, 1% O_2_, 5% CO_2_) for 4 h. Reoxygenation was performed by replacing the medium with normal DMEM and transferring the cells to normoxic conditions (95% air, 5% CO_2_) for 24 h.

PC12 cells were subjected to a similar procedure. Cells were cultured in complete DMEM. For OGD treatment, they were rinsed with PBS and incubated in glucose‐free DMEM under hypoxic conditions (94.9% N_2_, 0.1% O_2_, 5% CO_2_) for 15 h, followed by reoxygenation in normal DMEM under normoxic conditions for 24 h. Control cells were maintained in normal medium under normoxic conditions throughout the experiment. All cells were harvested after reoxygenation for subsequent analyses.

### 5.7 Co‐Immunoprecipitation Coupled with Liquid Chromatography‐Mass Spectrometry (Co‐IP/LC‐MS)

Cell pellets were harvested and resuspended in 500 µL lysis buffer. After manual homogenization, lysates were centrifuged at 12 000 × g for 15 min at 4°C. For Co‐IP, supernatants were incubated with 4 µg of the indicated antibodies for 12 h at 4°C. The lysates were then incubated with 25 µL protein A/G‐agarose beads (Thermo Scientific, # 88804) overnight at 4°C. After washing three times with cold lysis buffer, bead‐bound proteins were processed for mass spectrometry or mixed with 1 × loading buffer for western blot analysis.

To determine whether the NTD of CKMT1 mediates its binding to VDAC1 during OGD, si‐CKMT1 was co‐transfected into PC12 cells with either CKMT1‐ΔNTD or CKMT1‐FL constructs. Lysates were immunoprecipitated with anti‐CKMT1 antibody and immunoblotted for VDAC1 as described above.

For IP/MS analysis, bead‐bound protein complexes were denatured and reduced in SDT buffer containing TCEP and CAA at 100°C for 5 min. Samples were alkylated and buffer‐exchanged using UA buffer in a 10 kDa molecular weight cutoff (MWCO) ultrafiltration device by centrifugation at 12 000 × g. Following subsequent washes with 100 µL UA buffer (centrifuged at 12 000 g for 10 min, repeated twice) and 50 mm NH_4_HCO_3_ (centrifuged at 12 000 × g for 10 min, repeated twice), trypsin was added. The mixture was briefly oscillated and incubated at 37°C for 16–18 h. Digested peptides were collected by centrifugation, desalted using C18 StageTips, dried under vacuum, and reconstituted in 0.1% formic acid (FA) for LC‐MS analysis.

LC‐MS/MS was performed using an Easy‐nLC 1200 system (Thermo Scientific) coupled online to a Q‐Exactive HF‐X mass spectrometer (Thermo Scientific). Peptides were first loaded onto a trap column (C18, 5 µm, 100 µm × 20 mm, Dr. Maisch GmbH) and then separated on an analytical column (C18, 3 µm, 75 µm × 150 mm, Dr. Maisch GmbH) at a flow rate of 300 nL/min using a 60‐min linear gradient. Raw MS spectra data were searched against the UniProt Rattus norvegicus database using MaxQuant software (version 2.4.14.0). Search parameters included a precursor mass tolerance of 10 ppm, fragment mass tolerance of 0.02 Da, carbamidomethylation of cysteine as a fixed modification, oxidation of methionine as a variable modification, and a maximum of two missed cleavages. Peptide‐spectrum matches and proteins were filtered at a false discovery rate (FDR) <1.0%. Only proteins containing at least one unique peptide were retained. Gene Ontology (GO) enrichment analysis was performed for functional annotations. Identified proteins and peptides are listed in Data S2.

### 5.8 Transmission Electron Microscopy (TEM)

To analyze mitochondrial ultrastructure, brain tissues from sham‐operated and MCAO rats were fixed in ice‐cold 2.5 % glutaraldehyde for 2–4 h at 4°C. Samples were pre‐embedded in 1 % agarose and post‐fixed with 2% ferrocyanide‐reduced osmium tetroxide for 2 h at room temperature in the dark, followed by three PBS washes. The dehydrated tissues were embedded in resin and sectioned into 60–80 nm slices. Sections were stained with 2% uranyl acetate in saturated ethanol for 8 min in the dark, washed sequentially with 70% alcohol and ultrapure water, and counterstained with 2.6% lead citrate for 8 min. Images were acquired using a transmission electron microscope.

### 5.9 VDAC1 Protein Cross‐Linking Assay

The VDAC1 cross‐linking assay was performed as previously described with minor modifications [23, 68]. Briefly, cells were collected, washed twice with cold PBS, and centrifuged at 3000 × g for 5 min at 4°C. Cell pellets were resuspended in 2 mL of 1 mm ethylene glycol bis (EGS) diluted in DPBS and incubated for 20 min at 37°C. The reaction was quenched by adding 200 µL of 20 mm Tris‐HCL (pH 7.8) and incubating for 15 min at room temperature. Cells were subsequently lysed, and protein concentrations were determined using the BCA assay. Protein samples (50 µg) were separated by 12% SDS‐PAGE and analyzed by immunoblotting using the anti‐VDAC1 antibody.

For in vivo experiments, mitochondrial fractions were isolated from the cortical penumbra 24 h after MCAO using a commercial mitochondrial isolation kit (Abcam). Isolated mitochondria were resuspended in 500 µL mitochondria storage buffer, and EGS was added to a final concentration of 200 µm. After incubation at 30°C for 20 min, the reaction was quenched, and samples were processed for immunoblotting.

### 5.10 Immunofluorescence (IF) Staining

Immunofluorescence staining was performed on paraffin‐embedded brain sections and cultured cells (primary neurons and PC12 cells) as previously described [69], with minor modifications. For brain tissues, rats were perfused with PBS followed by 4% PFA at 24 h after MCAO or sham surgery. Brains were fixed in 4% PFA, dehydrated through graded ethanol, cleared in xylene, and embedded in paraffin. Coronal sections (5 µm) were deparaffinized, rehydrated, and subjected to antigen retrieval in citrate buffer (pH 6.0) using microwave heating. After blocking with 10% BSA for 1 h at 37°C, sections were incubated overnight at 4°C with primary antibodies, followed by fluorescent‐conjugated secondary antibodies for 1 h at 37°C. Nuclei were counterstained with DAPI.

For cultured cells, primary neurons and PC12 cells were grown on glass coverslips to approximately 60% confluence, fixed with 4% PFA for 15 min at room temperature, and blocked with 10% BSA for 1 h at 37°C. Primary and secondary antibody incubations were performed as described above. Nuclei were stained using mounting medium containing DAPI. Images were acquired using an Olympus confocal microscope and analyzed using ImageJ software.

### 5.11 Intracerebroventricular Injections

AAV delivery into the right lateral ventricle was performed under stereotaxic guidance in anesthetized rats (2% isoflurane, 2 L/min O_2_). Following a midline scalp incision, a burr hole was drilled at coordinates 1.5 mm lateral and 0.8 mm posterior to the bregma. A 10 µL syringe was inserted to a depth of 3.8–4.0 mm. Animals received 6 µL of either AAV9‐Syn‐CKMT1 (3.45 × 10^1^ ^2^ vg/mL; Heyuan Biotech) or AAV9‐sh‐NC (2.75 × 10^1^ ^3^ vg/mL; Heyuan Biotech) at a rate of 0.5 µL/min. After injection, the needle was left in place for 10 min before withdrawal. After wound closure with sutures, animals recovered on a 37°C heating pad until fully awake.

### 5.12 Mitochondrial Fractionation and CKMT1 Activity Assay

Mitochondrial fractions were isolated from cortical penumbra tissues of MCAO rats and OGD‐treated primary neurons using a commercial mitochondrial isolation kit (Abcam). CKMT1 activity was determined at 37°C using a coupled enzymatic assay measuring NADPH production at 340 nm. The 200 µL reaction mixture contained 10 mm phosphocreatine, 2 mm ADP, 10 mm glucose, 0.5 mm NADP^+^, coupling enzymes, and approximately 50 µg of mitochondrial protein. Specific activity was expressed as µmol NADPH min^−^^1^ mg^−^^1^ protein.

### 5.13 Native PAGE Analysis

CKMT1 high‐molecular‐mass complexes were analyzed by blue native PAGE (BN‐PAGE) as previously described [27, 70]. Briefly, proteins extracted from control and OGD‐treated PC12 cells were lysed and separated on Native PAGE 3%–12% Bis‐Tris gel (Thermo Fisher Scientific). Proteins were transferred onto PVDF membranes and immunoblotted with anti‐CKMT1 antibody.

### 5.14 Nuclear and Cytoplasmic Fractionation

Cytoplasmic and nuclear fractions were isolated using a commercial nuclear/cytoplasmic fractionation kit. Fresh brain tissues from the ischemic penumbra were rinsed with PBS, minced, and homogenized in 500 µL of ice‐cold Reagent A (HR0241, Bjbalb) supplemented with protease inhibitor cocktails. PC12 cells were trypsinized, pelleted, and resuspended in the same buffer.

Samples were rotated at 2°C–8°C for 30 min and centrifuged at 500 × g for 5 min at 4°C. Supernatants were collected as the cytoplasmic fraction. Pellets were washed once with ice‐cold PBS and centrifuged at 2000 × g for 5 min at 4°C to obtain nuclear fractions. Both fractions were immediately processed for DNA extraction or stored at –80°C.

### 5.15 Quantification of Cytosolic Mitochondrial DNA Content

Single‐cell suspensions were prepared from brain tissues of MCAO rats and OGD‐treated PC12 cells [71]. Each sample was divided into two aliquots for total DNA extraction or cytosolic fraction isolation using a nuclear/cytoplasmic fractionation kit. Total and cytosolic DNA were extracted using a commercial kit (TIANGEN). Mitochondrial DNA (mtDNA; D‐loop) and nuclear DNA (gDNA; *Tert*) were quantified by qPCR. Relative cytosolic mtDNA levels were normalized to total gDNA. Primer sequences are listed in Data S2.

### 5.16 Quantification of AIM2 Bound Mitochondrial and Genomic DNA

DNA bound to AIM2 was quantified using a modified chromatin immunoprecipitation (ChIP) assay [11]. Cell lysates were cross‐linked with 1% formaldehyde and sonicated to generate DNA fragments of 300–500 bp. Immunoprecipitation was performed overnight at 4°C using anti‐AIM2 antibody, with normal IgG serving as a negative control (Beyotime, Cat. No. #P2078).

After reversal of cross‐linking, DNA was purified (Beyotime, Cat. No. #D0033) and analyzed by qPCR using primers specific for mitochondrial and nuclear DNA regions. The percentage of input DNA (%Input) was calculated as 100 × 2^(Ct input‐Ct IP)^. Relative enrichment was calculated by dividing the %Input value of each IP sample by the mean %Input of IgG control replicates. Primer sequences are listed in Data S2.

### 5.17 Cell Viability Assay

Cell viability was measured using the Cell Counting Kit‐8 (CCK‐8; MCE, China) according to the manufacturer's protocol. Briefly, cells were seeded into 96‐well plates at a density of 1 × 10^4^ cells/well. After treatment, 10 µL CCK‐8 reagent was added to each well containing 100 µL serum‐free DMEM. Absorbance at 450 nm was measured using a microplate reader.

### 5.18 Plasmid Construction and siRNA Transfection

Full‐length rat CKMT1 and VDAC1 cDNAs were synthesized and subcloned into the pcDNA3.1 expression vector (Heyuan Biotech, Shanghai, China). Plasmid transfection into PC12 and HEK293T cells was performed using Lipofectamine 3000 (Vazyme) according to the manufacturer's protocol.

For RNA interference experiments, CKMT1‐specific small interfering RNA (si‐CKMT1) and a non‐targeting control siRNA (si‐NC) were designed and synthesized by General Biosystems (Anhui, China). Sequences are provided in Data S3. PC12 cells were transfected using GP Transfect‐Mate reagent (GenePharma).

For combined VBIT‐4 and si‐VDAC1 treatment experiments, PC12 cells were pre‐transfected with si‐NC or si‐VDAC1 for 48 h, pretreated with VBIT‐4 (20 µm) or DMSO for 30 min, and then subjected to OGD. VDAC1 expression, cytosolic mtDNA levels, and cell viability were subsequently analyzed as described above.

The CKMT1‐Δ1‐39 mutant was generated by deleting residues 1–39 from the full‐length CKMT1 and subcloned into pcDNA3.1. PC12 cells were transfected with empty vector, CKMT1‐WT, or CKMT1‐Δ1‐39 for 48 h prior to OGD. VDAC1 oligomerization and cell viability were assessed as described above.

### 5.19 RNA Extraction and Quantitative RT‐PCR

Total RNA was isolated from PC12 cells using TRIzol reagent (Cat. No. R401, Vazyme, Jiangsu, China). Reverse transcription was performed using HiScript III RT SuperMix. Quantitative real‐time PCR was conducted using SYBR Green Master Mix on a Bio‐Rad CFX Maestro system (Bio‐Rad, USA). All reactions were performed in duplicate. Relative gene expression levels were calculated using the 2^(−ΔΔCt) method. Primer sequences are provided in Data S3.

### 5.20 Molecular Docking Analysis

To investigate the direct interaction between CKMT1 and VDAC1 and its impact on VDAC1 dimerization, AlphaFold3‐based protein‐protein complex prediction was used to generate a CKMT1–VDAC1 binding model. Structural analyses were performed to identify key interaction residues and the binding interface.

Electrostatic interactions between CKMT1 and VDAC1 were analyzed, and the predicted CKMT1–VDAC1 complex was compared with the VDAC1 dimer structure to evaluate steric hindrance at the dimerization interface. Structural templates were disabled to enforce de novo prediction. Model quality was assessed using predicted local distance difference test (pLDDT) and predicted aligned error (PAE) scores [72]. Complex confidence was evaluated using ipTM + iTM, with values > 0.75 considered indicative of high‐confidence protein‐protein interactions [73].

### 5.21 Bioinformatic Analysis

Proteins significantly enriched in the IP‐MS experiment were converted to official gene symbols using the UniProt ID mapping tool. Gene Ontology (GO) enrichment analysis was performed for biological processes, cellular components, and molecular function using the R package ClusterProfiler (version 4.14.4) with default parameters (Fold Enrichment > 1.5). GO terms with adjusted *P* < 0.05 after Benjamini‐Hochberg correction were considered statistically significant. Enrichment results were visualized as bubble plots using the BioCloud online platform (http://47.103.152.113/BioCloud/start.php).

### 5.22 Behavioral Analysis

#### 5.22.1 Open Field Test

Locomotor activity was assessed in a square arena (80 cm × 80 cm) under standard illumination (∼50 lux). Each rat was placed in the center of the arena and allowed to explore freely for 10 min. Movement trajectories were recorded and analyzed using automated tracking software (Shanghai Jiliang Software Technology Co., Ltd., Shanghai, China) to quantify total distance traveled and locomotion time.

#### 5.22.2 Cylinder Test

Motor function and forelimb use asymmetry were assessed using the cylinder test [74]. Rats were placed individually in a cylinder (20 cm diameter × 40 cm height) and recorded for 5 min. During vertical exploration, cylinder wall contacts were recorded and categorized as follows: contralateral forelimb (unimpaired), ipsilateral forelimb (impaired), or both forelimbs contacts.

The asymmetry index (AI) was calculated as follows:

$$\begin{array}{ccc} & & \mathrm{AI}=(\mathrm{unimpaired}\;\mathrm{forelimb}\;\mathrm{contacts}\;-\;\mathrm{impaired}\;\mathrm{forelimb}\;\mathrm{contacts})\;/\\ & & \;(unimpaired\;forelimb\;contacts+impaired\;forelimb\;contacts\\ & & +\;both\;forelimb\;contacts) \end{array}$$

An AI value close to zero indicated symmetrical forelimb use, whereas larger absolute values indicated greater asymmetry. All behavioral tests were performed 24 h after ischemic stroke reperfusion.

### 5.23 Statistical Analysis

All experiments were conducted with at least three independent biological replicates. Data were normalized to the appropriate control group for each experiment, and no data were excluded from the analyses. Statistical analyses were performed using GraphPad Prism software (version 10.1.0). Data distribution was assessed using the Shapiro‐Wilk normality test, and homogeneity of variance was evaluated using Levene's test or the F‐test. Sample sizes (n) are specified in figure legends. Continuous variables are presented as mean ± standard deviation (SD). Differences between two groups were analyzed using a two‐tailed unpaired Student's t‐test. Comparisons among multiple groups were analyzed using one‐way ANOVA followed by Tukey's honest significant difference (HSD) post hoc test or the Kruskal–Wallis test followed by Dunn's post hoc test. Statistical significance was defined as a two‐tailed *P* < 0.05. Exact *P* values are provided in the figure legends.

## Author Contributions

M.Z., L.Y.Z., and L.H.D. conceived and designed the study. X.Y.W., J.H.W., H.F.Y., L.Z., X.Y.L., and Z. K.Y. performed the experiments. S.S. performed the molecular docking analysis. X.Y.W. performed the data analysis and drafted the manuscript. M.Z., L.Y.Z., J.G.Z., Y.Y.H., S.Q.L., C.G.Z., D.M., X.H.X., and W.B.L. reviewed and revised the manuscript. L.Z.L. was responsible for the management and maintenance of laboratory instruments and equipment.

## Funding

This work was supported by the National Natural Science Foundation of China (No. 82571472), the 2025 Hebei Universities‐Shijiazhuang Industry‐University‐Research Collaboration Project (No. 2517901907A), the Hengrui Hebei Innovative Development Medical Cooperation Program (No. HR202502004), the Natural Science Foundation of Hebei Province (No. H2024206037), the Science Research Project of the Hebei Education Department (No. QN2025212), the directors’ funds (No. NV20250012), and the Graduate Zhiyuan Fund (No. NV20250026) from the Key Laboratory of Neural and Vascular Biology, Ministry of Education, Hebei Medical University, China.

## Conflicts of Interest

The authors declare no conflicts of interest.

## Supporting information




**Supporting File 1**: advs78611‐sup‐0001‐SuppMat.xlsx.

**Supporting File 2**: advs78611‐sup‐0002‐SuppMat.xlsx.

**Supporting File 3**: advs78611‐sup‐0003‐SuppMat.xlsx.

**Supporting File 4**: advs78611‐sup‐0004‐SuppMat.xlsx.

**Supporting File 5**: advs78611‐sup‐0005‐SuppMat.docx.

**Supporting File 6**: advs78611‐sup‐0006‐SuppMat.pdf.

**Supporting File 7**: advs78611‐sup‐0007‐SuppMat.pdf.

## Data Availability

The mass spectrometry proteomics data have been deposited in the ProteomeXchange Consortium via the iProX partner repository [75, 76] under dataset identifier PXD073623 (http://proteomecentral.proteomexchange.org/cgi/GetDataset?ID = PXD073623). The Gene Ontology enrichment analysis results are provided in Supplementary Data 2. All remaining data are available within the article, Supplementary Information, or Source Data files. Raw data underlying all figures, including uncropped western blot images and original microscopy images, are provided in the Supporting Information.

## References

[1] E. J. Benjamin , M. J. Blaha , S. E. Chiuve , et al., “Heart Disease and Stroke Statistics‐2017 Update: A Report From the American Heart Association,” Circulation 135, no. 10 (2017): e146–e603, 10.1161/CIR.0000000000000485. PMC5408160 28122885

[2] M. Zhang , Q. Liu , H. Meng , et al., “Ischemia‐Reperfusion Injury: Molecular Mechanisms And Therapeutic Targets,” Signal Transduction and Targeted Therapy 9, no. 1 (2024): 12, 10.1038/s41392-023-01674-2. PMC10772178 38185705

[3] S. Li , L. Zhang , J. Lin , et al., “LncRNA BIRF Promotes Brain Ischemic Tolerance Induced By Cerebral Ischemic Preconditioning Through Upregulating GLT‐1 via Sponging miR‐330‐5p,” Molecular Neurobiology 59, no. 7 (2022): 3996–4014, 10.1007/s12035-022-02841-3. PMC9167204 35451738

[4] L. Zhao , S. Li , X. Wang , et al., “The AGEs/RAGE Signaling Pathway Regulates NLRP3‐Mediated Neuronal Pyroptosis After MCAO Injury in Lepr^−^/^−^ Obese Rats,” Journal of Inflammation Research 17, no. 17 (2024): 6935–6954, 10.2147/JIR.S476458. PMC11453143 39372588

[5] T. Kosugi and K. Kawahara , “Reversed Actrocytic GLT‐1 During Ischemia Is Crucial To Excitotoxic Death Of Neurons, But Contributes To The Survival Of Astrocytes Themselves,” Neurochemical Research 31, no. 7 (2006): 933–943, 10.1007/s11064-006-9107-2. 16830212

[6] R. She , D. Liu , J. Liao , G. Wang , J. Ge , and Z. Mei , “Mitochondrial Dysfunctions Induce PANoptosis and Ferroptosis In Cerebral Ischemia/Reperfusion Injury: From Pathology To Therapeutic Potential,” Frontiers in Cellular Neuroscience 17 (2023): 1191629, 10.3389/fncel.2023.1191629. PMC10244524 37293623

[7] M. Scognamiglio , V. Maresca , A. Basile , et al., “Phytochemical Characterization, Antioxidant, and Anti‐Proliferative Activities of Wild and Cultivated Nigella damascena Species Collected in Sicily (Italy),” Antioxidants 13, no. 4 (2024): 402, 10.3390/antiox13040402. PMC11047655 38671850

[8] C. R. Lammert , E. L. Frost , C. E. Bellinger , et al., “AIM2 Inflammasome Surveillance of DNA Damage Shapes Neurodevelopment,” Nature 580, no. 7805 (2020): 647–652. 10.1038/s41586-020-2174-3 PMC7788527 32350463

[9] J. Liang , Q. Wang , J. Q. Li , T. Guo , and D. Yu , “Long Non‐Coding RNA MEG3 Promotes Cerebral Ischemia‐Reperfusion Injury Through Increasing Pyroptosis By Targeting miR‐485/AIM2 Axis,” Experimental Neurology 325 (2020): 113139, 10.1016/j.expneurol.2019.113139. 31794744

[10] M. M. Hu and H. B. Shu , “Mitochondrial DNA‐Triggered Innate Immune Response: Mechanisms And Diseases,” Cellular & Molecular Immunology 20, no. 12 (2023): 1403–1412, 10.1038/s41423-023-01086-x. PMC10687031 37932533

[11] L.‐Q. Wang , T. Liu , S. Yang , et al., “Perfluoroalkyl Substance Pollutants Activate The Innate Immune System Through The AIM2 Inflammasome,” Nature Communications 12, no. 1 (2021): 2915, 10.1038/s41467-021-23201-0. PMC8131593 34006824

[12] S. Victorelli , H. Salmonowicz , J. Chapman , et al., “Apoptotic Stress Causes mtDNA Release During Senescence And Drives The SASP,” Nature 622, no. 7983 (2023): 627–636, 10.1038/s41586-023-06621-4. PMC10584674 37821702

[13] C.‐H. Yu , S. Davidson , C. R. Harapas , et al., “TDP‐43 Triggers Mitochondrial DNA Release via mPTP to Activate cGAS/STING in ALS,” Cell 183, no. 3 (2020): 636–649, 10.1016/j.cell.2020.09.020. PMC7599077 33031745

[14] J. S. Riley , G. Quarato , C. Cloix , et al., “Mitochondrial Inner Membrane Permeabilisation Enables mtDNA Release During Apoptosis,” The EMBO Journal 37, no. 17 (2018): 99238, 10.15252/embj.201899238. PMC6120664 30049712

[15] B. He , H. Yu , S. Liu , et al., “Mitochondrial Cristae Architecture Protects Against mtDNA Release And Inflammation,” Cell Reports 41, no. 10 (2022): 111774, 10.1016/j.celrep.2022.111774. 36476853

[16] V. Shoshan‐Barmatz , V. De Pinto , M. Zweckstetter , Z. Raviv , N. Keinan , and N. Arbel , “VDAC, A Multi‐Functional Mitochondrial Protein Regulating Cell Life And Death,” Molecular Aspects of Medicine 31, no. 3 (2010): 227–285, 10.1016/j.mam.2010.03.002. 20346371

[17] V. Shoshan‐Barmatz , E. N. Maldonado , and Y. Krelin , “VDAC1 at the Crossroads of Cell Metabolism,” Apoptosis and Cell Stress,″ Cell Stress 1, no. 1 (2017): 11–36, 10.15698/cst2017.10.104. PMC6287957 30542671

[18] V. Shoshan‐Barmatz , Y. Krelin , A. Shteinfer‐Kuzmine , and T. Arif , “Voltage‐Dependent Anion Channel 1 As an Emerging Drug Target for Novel Anti‐Cancer Therapeutics,” Frontiers in Oncology 7 (2017): 154, 10.3389/fonc.2017.00154. PMC5534932 28824871

[19] V. Shoshan‐Barmatz , A. Shteinfer‐Kuzmine , and A. Verma , “VDAC1 at the Intersection of Cell Metabolism, Apoptosis, and Diseases,” Biomolecules 10, no. 11 (2020): 1485, 10.3390/biom10111490. PMC7693975 33114780

[20] V. De Pinto , “Renaissance of VDAC: New Insights on a Protein Family at the Interface between Mitochondria and Cytosol,” Biomolecules 11, no. 1 (2021): 107, 10.3390/biom11010107. PMC7831034 33467485

[21] N. N. Wu , L. Wang , L. Wang , et al., “Site‐Specific Ubiquitination of VDAC1 Restricts Its Oligomerization And Mitochondrial DNA Release In Liver Fibrosis,” Experimental & Molecular Medicine 55, no. 1 (2023): 269–280, 10.1038/s12276-022-00924-w. PMC9898252 36658227

[22] F. Akhter , A. Akhter , X. Zhu , et al., “Amyloid‐Beta Glycation Induces Neuronal Mitochondrial Dysfunction And Alzheimer's Pathogenesis Via VDAC1‐Dependent mtDNA Efflux,” Proceedings of the National Academy of Sciences 122, no. 47 (2025): 2505046122, 10.1073/pnas.2505046122. PMC12664008 41248283

[23] J. Kim , R. Gupta , L. P. Blanco , et al., “VDAC Oligomers Form Mitochondrial Pores To Release mtDNA Fragments And Promote Lupus‐Like Disease,” Science 366, no. 6472 (2019): 1531–1536, 10.1126/science.aav4011. PMC8325171 31857488

[24] F.‐Q. Huang , H.‐F. Wang , T. Yang , et al., “Ceramides Increase Mitochondrial Permeabilization To Trigger mtDNA‐Dependent Inflammation In Astrocytes During Brain Ischemia,” Metabolism 166 (2025): 156161, 10.1016/j.metabol.2025.156161. 39956315

[25] N. Fenouille , C. F. Bassil , I. Ben‐Sahra , et al., “The Creatine Kinase Pathway Is A Metabolic Vulnerability In EVI1‐Positive Acute Myeloid Leukemia,” Nature Medicine 23, no. 3 (2017): 301–313, 10.1038/nm.4283. PMC5540325 28191887

[26] C. D. Rae and S. Bröer , “Creatine As A Booster For Human Brain Function. How Might It Work?,” Neurochemistry International 89 (2015): 249–259, 10.1016/j.neuint.2015.07.005. 26297632

[27] C. Datler , E. Pazarentzos , A.‐L. Mahul‐Mellier , et al., “CKMT1 Regulates The Mitochondrial Permeability Transition Pore In A Process That Provides Evidence For Alternative Forms Of The Complex,” Journal of Cell Science 127, no. 8 (2014): 1816–1828, 10.1242/jcs.140467. PMC3986677 24522192

[28] V. Ayyappan , N. M. Jenkinson , C. M. Tressler , et al., “Context‐Dependent Roles For Ubiquitous Mitochondrial Creatine Kinase CKMT1 In Breast Cancer Progression,” Cell Reports 43, no. 4 (2024): 114121, 10.1016/j.celrep.2024.114121. PMC11100297 38615320

[29] R. Lan , F. Huang , G. Zhong , et al., “Effects of CKMT1 on Radiosensitivity Of Nasopharyngeal Carcinoma Cells,” International Journal of Radiation Biology 95, no. 5 (2019): 597–606, 10.1080/09553002.2019.1566682. 30507333

[30] M. Li , H. Liu , J. Li , S. Guo , and Y. Lv , “Mitochondrial Creatine Kinase 1 In Non‐Small Cell Lung Cancer Progression And Hypoxia Adaptation,” Respiratory Research 22, no. 1 (2021): 190, 10.1186/s12931-021-01776-2. PMC8247087 34210337

[31] Z. Wang , H. Wu , X. Chang , et al., “CKMT1 Deficiency Contributes To Mitochondrial Dysfunction And Promotes Intestinal Epithelial Cell Apoptosis Via Reverse Electron Transfer‐Derived ROS in Colitis,” Cell Death & Disease 16, no. 1 (2025): 177, 10.1038/s41419-025-07410-5. PMC11910573 40089459

[32] S. Wang , Y. Song , Z. Wang , et al., “Neutrophil‐Derived PAD4 Induces Citrullination Of CKMT1 Exacerbates Mucosal Inflammation In Inflammatory Bowel Disease,” Cellular & Molecular Immunology 21, no. 6 (2024): 620–633, 10.1038/s41423-024-01160-8. PMC11143373 38720063

[33] D. Ben‐Hail , R. Begas‐Shvartz , M. Shalev , et al., “Novel Compounds Targeting the Mitochondrial Protein VDAC1 Inhibit Apoptosis and Protect against Mitochondrial Dysfunction,” Journal of Biological Chemistry 291, no. 48 (2016): 24986–25003, 10.1074/jbc.M116.744284. PMC5122769 27738100

[34] M. Santhanam , V. Babu , A. Shteinfer‐Kuzmine , R. Zalk , and V. Shoshan‐Barmaz , “VDAC1‐Interacting Proteins: Binding Site Mapping and Their Derived Peptides Induce Apoptosis and Multifaceted Cellular Effects,” Apoptosis 30, no. 11–12 (2025): 2938–2959, 10.1007/s10495-025-02185-y. PMC12669278 41006687

[35] H. Zhou , Z. Dai , J. Li , et al., “TMBIM6 Prevents VDAC1 Multimerization And Improves Mitochondrial Quality Control To Reduce Sepsis‐Related Myocardial Injury,” Metabolism 140 (2023): 155383, 10.1016/j.metabol.2023.155383. 36603706

[36] J. Yang , X. Lu , J.‐L. Hao , et al., “VSTM2L Protects Prostate Cancer Cells Against Ferroptosis Via Inhibiting VDAC1 Oligomerization And Maintaining Mitochondria Homeostasis,” Nature Communications 16, no. 1 (2025): 1160, 10.1038/s41467-025-56539-4. PMC11779845 39880844

[37] H. Wan , Y. Yan , X. Hu , et al., “Inhibition of Mitochondrial VDAC1 Oligomerization Alleviates Apoptosis and Necroptosis of Retinal Neurons Following OGD/R Injury,” Annals of Anatomy – Anatomischer Anzeiger 247 (2023): 152049, 10.1016/j.aanat.2023.152049. 36690044

[38] H. Wan , Y. Yang , Q. Zhang , et al., “VDAC1, as a Downstream Molecule of MLKL, Participates in OGD/R‐Induced Necroptosis by Inducing Mitochondrial Damage,” Heliyon 10, no. 1 (2024): e23426, 10.1016/j.heliyon.2023.e23426. PMC10761567 38173512

[39] S. He , T. Pan , R. Tian , et al., “Fatty Acid Synthesis Promotes mtDNA Release via ETS1‐Mediated Oligomerization Of VDAC1 Facilitating Endothelial Dysfunction In Sepsis‐Induced Lung Injury,” Cell Death & Differentiation 32, no. 12 (2025): 2177–2192, 10.1038/s41418-025-01426-8. PMC12669790 40369168

[40] V. Paschon , B. C. Morena , F. F. Correia , et al., “VDAC1 Is Essential For Neurite Maintenance And The Inhibition Of Its Oligomerization Protects Spinal Cord From Demyelination And Facilitates Locomotor Function Recovery After Spinal Cord Injury,” Scientific Reports 9, no. 1 (2019): 14063, 10.1038/s41598-019-50506-4. PMC6773716 31575916

[41] B. Niu , X. Lei , Q. Xu , et al., “Protecting Mitochondria Via Inhibiting VDAC1 Oligomerization Alleviates Ferroptosis In Acetaminophen‐Induced Acute Liver Injury,” Cell Biology and Toxicology 38, no. 3 (2022): 505–530, 10.1007/s10565-021-09623-6. 34401974

[42] A. Verma , A. Shteinfer‐Kuzmine , N. Kamenetsky , et al., “Targeting The Overexpressed Mitochondrial Protein VDAC1 In A Mouse Model Of Alzheimer's Disease Protects Against Mitochondrial Dysfunction And Mitigates Brain Pathology,” Translational Neurodegeneration 11, no. 1 (2022): 58, 10.1186/s40035-022-00328-y. PMC9795455 36578022

[43] X. Zhou , X. M. Tang , T. Li , et al., “Inhibition of VDAC1 Rescues Aβ(1‐42)‐Induced Mitochondrial Dysfunction and Ferroptosis via Activation of AMPK and Wnt/β‐Catenin Pathways,” Mediators of Inflammation 2023 (2023): 6739691, 10.1155/2023/6739691. PMC9937775 36816741

[44] X. Luo and J. Yue , “VDAC1 Inhibition Mitigates Inflammatory Status and Oxidative Stress in Epileptic Mice Treated with the Ketogenic Diet,” Neurochemical Research 50, no. 2 (2025): 118, 10.1007/s11064-024-03840-5. 40085179

[45] W. Jiang , B. Du , Z. Chi , et al., “Preliminary explorations of the role of mitochondrial proteins in refractory epilepsy: Some Findings From Comparative Proteomics,” Journal of Neuroscience Research 85, no. 14 (2007): 3160–3170, 10.1002/jnr.21466. 17893921

[46] G.‐Y. Yao , Q. Zhu , J. Xia , et al., “Ischemic Postconditioning Confers Cerebroprotection By Stabilizing VDACs After Brain Ischemia,” Cell Death & Disease 9, no. 10 (2018): 1033, 10.1038/s41419-018-1089-5. PMC6180002 30305621

[47] P. L. F. Costa , M. M. França , M. L. Katayama , et al., “Circulating Cell‐Free mtDNA Contributes to AIM2 Inflammasome‐Mediated Chronic Inflammation in Patients with Type 2 Diabetes,” Cells 8, no. 4 (2019): 318, 10.3390/cells8040318. PMC6524162 30965677

[48] L. Giordano , S. A. Ware , C. J. Lagranha , and B. A. Kaufman , “Mitochondrial DNA Signals Driving Immune Responses: Why, How, Where?,” Cell Communication and Signaling 23, no. 1 (2025): 192, 10.1186/s12964-025-02106-5. PMC12012978 40264103

[49] L. Xu , J. Zhou , J. Che , et al., “Mitochondrial DNA Enables AIM2 Inflammasome Activation And Hepatocyte Pyroptosis In Nonalcoholic Fatty Liver Disease,” American Journal of Physiology‐Gastrointestinal and Liver Physiology 320, no. 6 (2021): G1034–G1044, 10.1152/ajpgi.00431.2020. 33728991

[50] A. Denes , G. Coutts , N. Lénárt , et al., “AIM2 and NLRC4 Inflammasomes Contribute With ASC To Acute Brain Injury Independently of NLRP3,” Proceedings of the National Academy of Sciences 112, no. 13 (2015): 4050–4055, 10.1073/pnas.1419090112. PMC4386342 25775556

[51] P. Habib , J. Harms , A. Zendedel , C. Beyer , and A. Slowik , “Gonadal Hormones E2 and P Mitigate Cerebral Ischemia‐Induced Upregulation of the AIM2 and NLRC4 Inflammasomes in Rats,” International Journal of Molecular Sciences 21, no. 13 (2020): 4795, 10.3390/ijms21134795. PMC7370209 32645874

[52] H. Kim , J. S. Seo , S.‐Y. Lee , et al., “AIM2 Inflammasome Contributes To Brain Injury And Chronic Post‐Stroke Cognitive Impairment In Mice,” Brain, Behavior, and Immunity 87 (2020): 765–776, 10.1016/j.bbi.2020.03.011. 32201254

[53] G. You , L. Zheng , Y. Zhang , et al., “Tangeretin Attenuates Cerebral Ischemia–Reperfusion‐Induced Neuronal Pyroptosis by Inhibiting AIM2 Inflammasome Activation via Regulating NRF2,” Inflammation 47, no. 1 (2024): 145–158, 10.1007/s10753-023-01906-8. 37725272

[54] Q. Li , L. Yang , K. Wang , et al., “Oxidized Mitochondrial DNA Activates The cGAS‐STING Pathway In The Neuronal Intrinsic Immune System After Brain Ischemia‐Reperfusion Injury,” Neurotherapeutics 21, no. 4 (2024): e00368, 10.1016/j.neurot.2024.e00368. PMC11284550 38688786

[55] H. Liu , H. Fan , P. He , et al., “Prohibitin 1 Regulates mtDNA Release And Downstream Inflammatory Responses,” The EMBO Journal 41, no. 24 (2022): 111173, 10.15252/embj.2022111173. PMC9753472 36245295

[56] M. Dolder , S. Wendt , and T. Wallimann , “Mitochondrial Creatine Kinase in Contact Sites: Interaction with Porin and Adenine Nucleotide Translocase, Role in Permeability Transition and Sensitivity to Oxidative Damage,” Neuro‐Signals 10, no. 1–2 (2001): 93–111, 10.1159/000046878. 11223643

[57] S. Geula , H. Naveed , J. Liang , and V. Shoshan‐Barmatz , “Structure‐based Analysis of VDAC1 Protein,” Journal of Biological Chemistry 287, no. 3 (2012): 2179–2190, 10.1074/jbc.M111.268920. PMC3265896 22117062

[58] H. Li , S. Sun , W. Guo , et al., “Positively Charged Cytoplasmic Residues In Corin Prevent Signal Peptidase Cleavage And Endoplasmic Reticulum Retention,” Communications Biology 8, no. 1 (2025): 89, 10.1038/s42003-025-07402-5. PMC11756421 39833422

[59] M. S. Azam and D. Missiakas , “The Signal Peptide Of Staphylococcal Protein A Alters The Multimeric States of PepV,” Microbiology Spectrum 13, no. 12 (2025): 0177825, 10.1128/spectrum.01778-25. PMC12671168 41143410

[60] M. Bayrhuber , T. Meins , M. Habeck , et al., “Structure Of The Human Voltage‐Dependent Anion Channel,” Proceedings of the National Academy of Sciences 105, no. 40 (2008): 15370–15375, 10.1073/pnas.0808115105. PMC2557026 18832158

[61] J. Preto and I. Krimm , “The Intrinsically Disordered N‐Terminus Of The Voltage‐Dependent Anion Channel,” PLOS Computational Biology 17, no. 2 (2021): 1008750, 10.1371/journal.pcbi.1008750. PMC7906469 33577583

[62] S. Abu‐Hamad , N. Arbel , D. Calo , et al., “The VDAC1 N‐Terminus Is Essential Both For Apoptosis And The Protective Effect Of Anti‐Apoptotic Proteins,” Journal of Cell Science 122, no. 11 (2009): 1906–1916, 10.1242/jcs.044768. 19461077

[63] N. Keinan , D. Tyomkin , and V. Shoshan‐Barmatz , “Oligomerization of the Mitochondrial Protein Voltage‐Dependent Anion Channel Is Coupled to the Induction of Apoptosis,” Molecular and Cellular Biology 30, no. 24 (2010): 5698–5709, 10.1128/MCB.00638-10. PMC3004265 20937774

[64] H. Justić , A. Barić , M. Radmilović , et al., “Redefining the Koizumi Model of Mouse Ischemic Stroke: A Comparative Longitudinal Study of Cerebral and Retinal Ischemia in the Koizumi and Longa Middle Cerebral Artery Occlusion Models,” Journal of Cerebral Blood Flow and Metabolism 42, no. 11 (2022): 2080–2094, 10.1177/0271678x221109507. PMC9580169 35748043

[65] P.‐F. Wang , H. Fang , J. Chen , et al., “Polyinosinic‐Polycytidylic Acid Has Therapeutic Effects against Cerebral Ischemia/Reperfusion Injury through the Downregulation of TLR4 Signaling via TLR3,” The Journal of Immunology 192, no. 10 (2014): 4783–4794, 10.4049/jimmunol.1303108. PMC4009499 24729619

[66] N. Pilati , M. Barker , S. Panteleimonitis , R. Donga , and M. Hamann , “A Rapid Method Combining Golgi and Nissl Staining to Study Neuronal Morphology and Cytoarchitecture,” Journal of Histochemistry & Cytochemistry 56, no. 6 (2008): 539–550, 10.1369/jhc.2008.950246. PMC2386766 18285350

[67] L.‐Y. Zhang , Y.‐Y. Hu , C.‐C. Zhao , et al., “The Mechanism Of GLT‐1 Mediating Cerebral Ischemic Injury Depends On The Activation Of p38 MAPK,” Brain Research Bulletin 147 (2019): 1–13, 10.1016/j.brainresbull.2019.01.017. 30731111

[68] S. H. Baik , V. K. Ramanujan , C. Becker , S. Fett , D. M. Underhill , and A. J. Wolf , “Hexokinase Dissociation From Mitochondria Promotes Oligomerization of VDAC That Facilitates NLRP3 Inflammasome Assembly And Activation,” Science Immunology 8, no. 84 (2023): ade7652, 10.1126/sciimmunol.ade7652. PMC10360408 37327321

[69] H. Hagihara , H. Shoji , H. Otabi , et al., “Protein Lactylation Induced By Neural Excitation,” Cell Reports 37, no. 2 (2021): 109820, 10.1016/j.celrep.2021.109820. 34644564

[70] H. Schägger and G. von Jagow , “Blue Native Electrophoresis For Isolation Of Membrane Protein Complexes In Enzymatically Active Form,” Analytical Biochemistry 199, no. 2 (1991): 223–231, 10.1016/0003-2697(91)90094-a. 1812789

[71] W. R. He , L. B. Cao , Y. L. Yang , D. Hua , M. M. Hu , and H. B. Shu , “VRK2 Is Involved In The Innate Antiviral Response By Promoting Mitostress‐Induced mtDNA Release,” Cellular & Molecular Immunology 18, no. 5 (2021): 1186–1196, 10.1038/s41423-021-00673-0. PMC8093274 33785841

[72] K.‐Y. Chu , A. N. Crawford , B. S. Krah , et al., “Cripto‐1 Acts As A Molecular Bridge Linking Nodal To ALK4 Via Distinct Structural Domains,” Protein Science 34, no. 2 (2025): 70034, 10.1002/pro.70034. PMC11751877 39840816

[73] F. Homma , J. Huang , and R. A. L. van der Hoorn , “Alphafold‐Multimer Predicts Cross‐Kingdom Interactions At The Plant‐Pathogen Interface,” Nature Communications 14, no. 1 (2023): 6040, 10.1038/s41467-023-41763-9. PMC10533508 37758696

[74] J. Sun , Y. Huang , J. Gong , et al., “Transplantation of hPSC‐Derived Pericyte‐Like Cells Promotes Functional Recovery In Ischemic Stroke Mice,” Nature Communications 11, no. 1 (2020): 5196, 10.1038/s41467-020-19020-0. PMC7566513 33060592

[75] J. Ma , T. Chen , S. Wu , et al., “iProX: An Integrated Proteome Resource,” Nucleic Acids Research 47, no. D1 (2019): D1211–D1217, 10.1093/nar/gky869. PMC6323926 30252093

[76] T. Chen , J. Ma , Y. Liu , et al., “iProX in 2021: Connecting Proteomics Data Sharing With Big Data,” Nucleic Acids Research 50, no. D1 (2022): D1522–D1527, 10.1093/nar/gkab935. PMC8728291 34871441

