# Supplementary material for: CKMT1 Counteracts VDAC1 Oligomerization‐Induced Neuronal Pyroptosis Through Steric Hindrance in Ischemic Stroke
Source: Adv Sci (Weinh). 2026 Nov 5:e78611. Online ahead of print. doi: 10.1002/advs.78611 (PMC13735431; doi:10.1002/advs.78611)

Figure1D

Nissl staining

Sham：

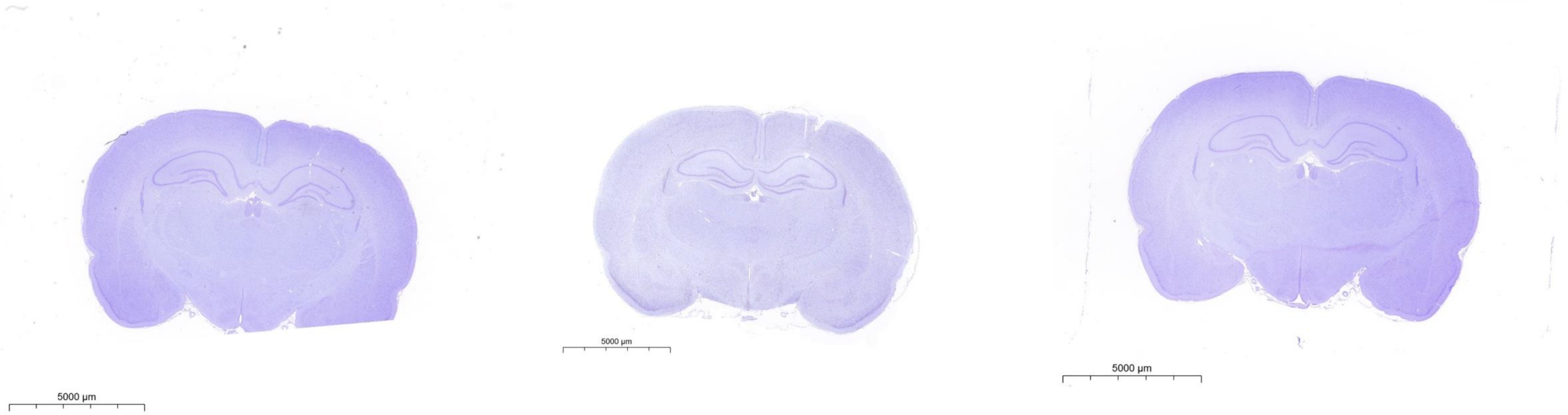

MCAO：

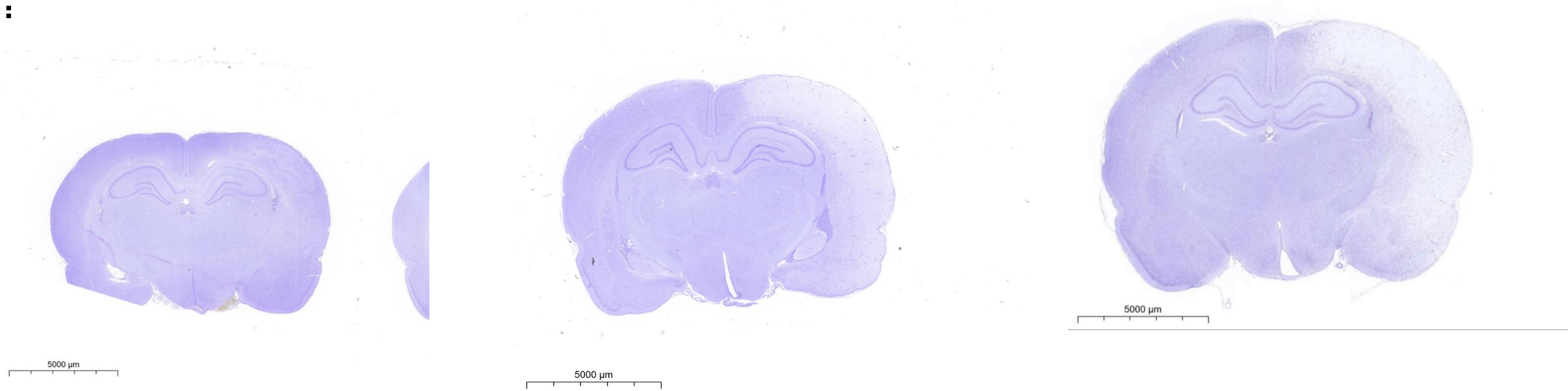

Figure1E

Sham:

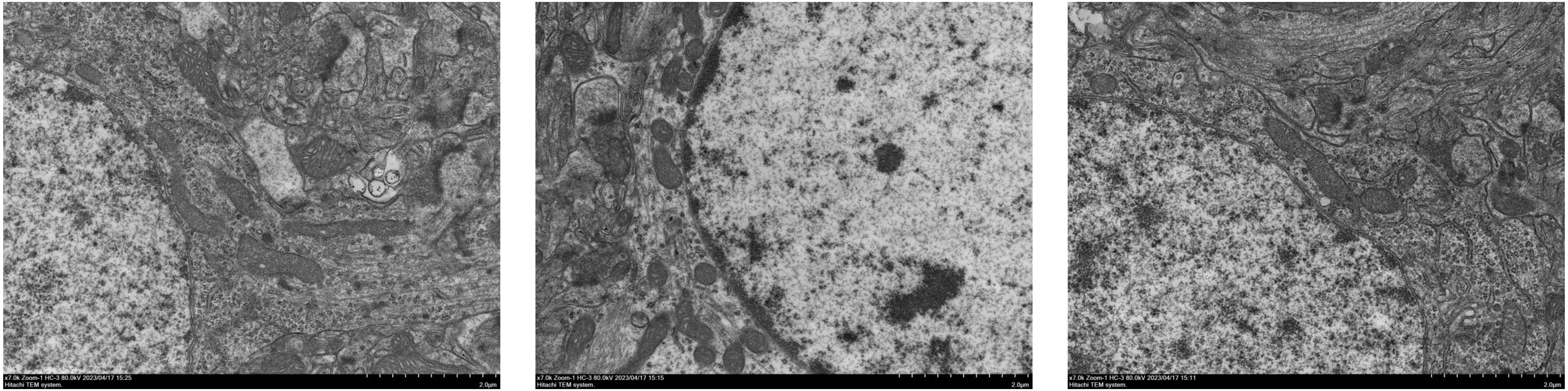

MCAO:

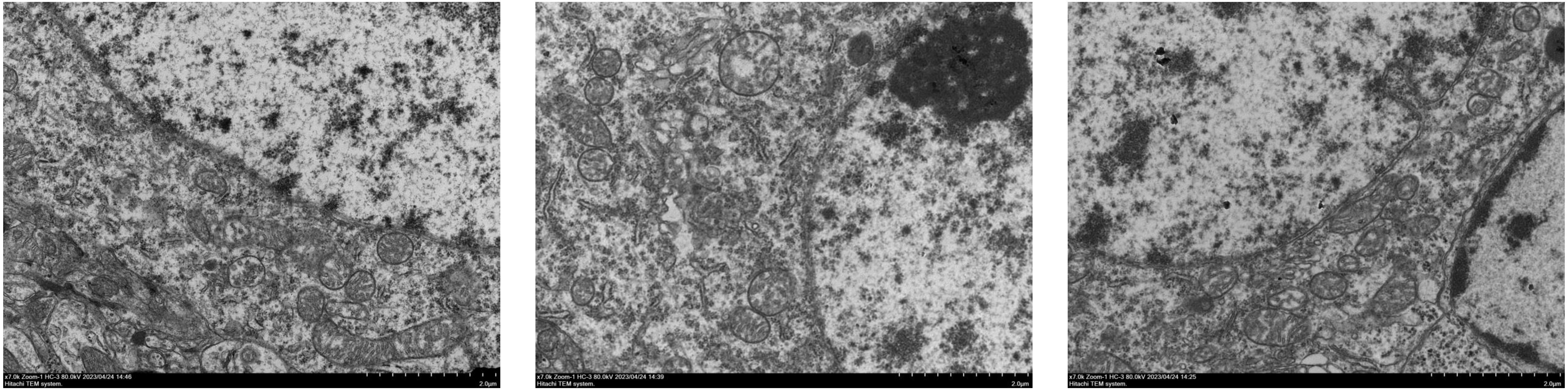

Figure1K

Control:

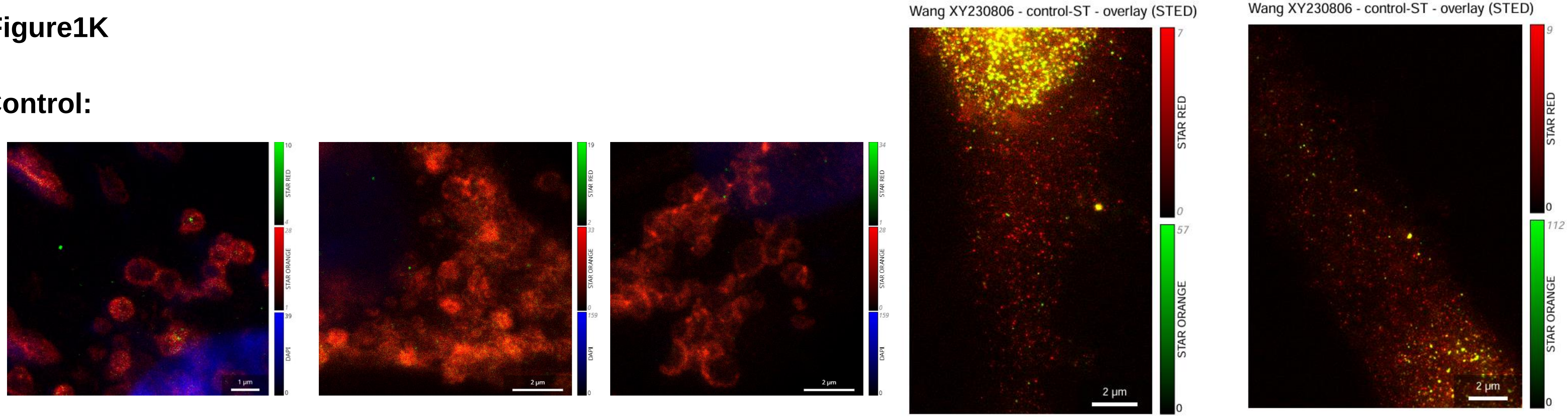

OGD:

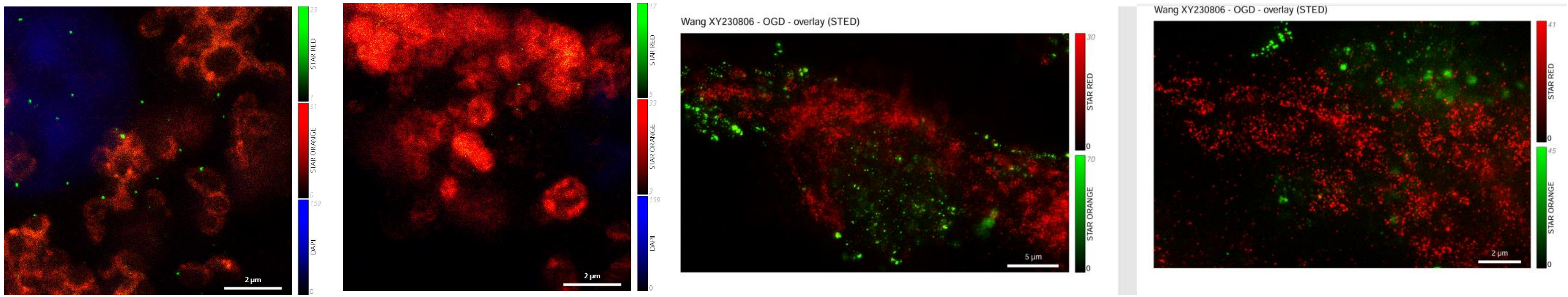

Figure1M

Vehicle+OGD:

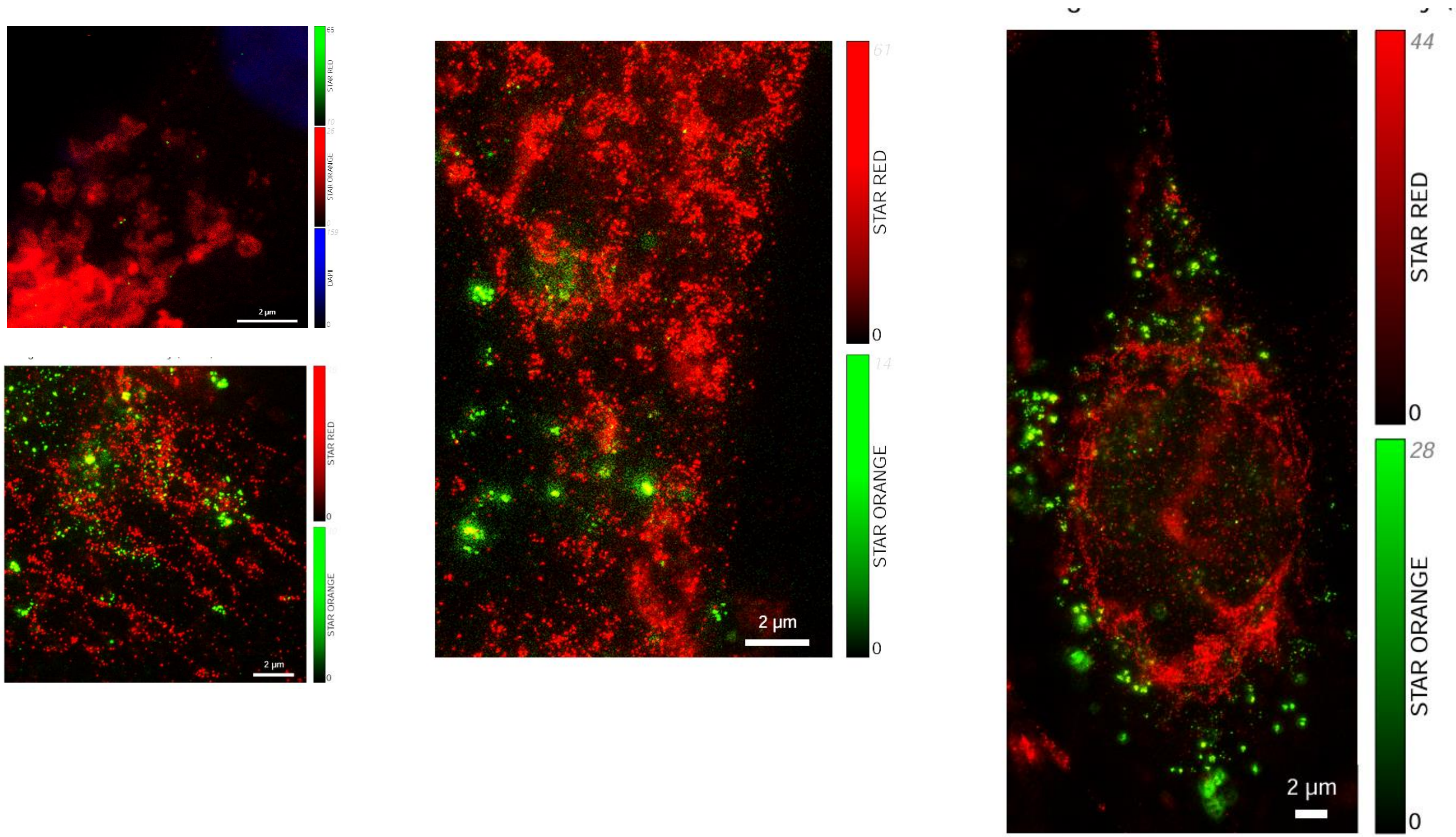

Figure1M

VBIT-4+OGD:

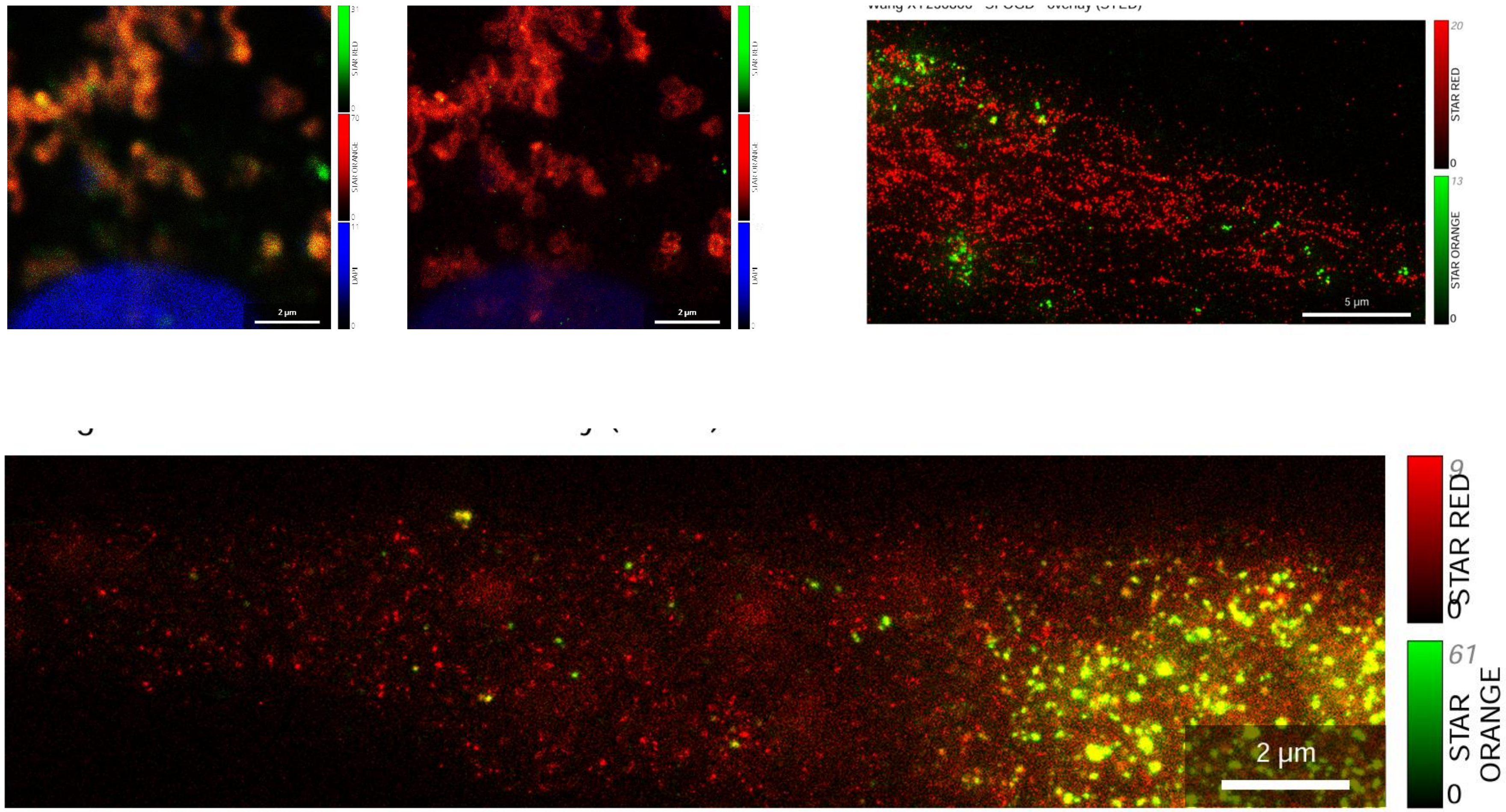

Figure2F

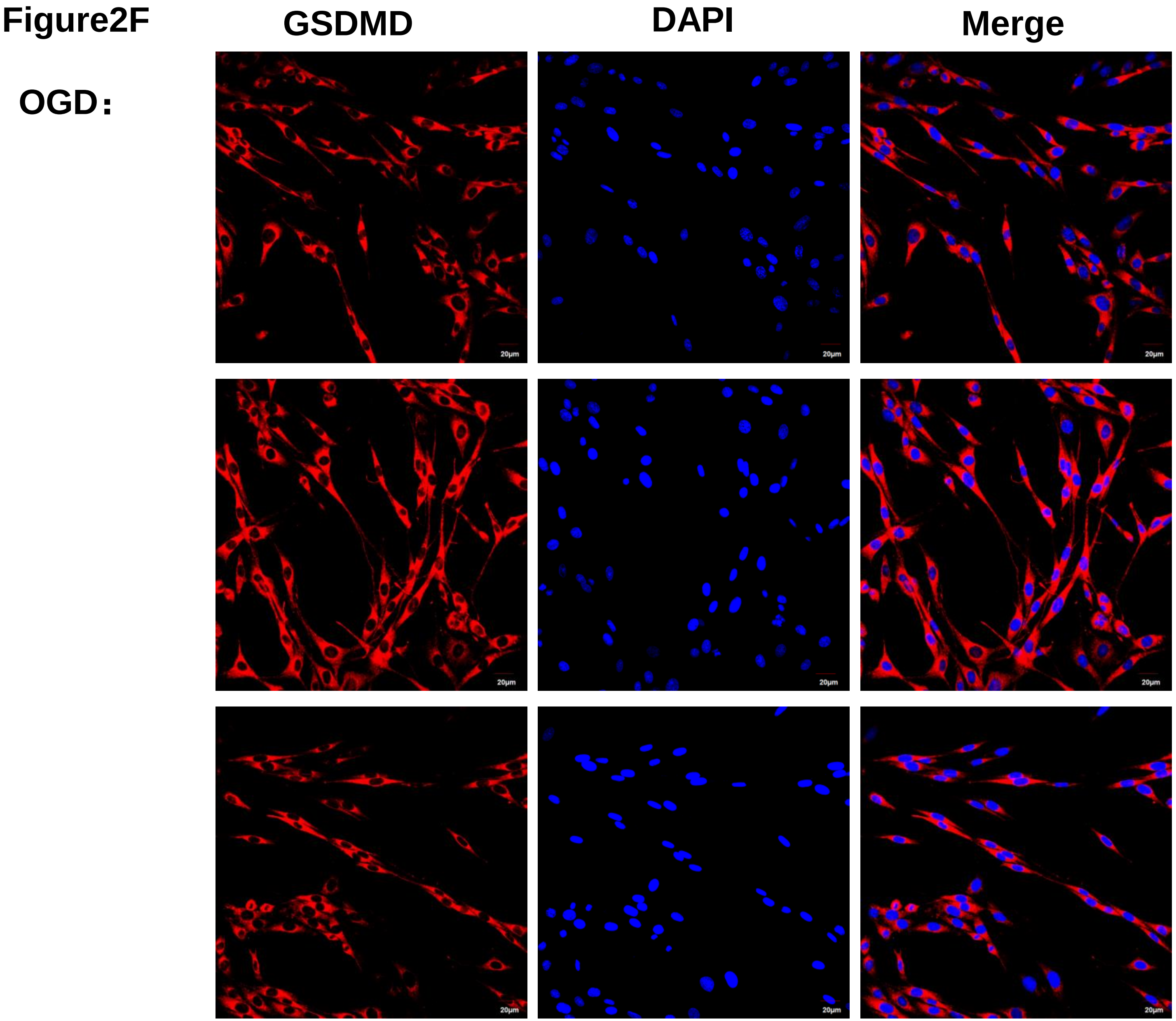

Figure2F

Vehicle+OGD：

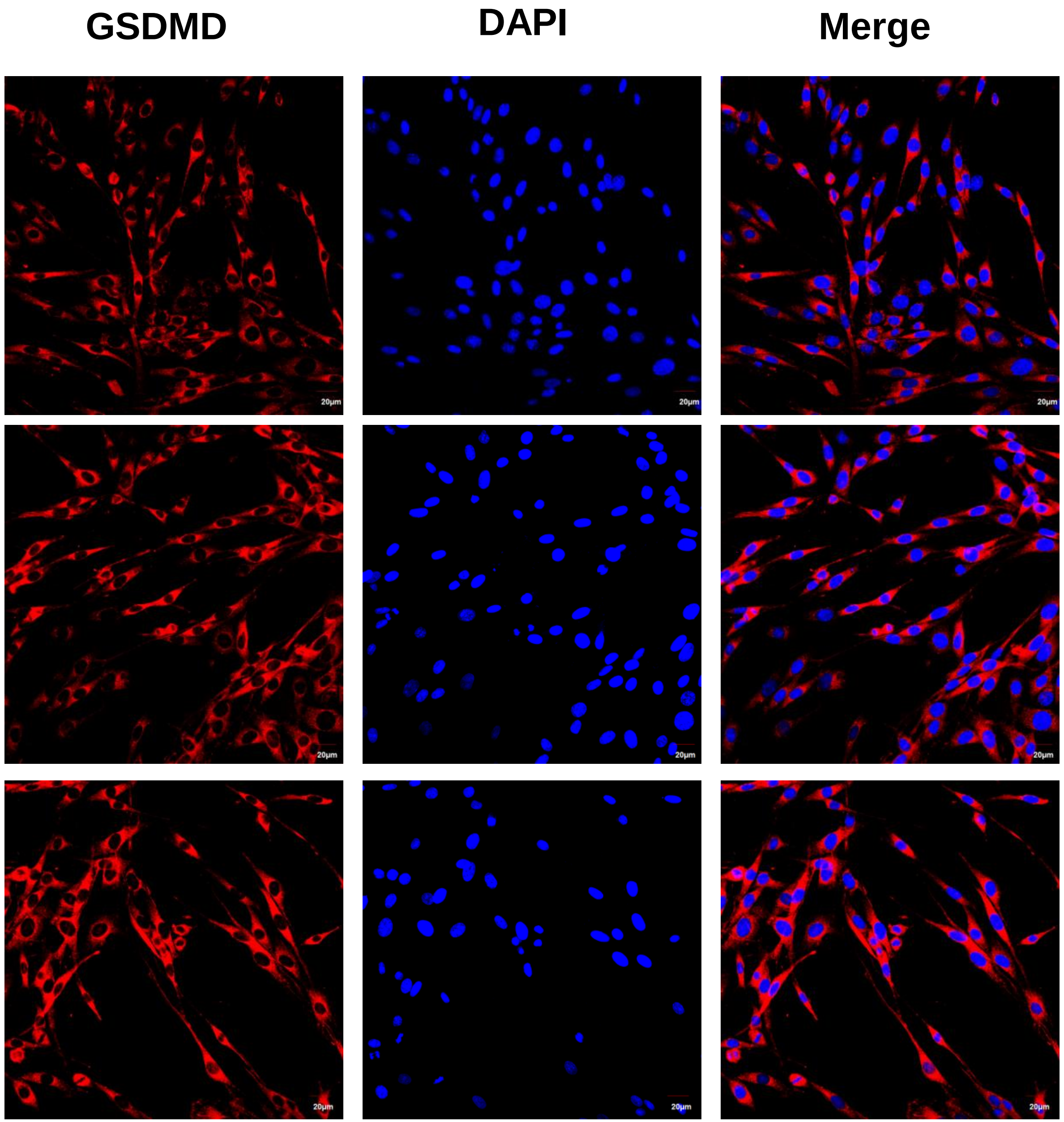

Figure2F

EtBr+OGD：

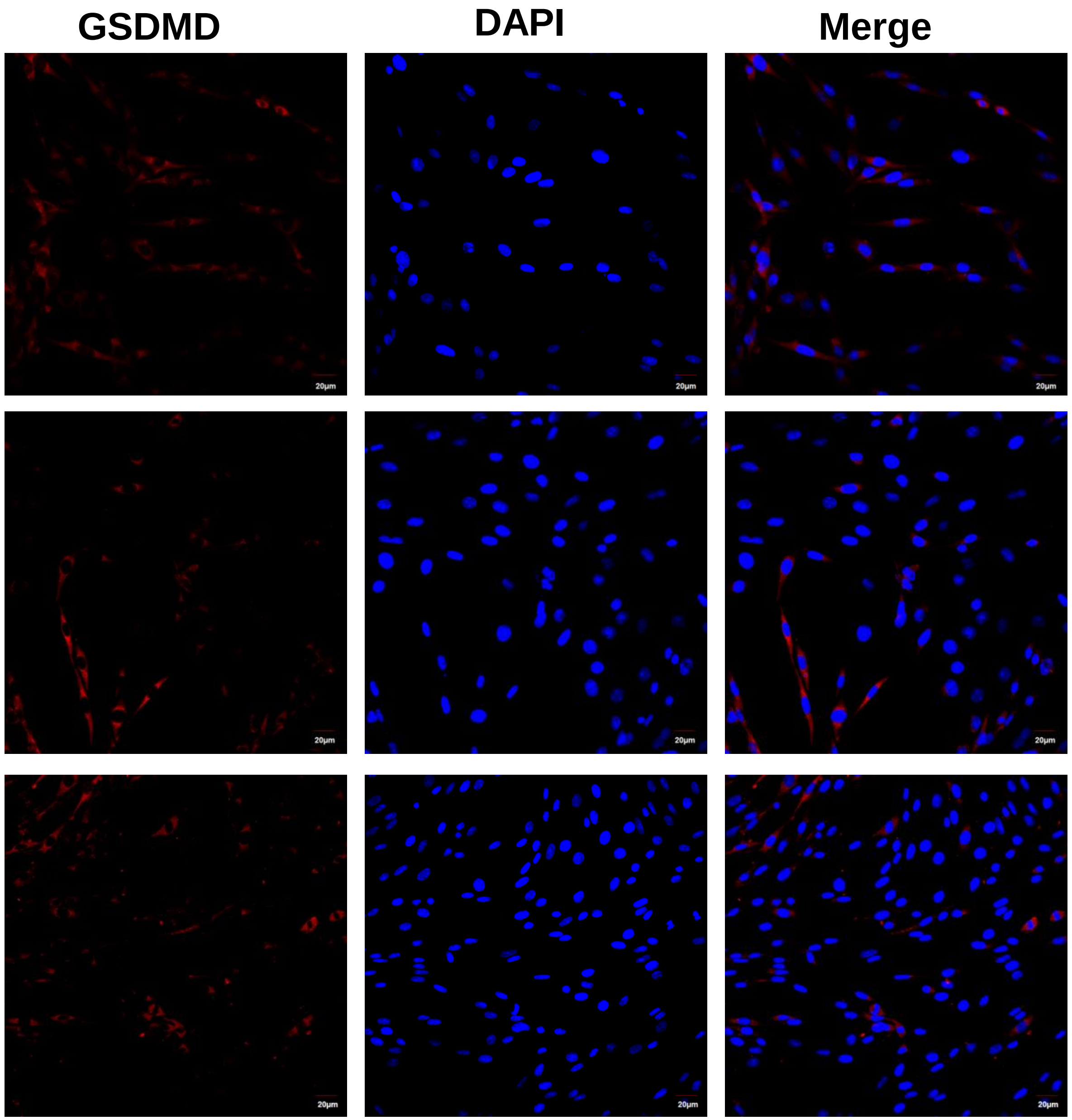

Figure2I

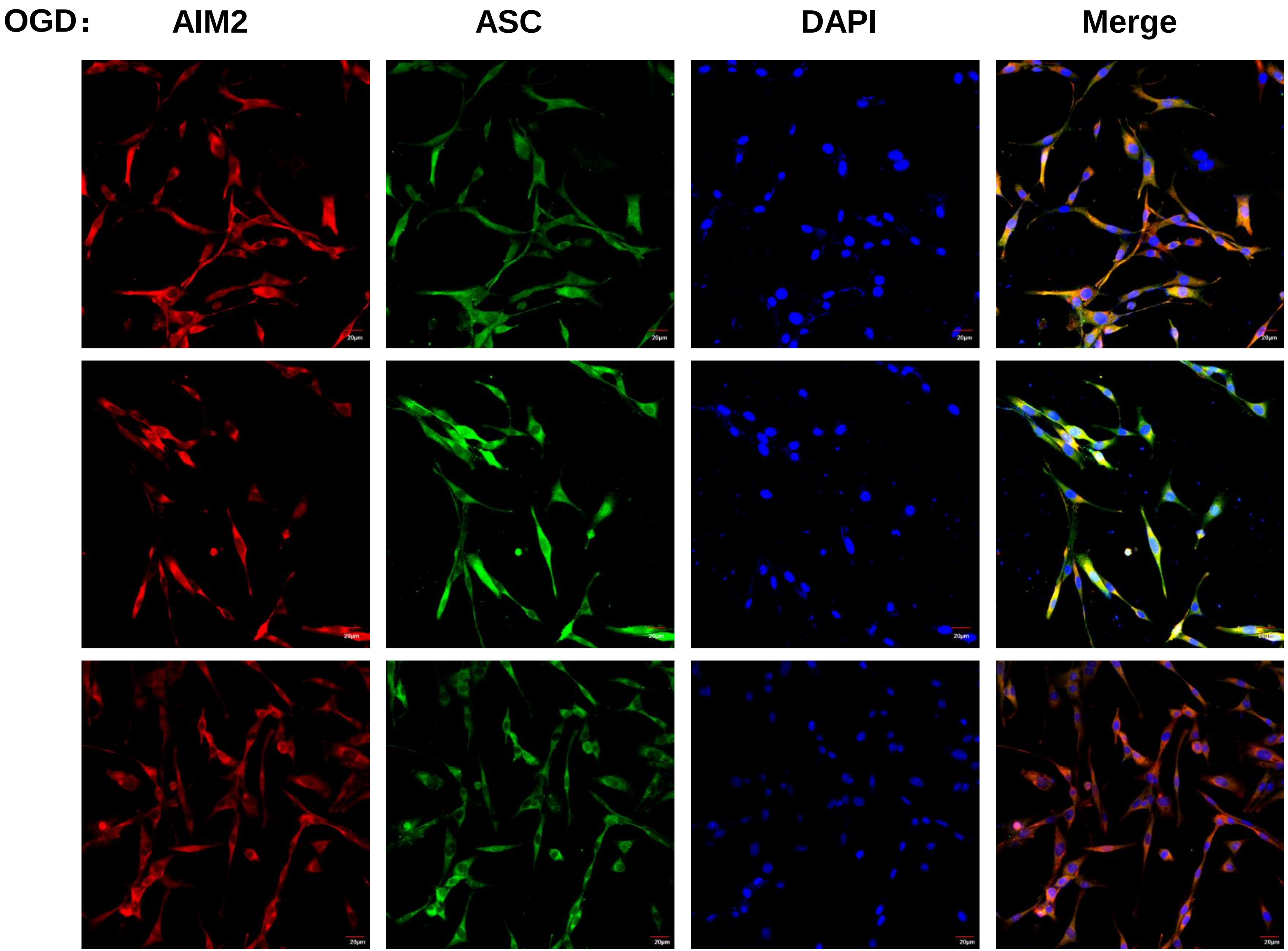

Figure2I

Vehicle+OGD：

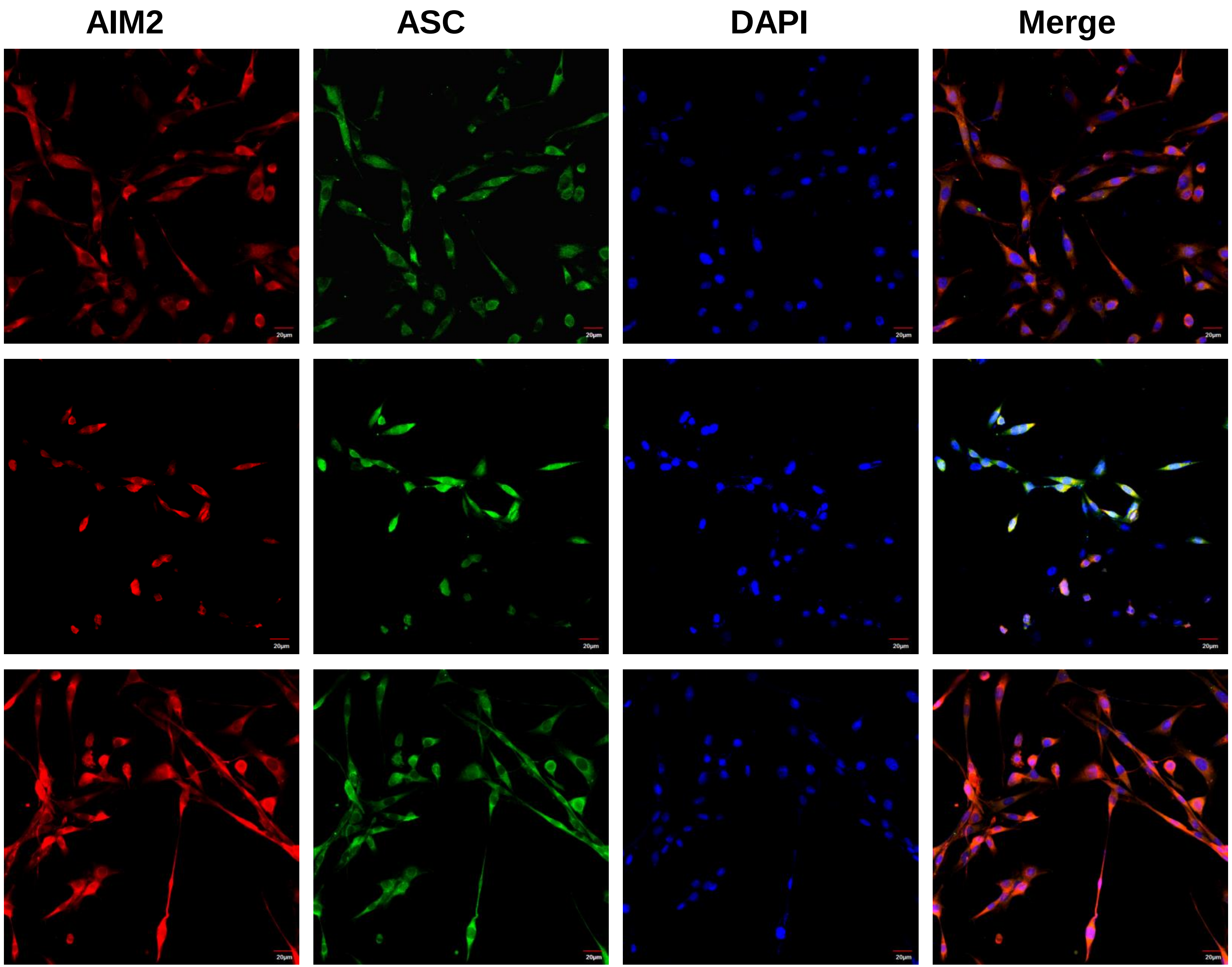

Figure2I

EtBr+OGD：

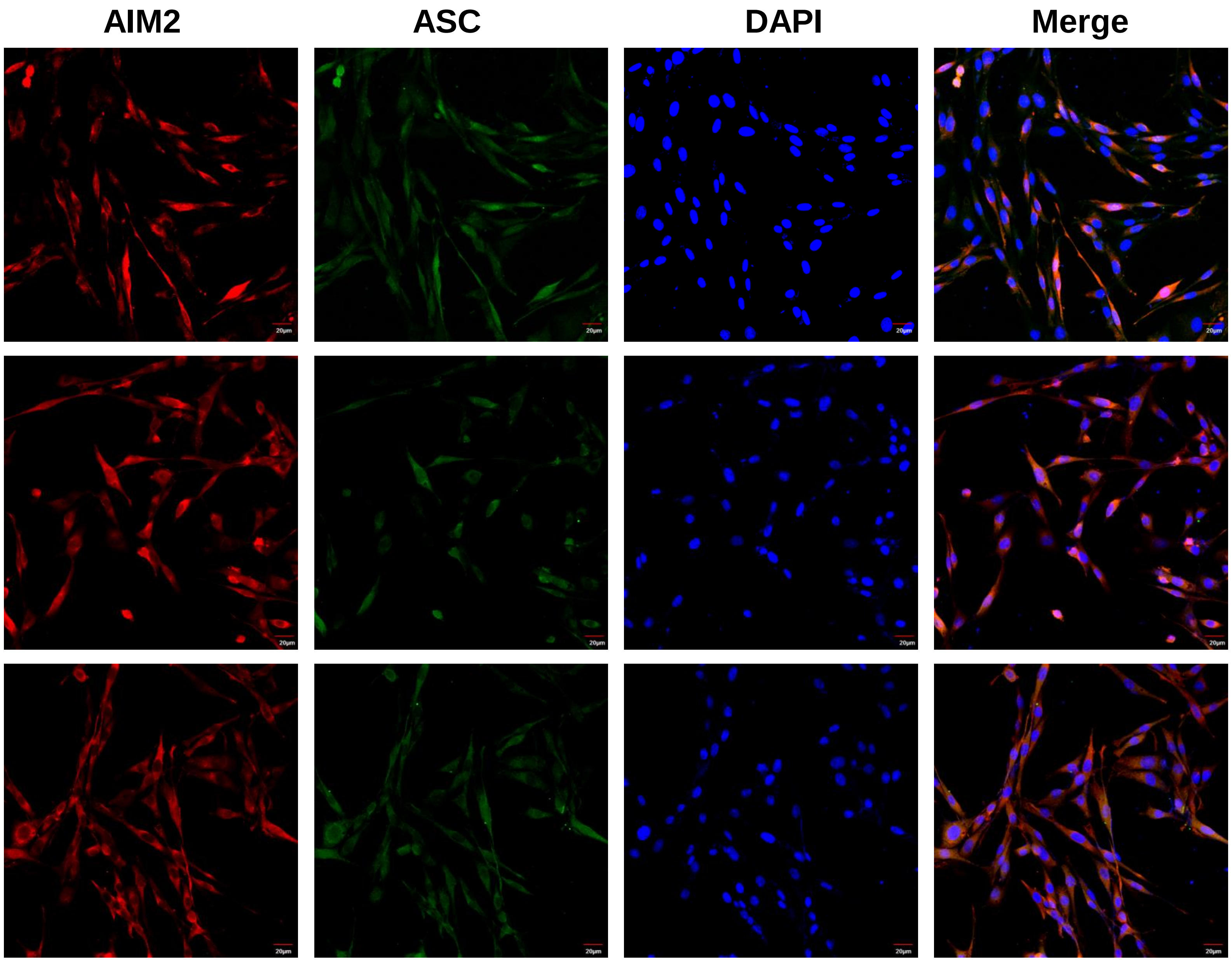

Figure3I

MCAO:

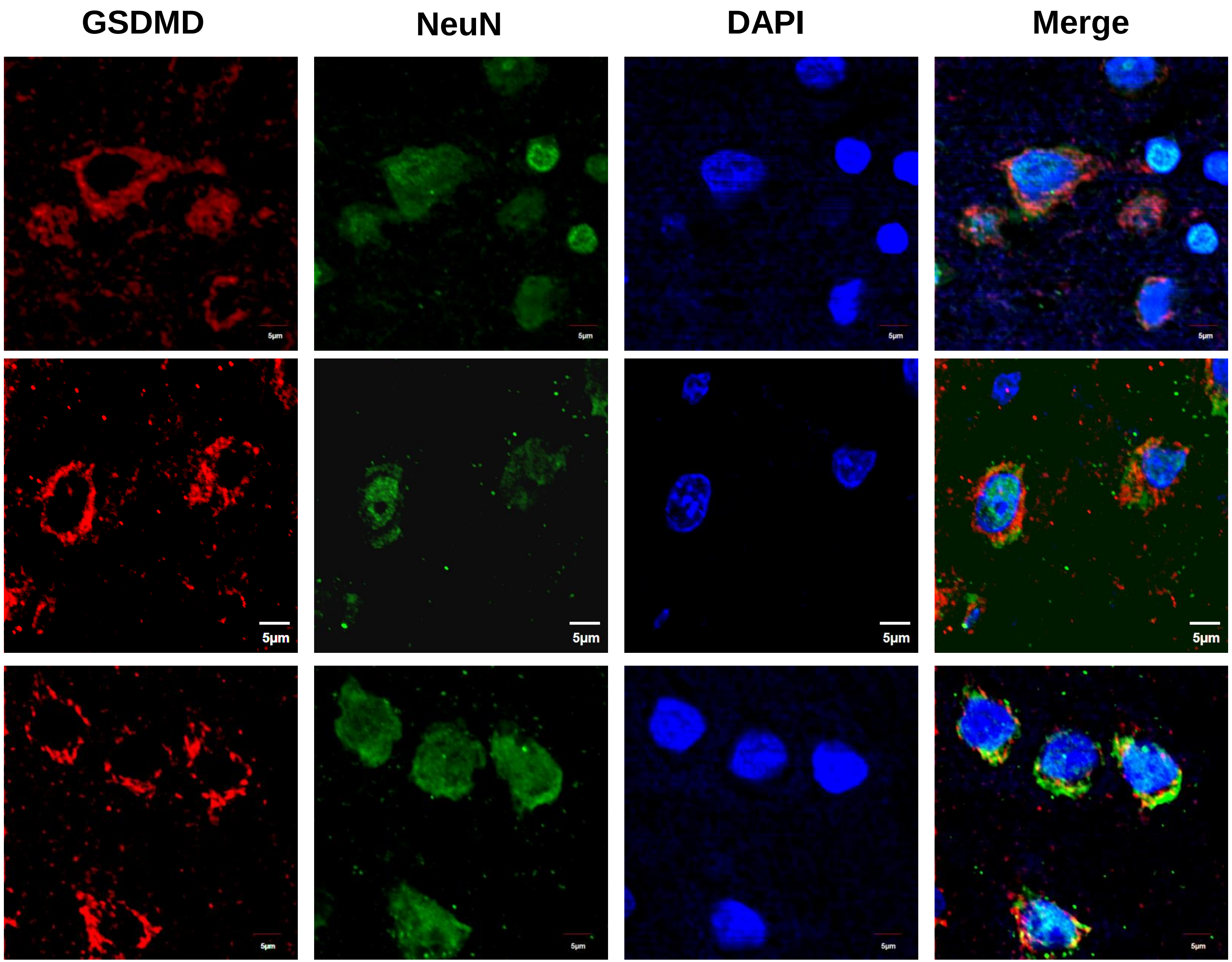

Figure3I

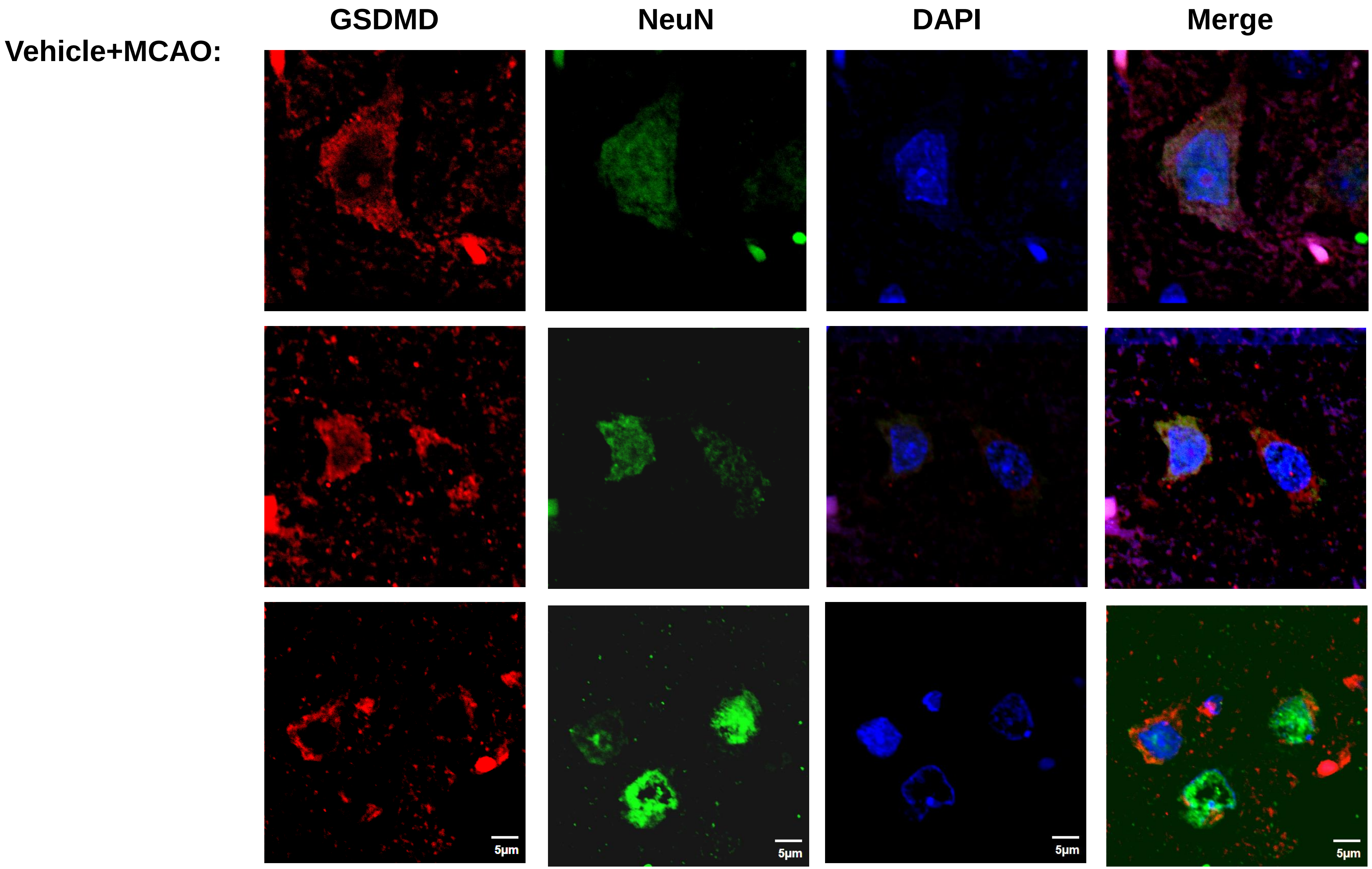

Figure3I

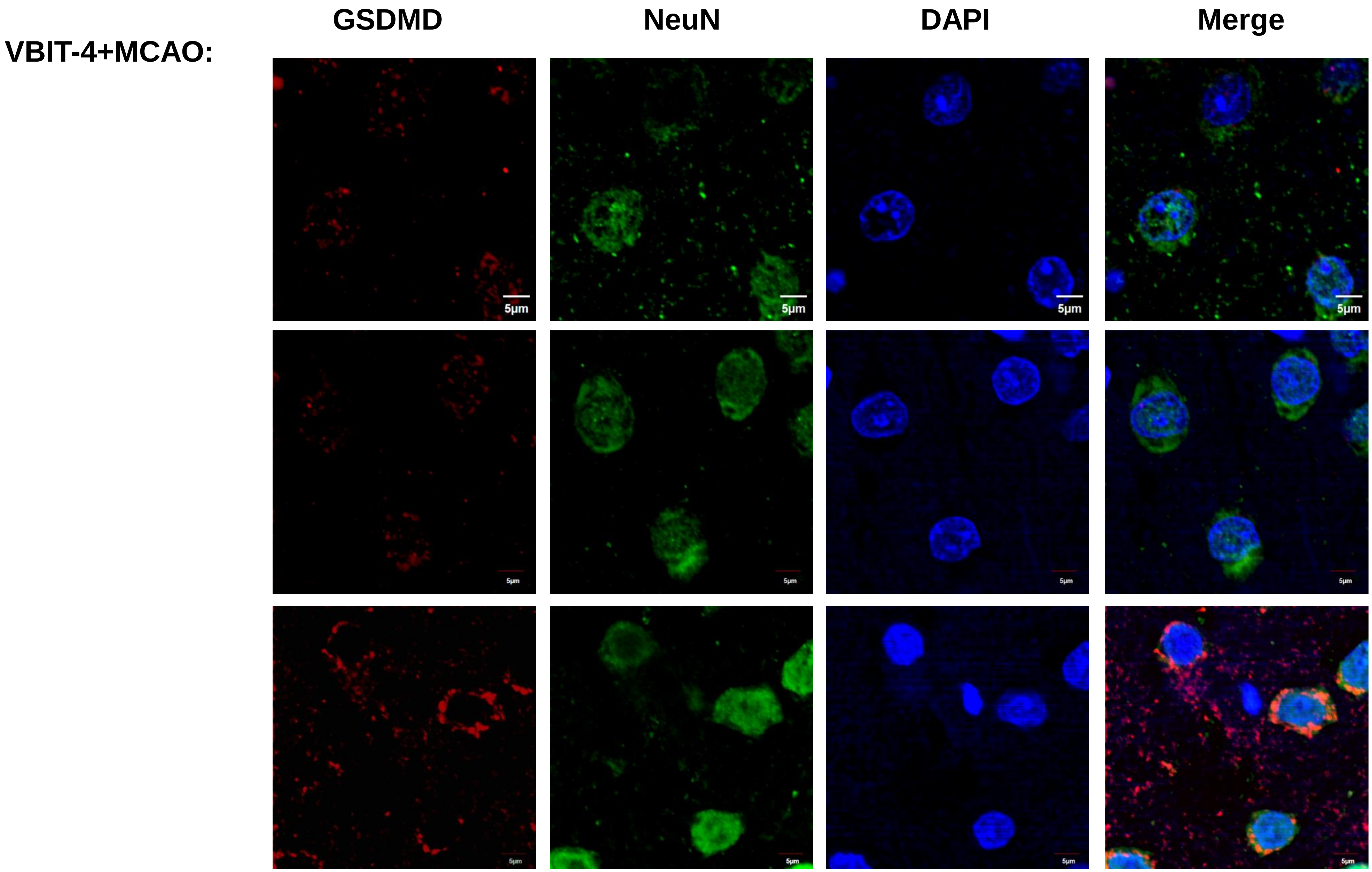

Figure3H

MCAO:

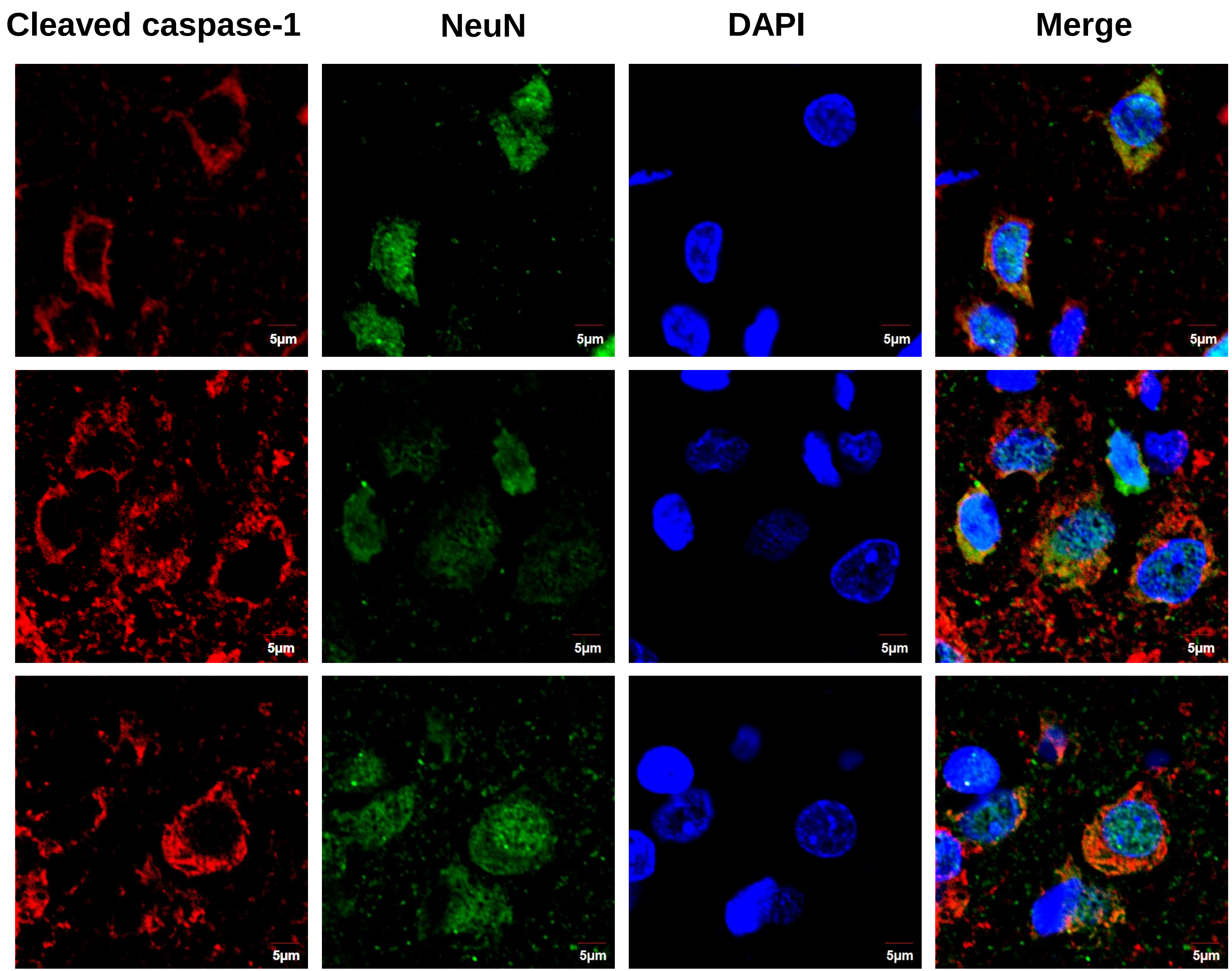

Figure3H

Vehicle+MCAO:

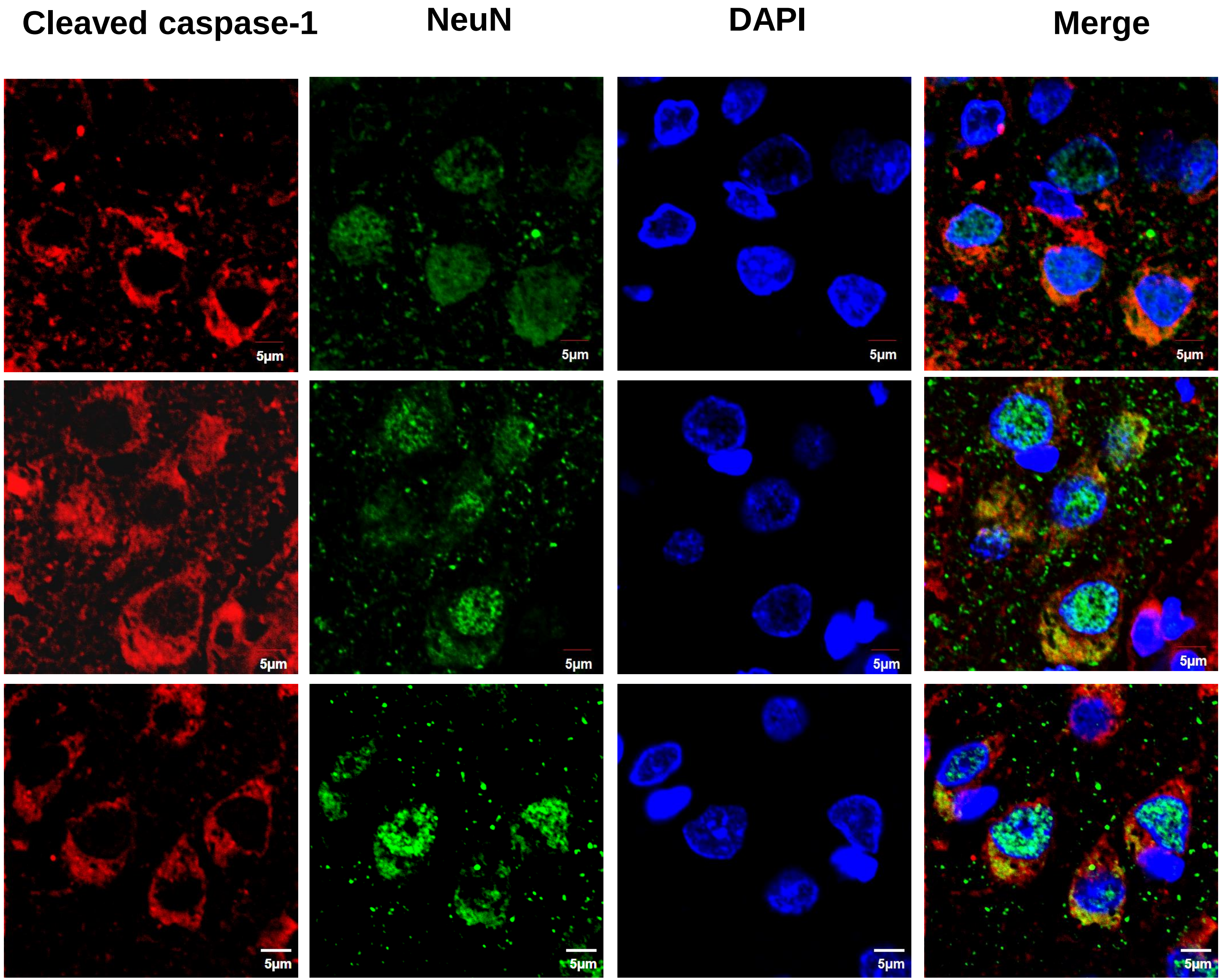

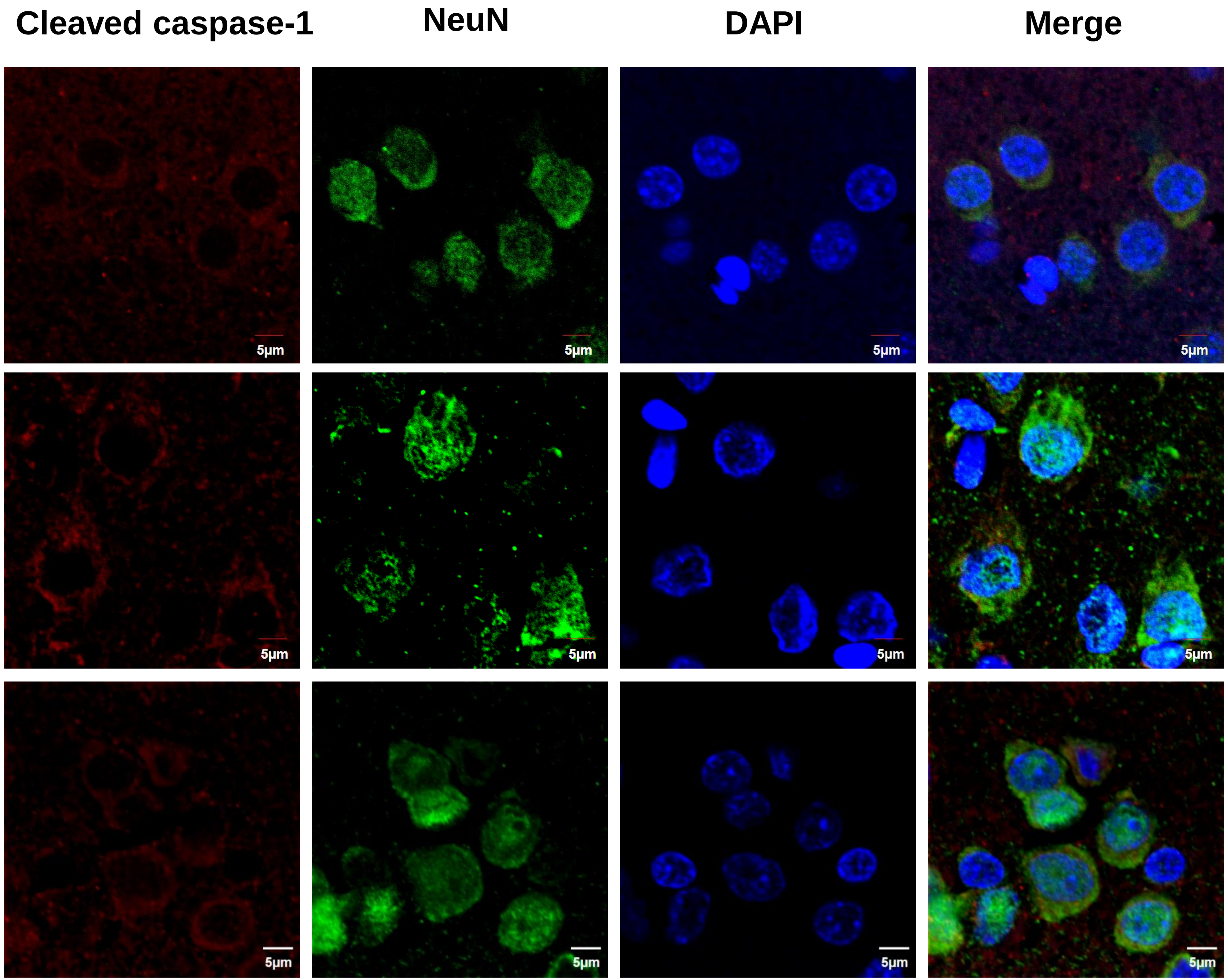
Cleaved caspase-1
NeuN
DAPI
Merge
5μm

Figure5D

Sham:

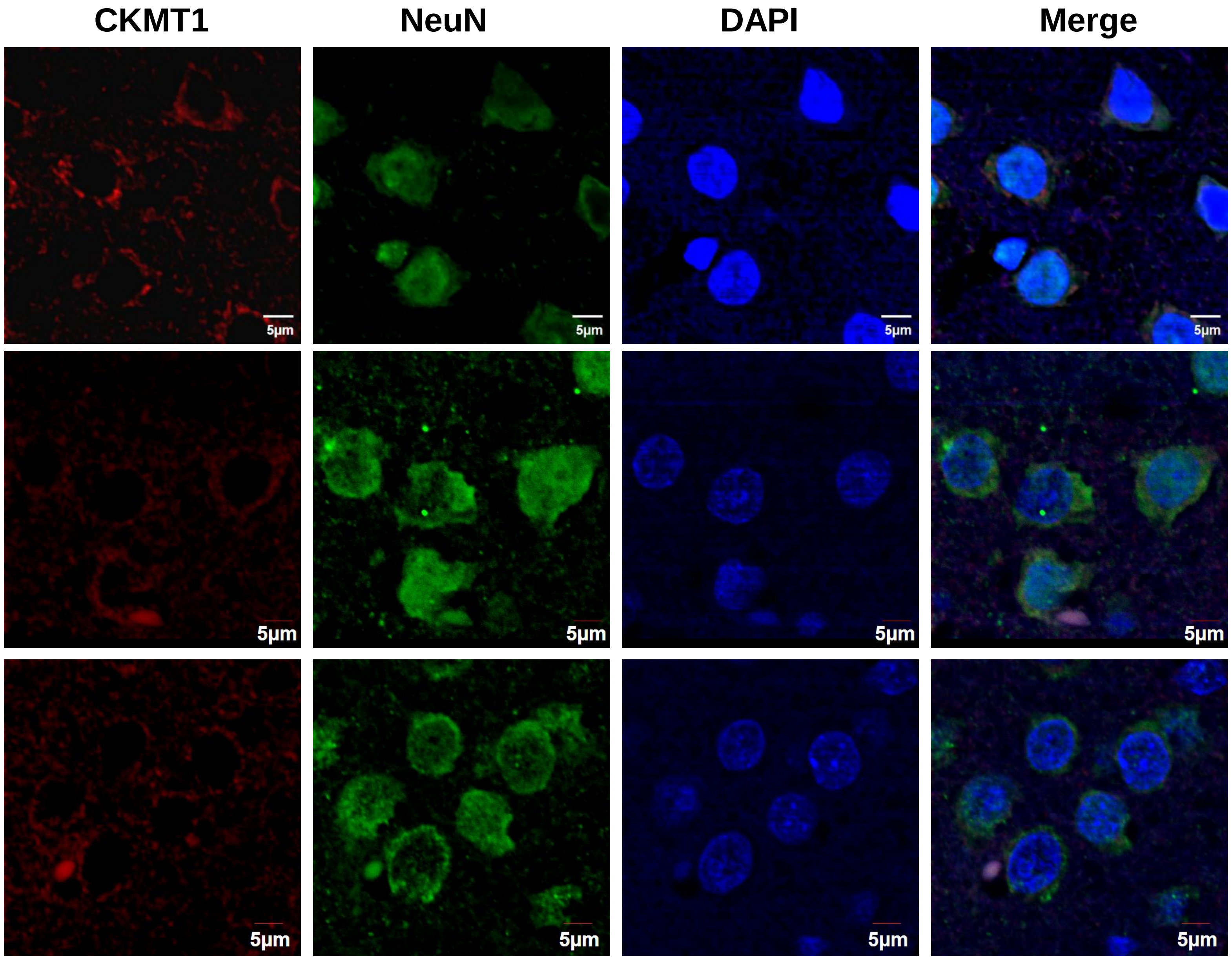

Figure5D

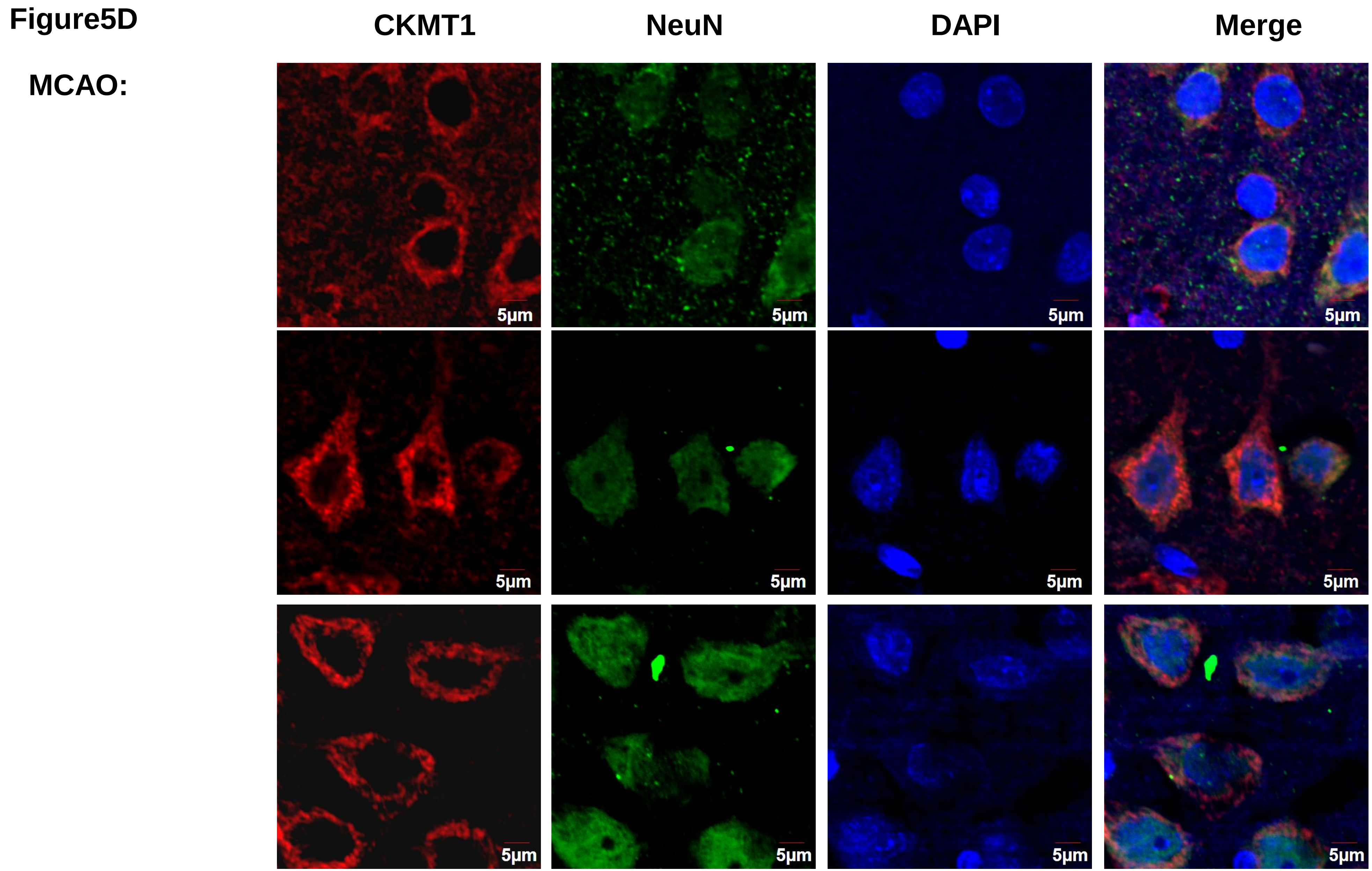

Figure6C

Sham:

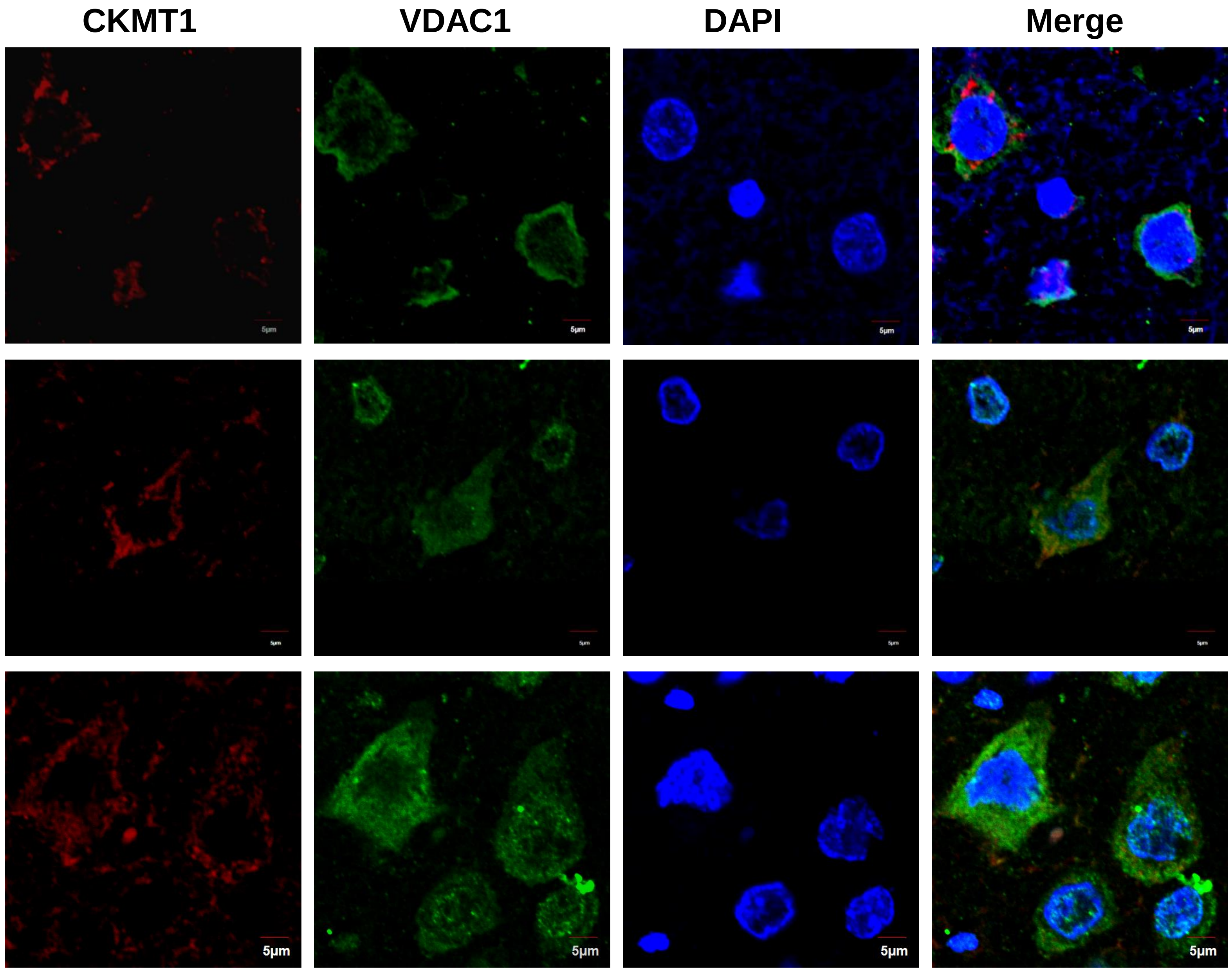

Figure6C

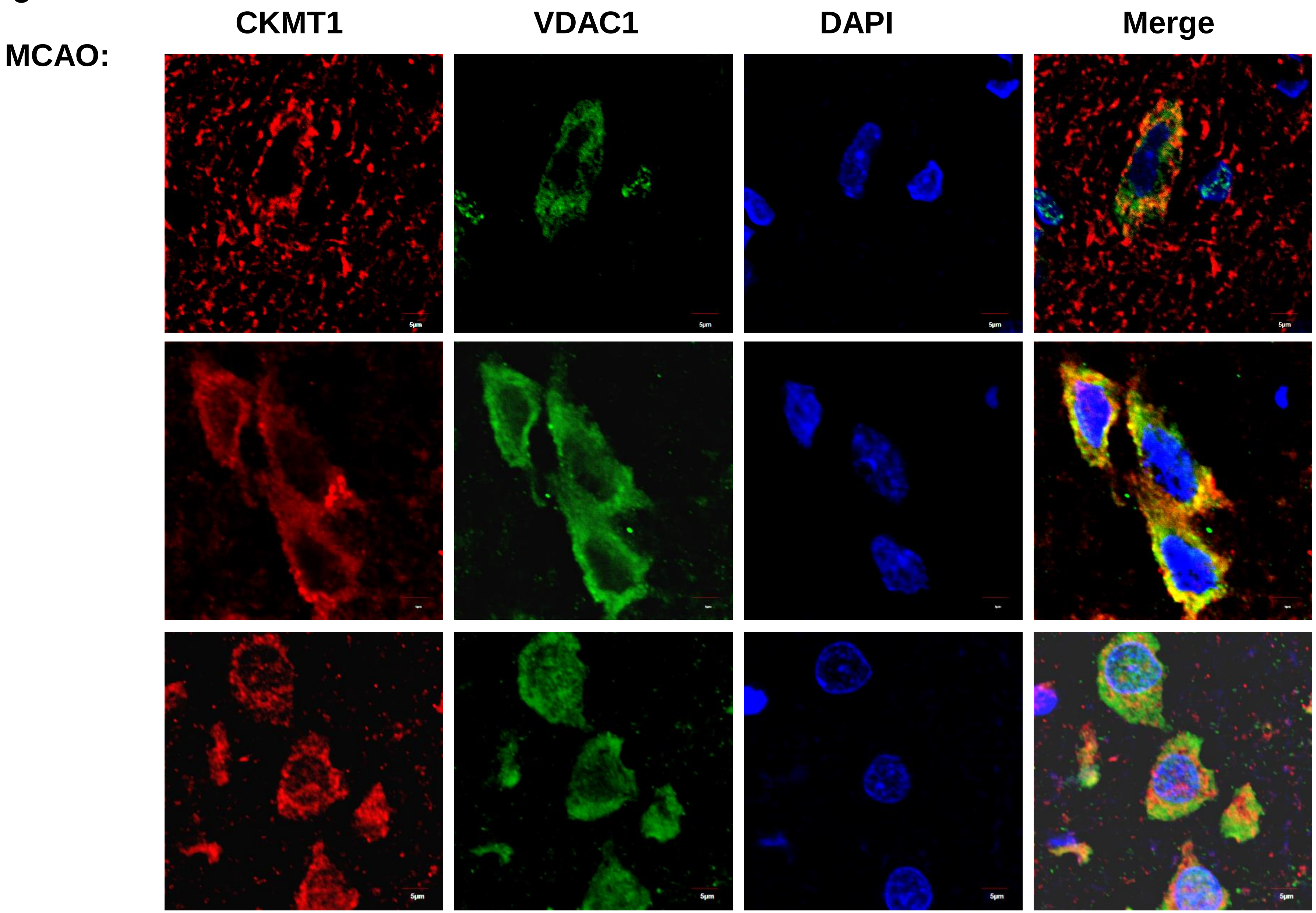

Figure6F

Control: Primary neuron

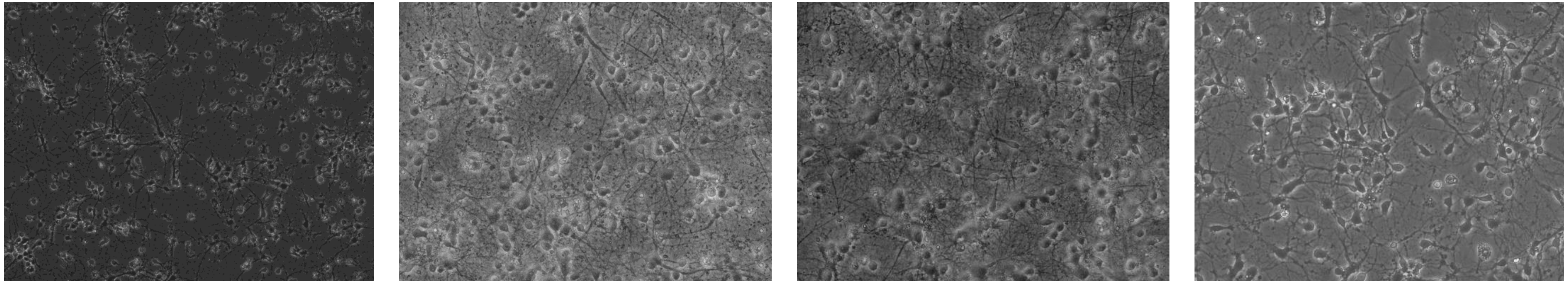

OGD:

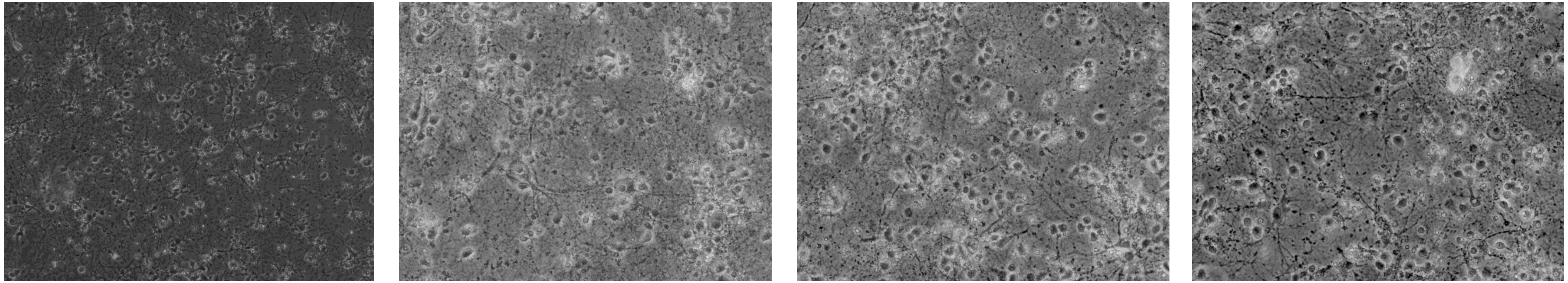

Figure6G

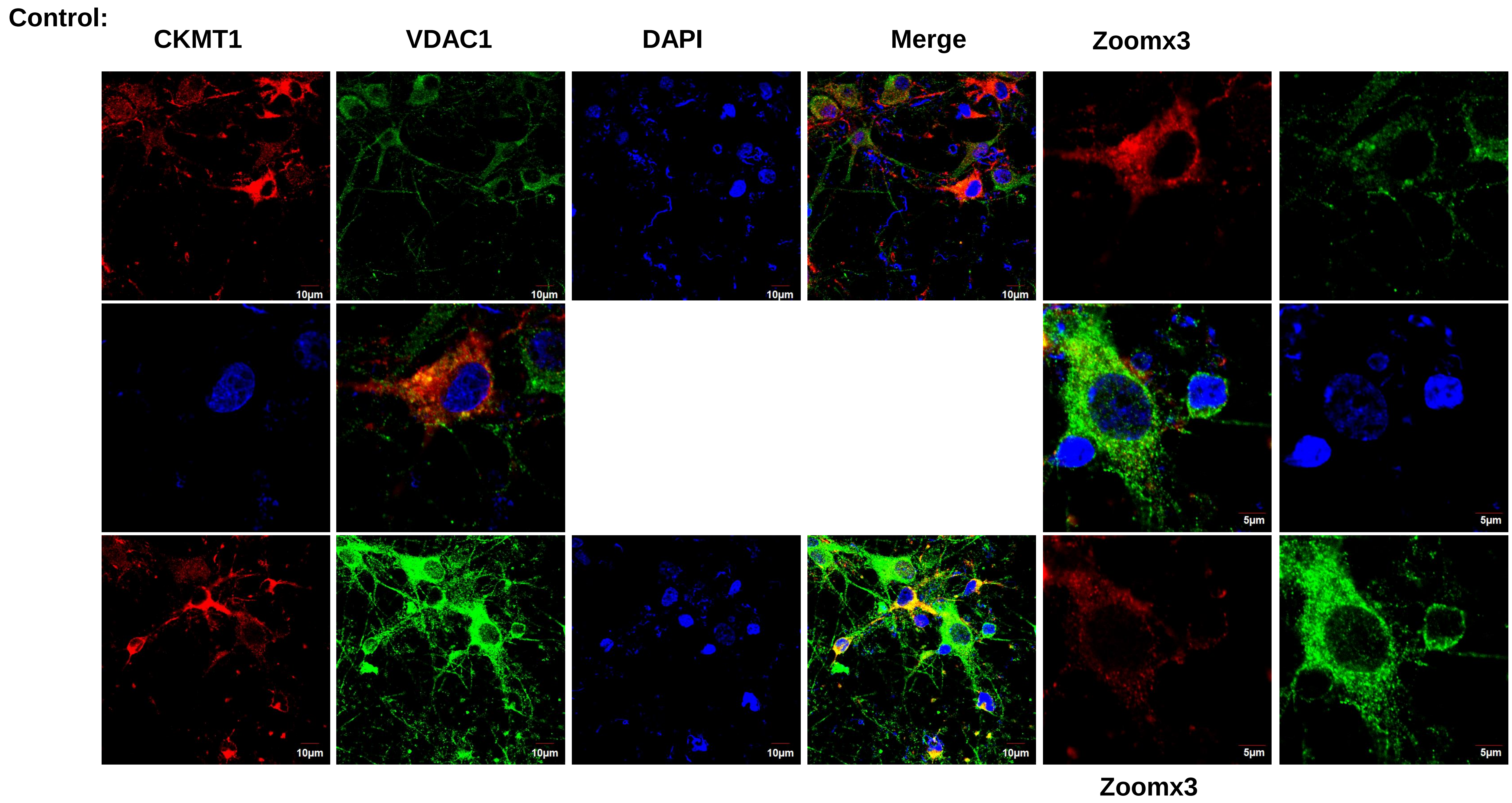

Figure6G

Control:

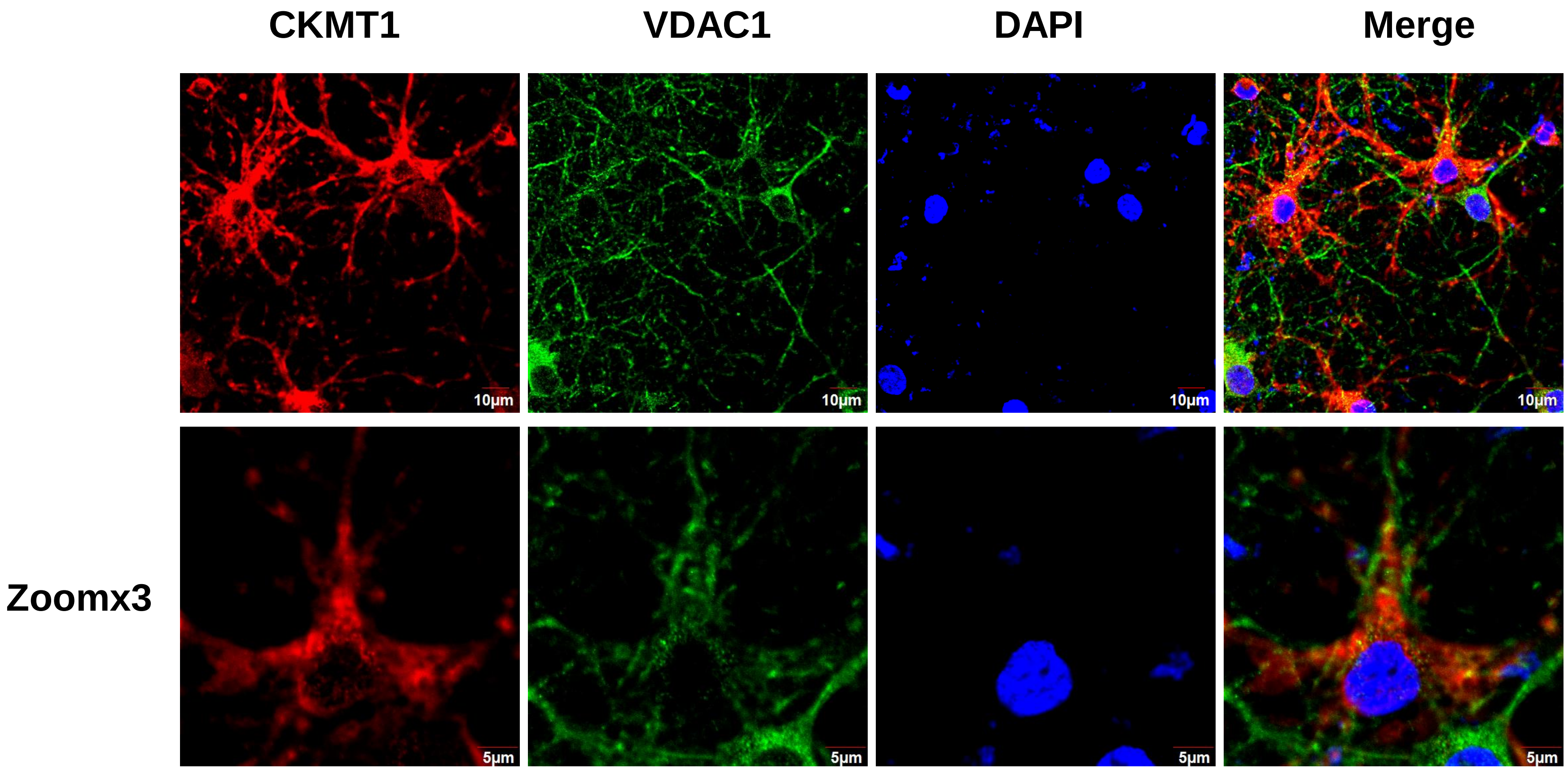

Figure6G

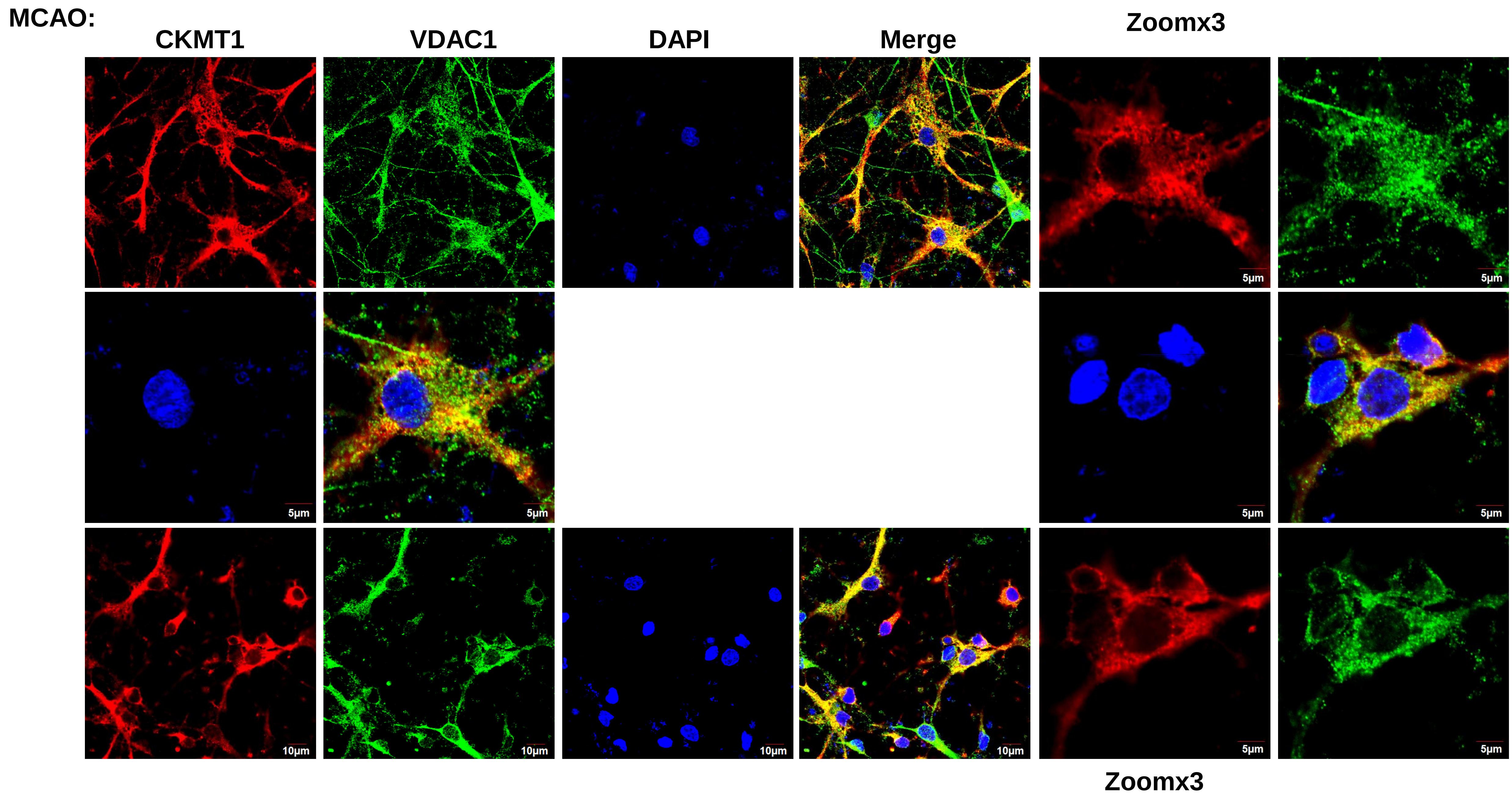

Figure6G

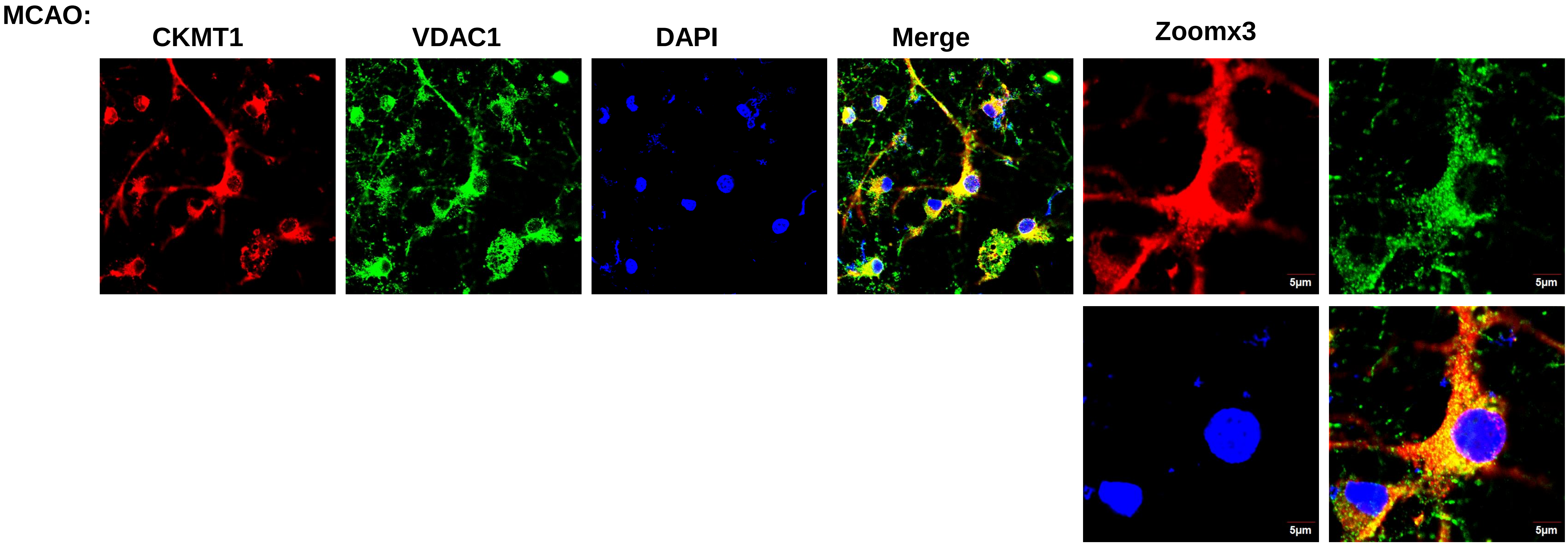

Figure8A

si-NC-EGFP

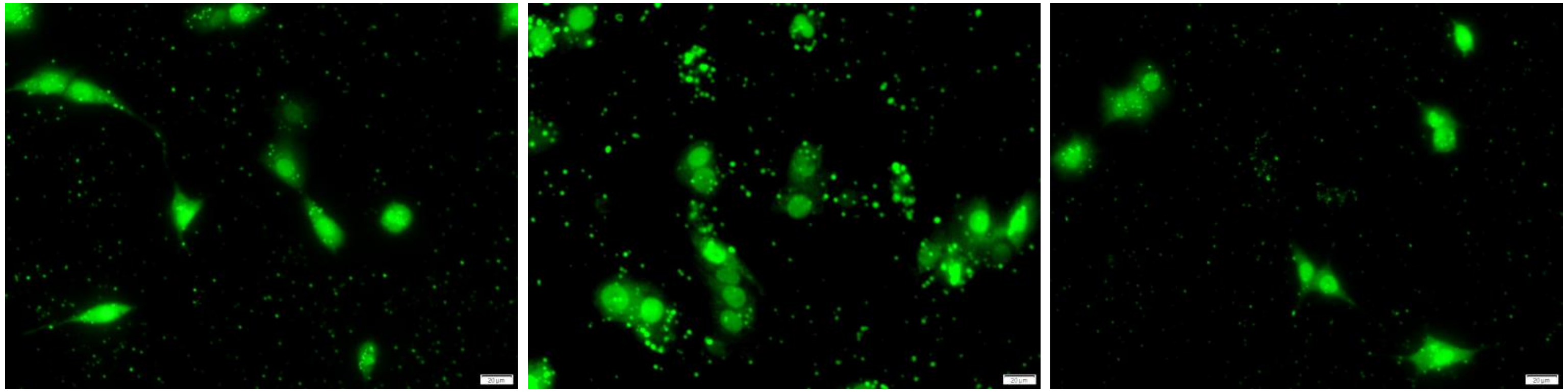

Figure8F

OE-CKMT1-HA：

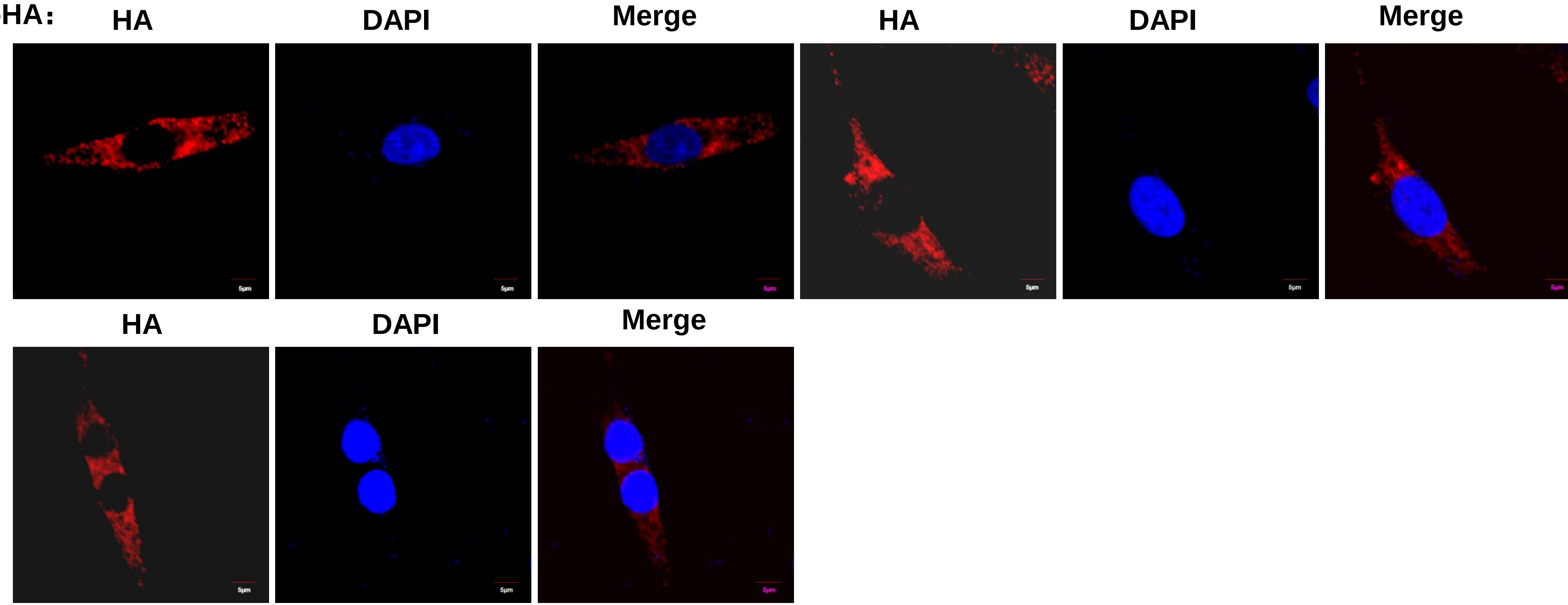

Figure9D

Nissl staining

MCAO:

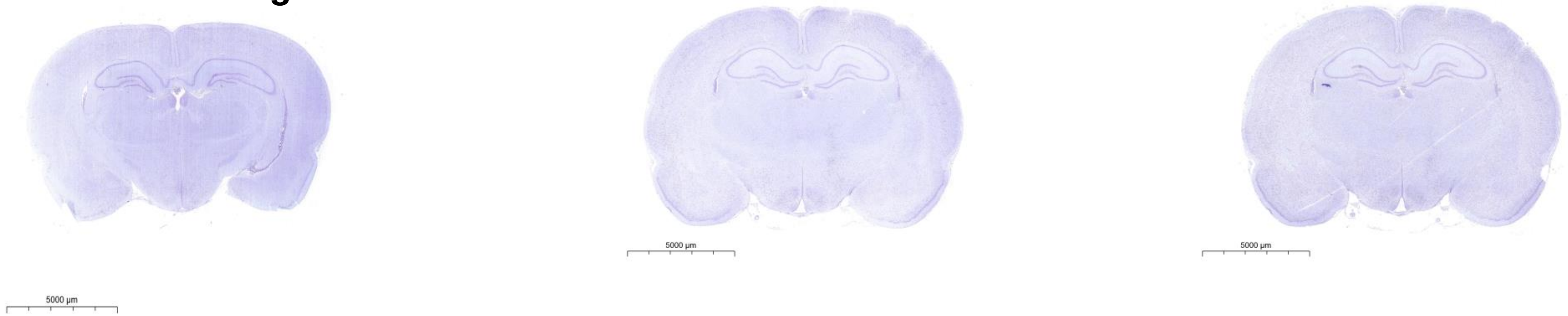

AAV-Control+MCAO:

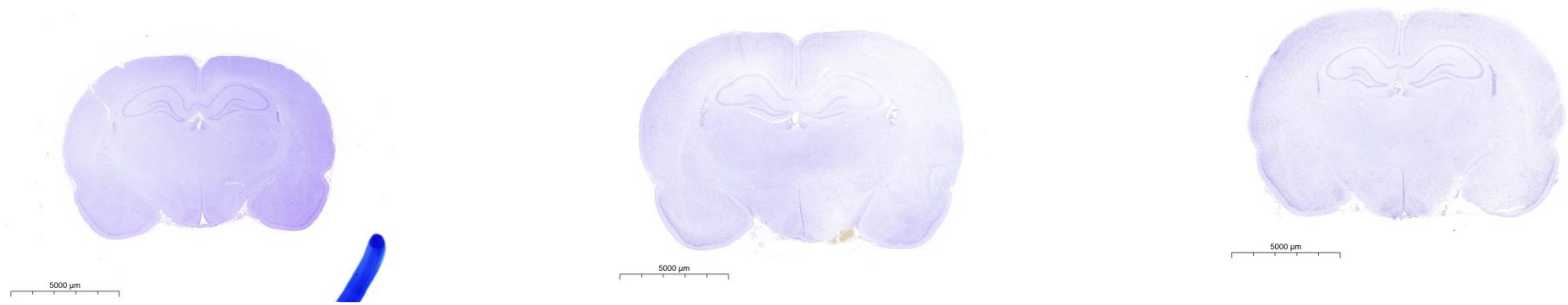

Figure9D Nissl staining

AAV-CKMT1+MCAO:

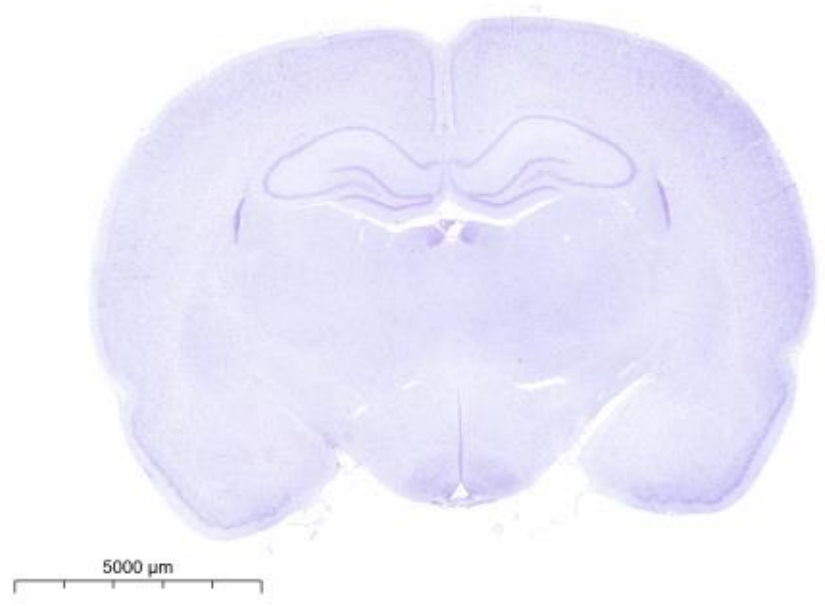

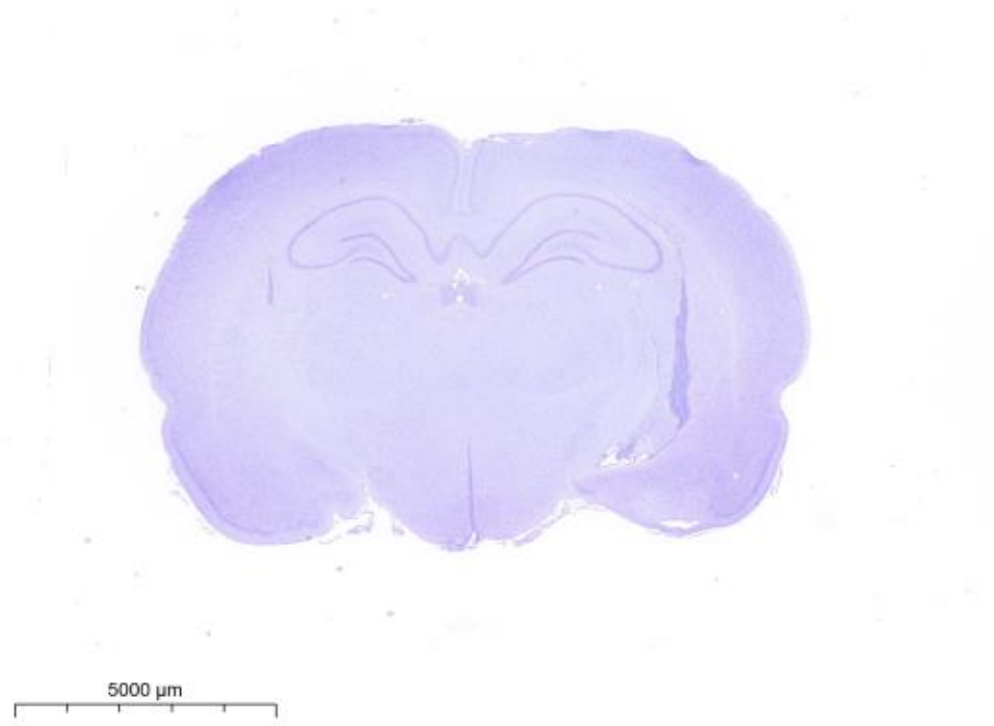

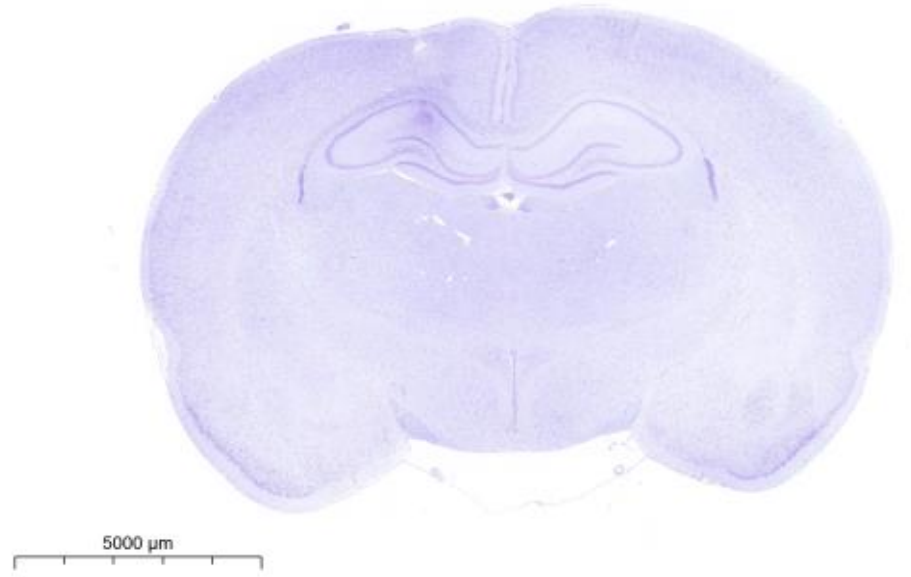

Supplement: Supplementary file 1 [file ADVS-9999-e78611-s003.pdf]
