# Supplementary material for: CKMT1 Counteracts VDAC1 Oligomerization‐Induced Neuronal Pyroptosis Through Steric Hindrance in Ischemic Stroke
Source: Adv Sci (Weinh). 2026 Nov 5:e78611. Online ahead of print. doi: 10.1002/advs.78611 (PMC13735431; doi:10.1002/advs.78611)

Figure 1 G

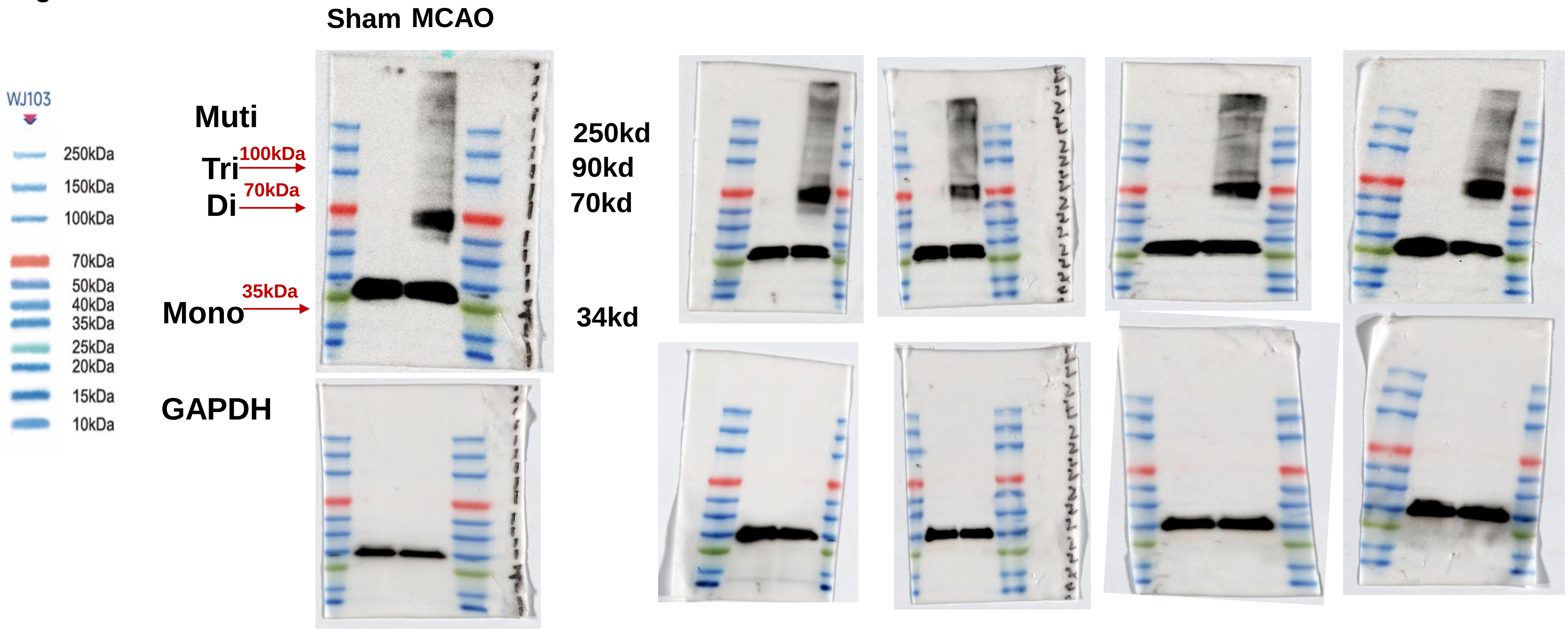
Sham MCAO
WJ103
250kDa
150kDa
100kDa
70kDa
50kDa
40kDa
35kDa
25kDa
20kDa
15kDa
10kDa
Muti
Tri
100kDa
Di
70kDa
35kDa
Mono
GAPDH
250kd
90kd
70kd
34kd

Figure 1 I

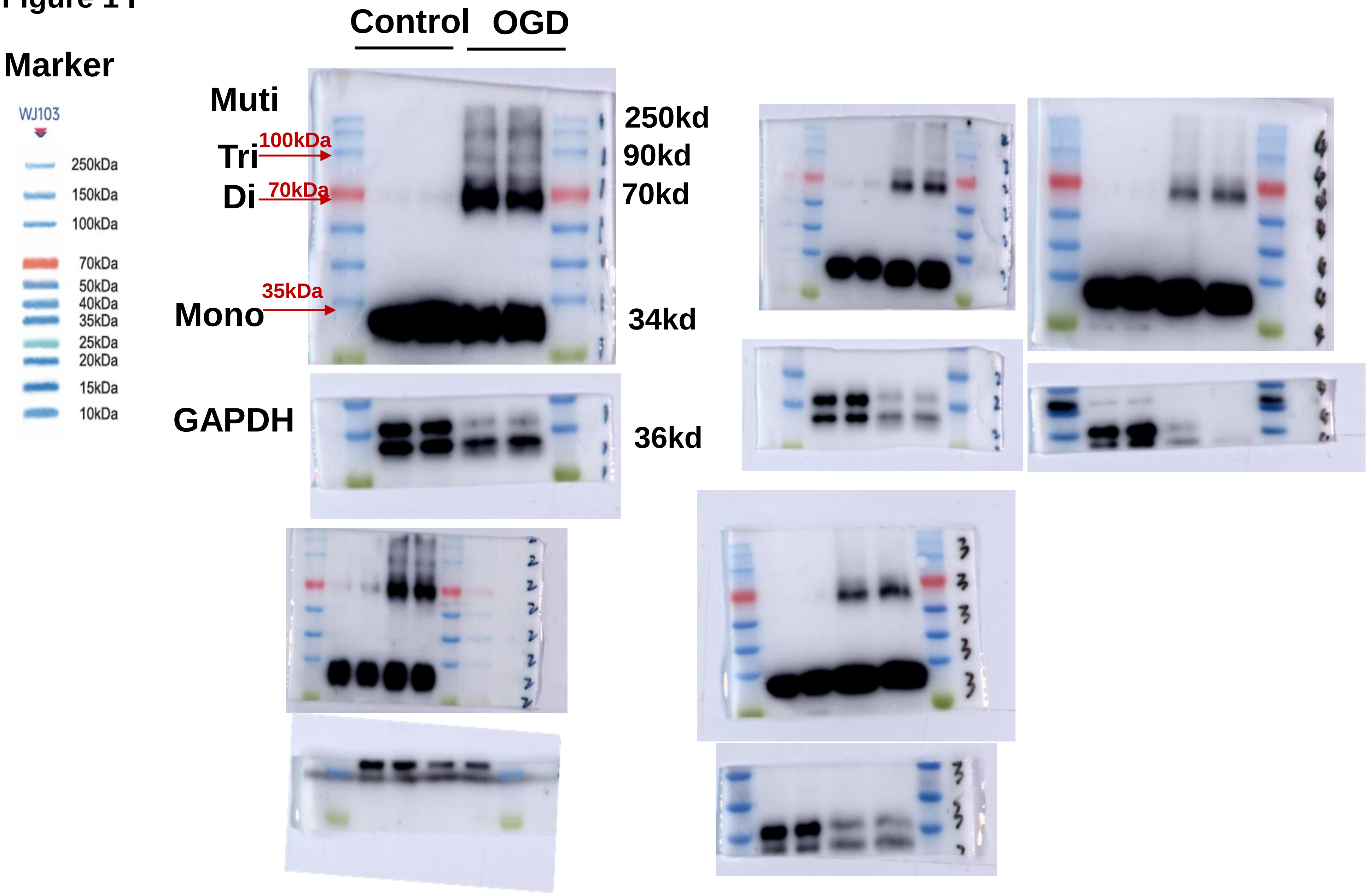
Control
OGD
Marker
WJ103
250kDa
150kDa
100kDa
70kDa
50kDa
40kDa
35kDa
25kDa
20kDa
15kDa
10kDa
Muti
Tri
100kDa
Di
70kDa
35kDa
Mono
GAPDH
250kd
90kd
70kd
34kd
36kd

Figure 1 L

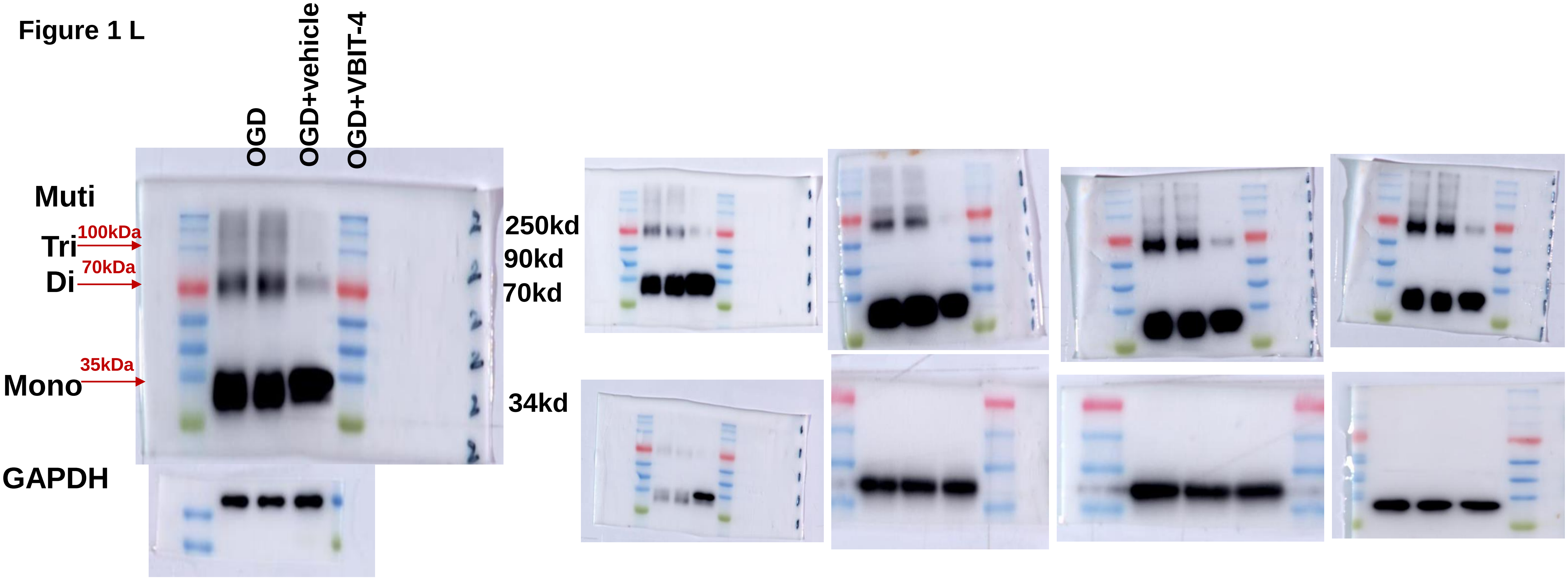

GAPDH was detected from the same sample without oligomerization treatment, as oligomerization impaired GAPDH signal.

Figure 2E

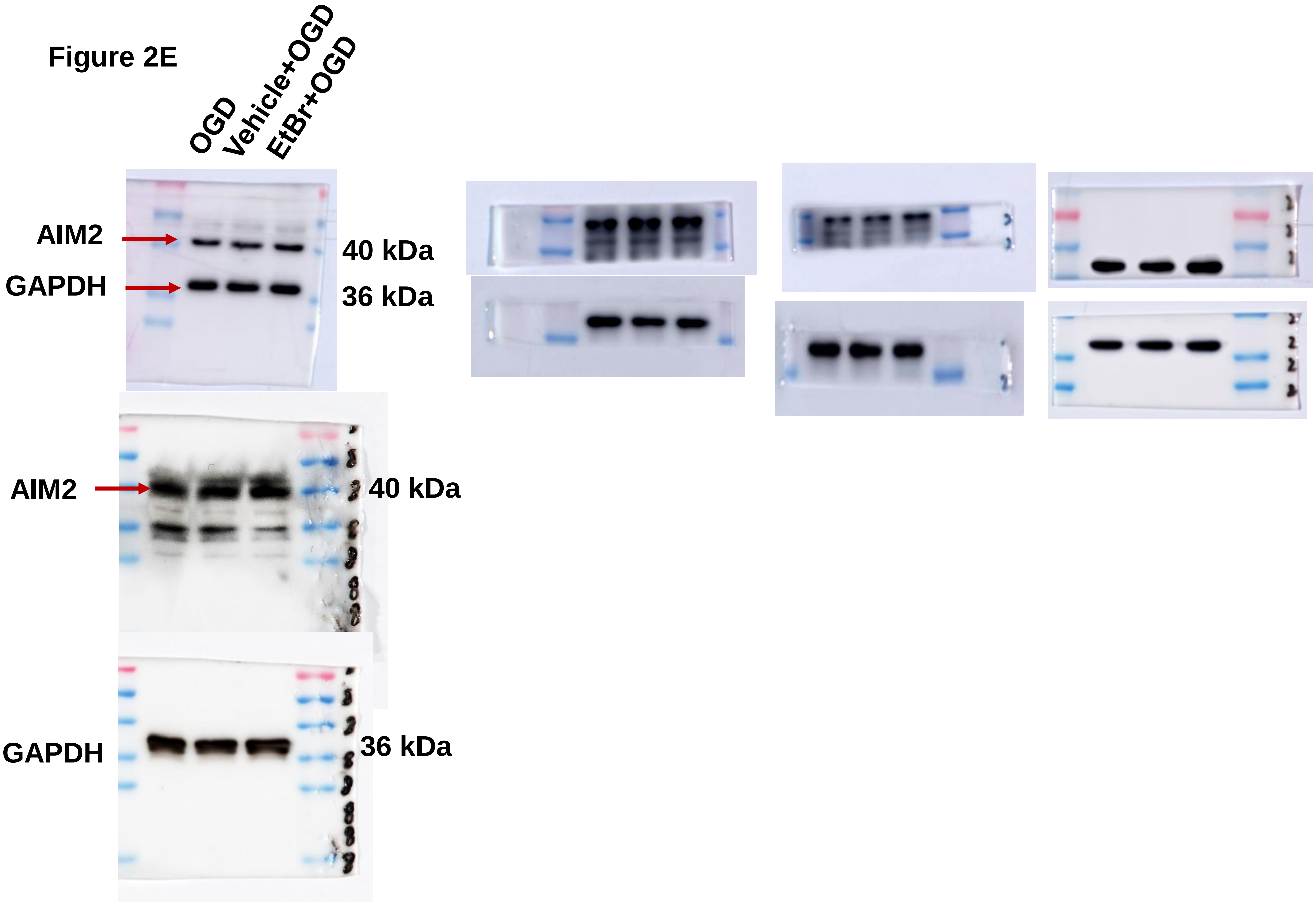
OGD
Vehicle+OGD
EtBr+OGD
AIM2
40 kDa
GAPDH
36 kDa
AIM2
40 kDa
GAPDH
36 kDa

Figure2E

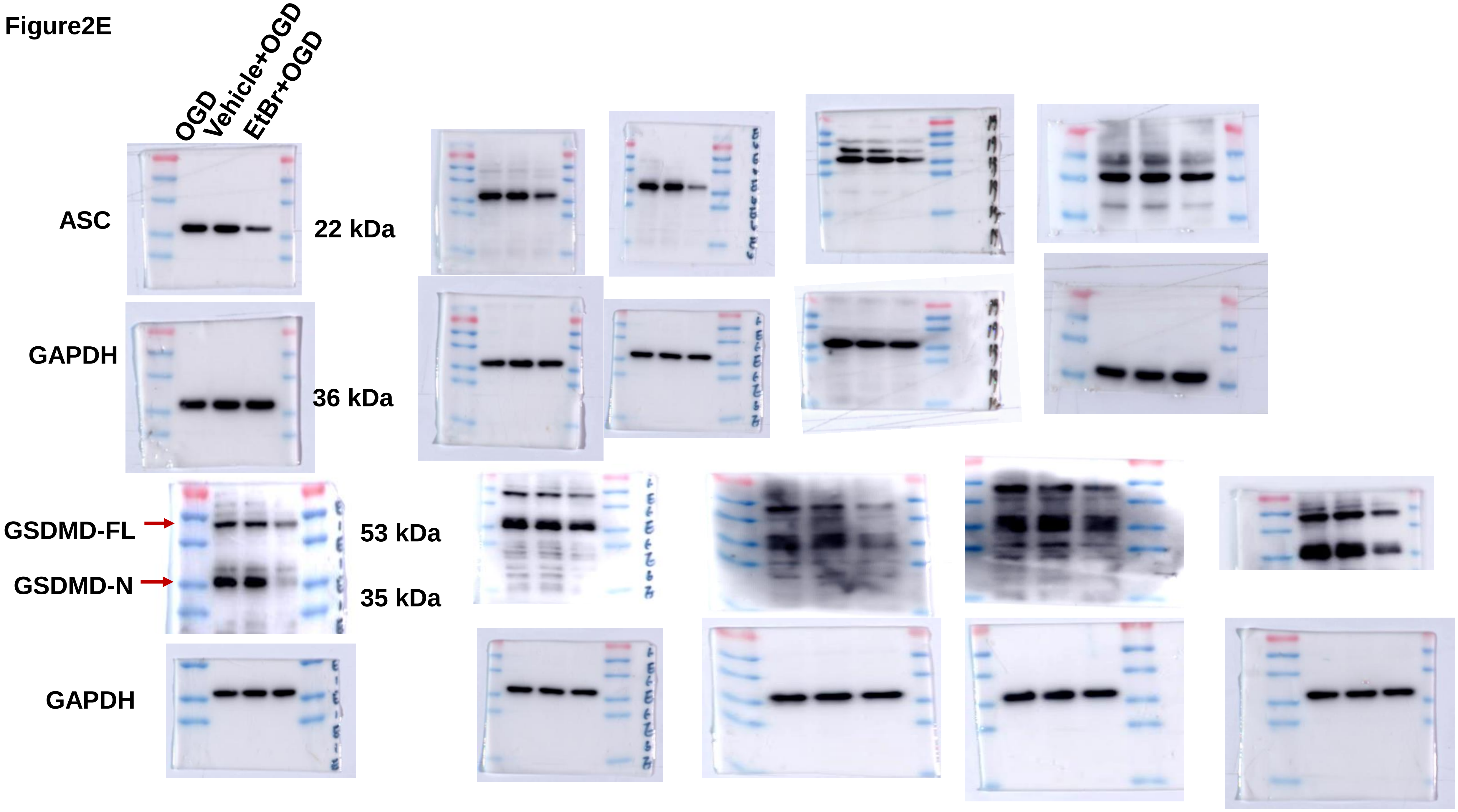
OGD
Vehicle+OGD
EtBr+OGD
ASC
22 kDa
GAPDH
36 kDa
GSDMD-FL
GSDMD-N
53 kDa
35 kDa
GAPDH

Figure2E

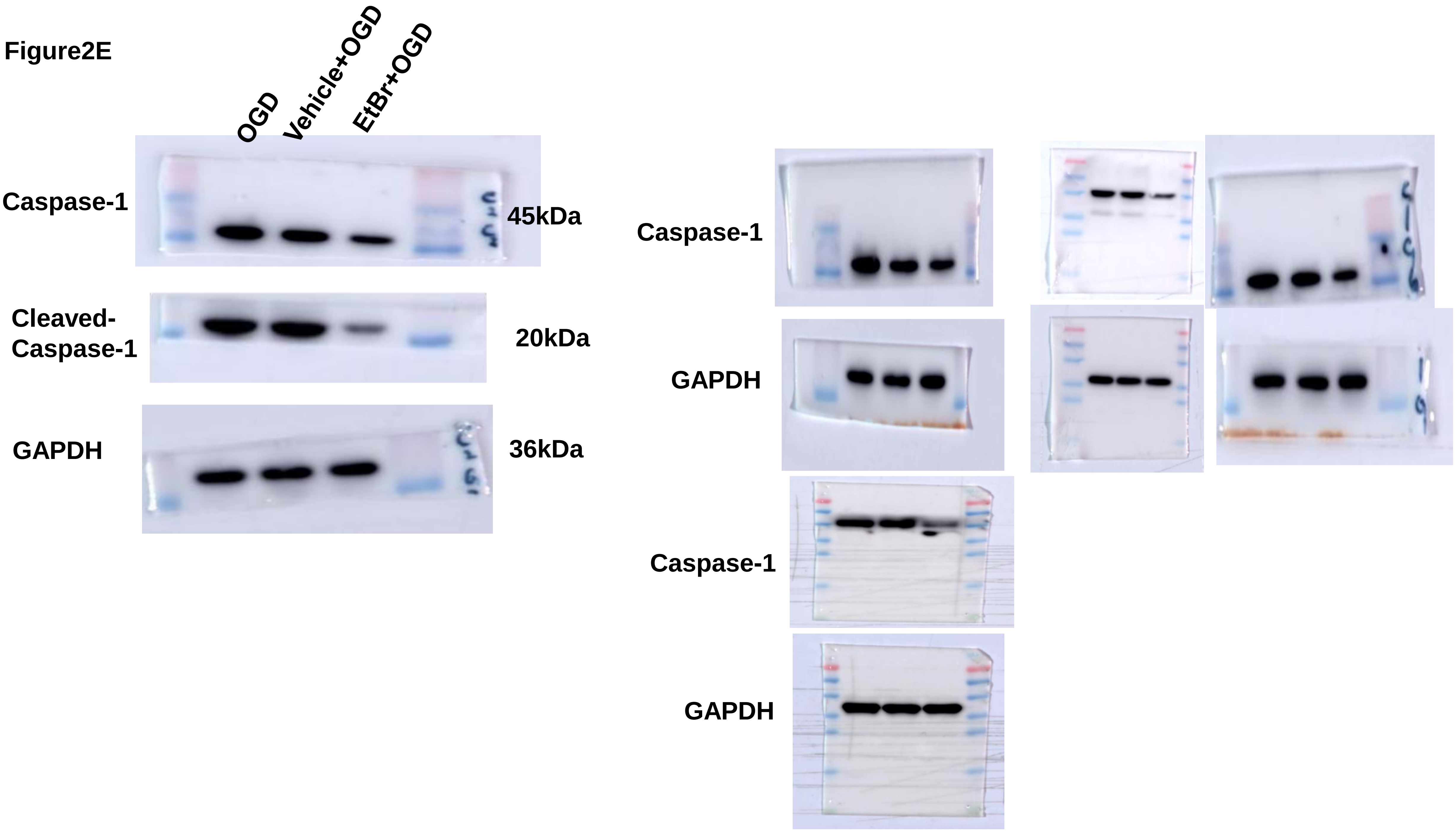

OGD
Vehicle+OGD
EtBr+OGD
Caspase-1
45kDa
Cleaved-Caspase-1
20kDa
GAPDH
36kDa
Caspase-1
GAPDH
Caspase-1
GAPDH

Figure2E

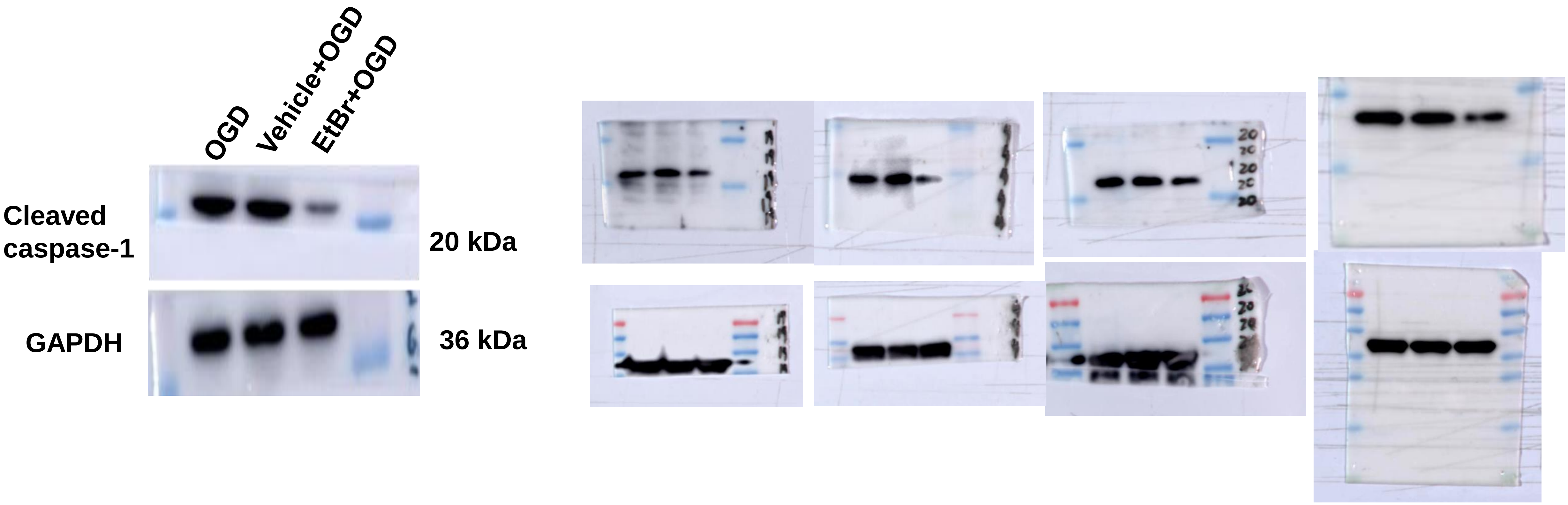
OGD
Vehicle+OGD
EtBr+OGD
Cleaved caspase-1
GAPDH
20 kDa
36 kDa

Figure3 E

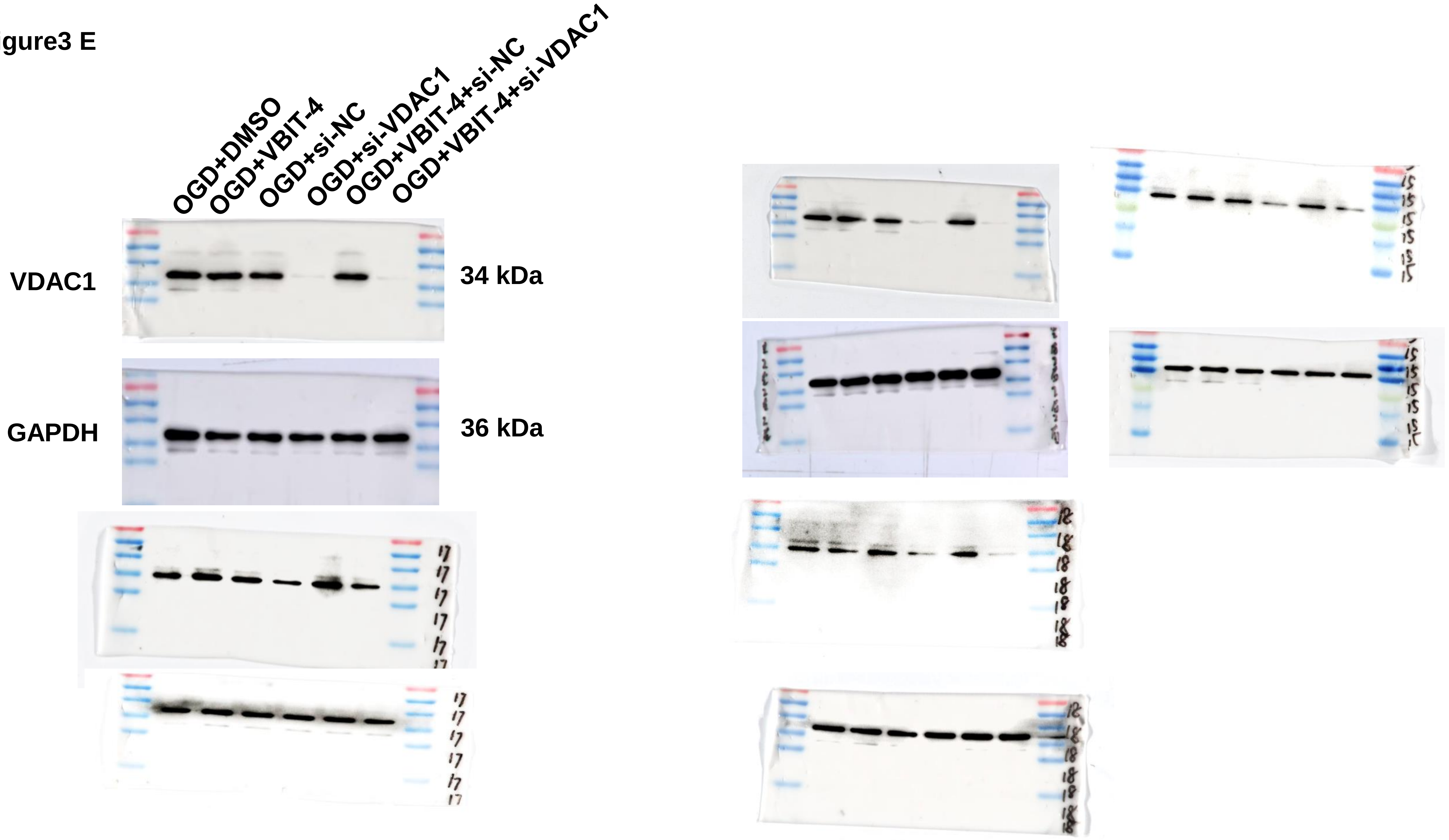
OGD+DMSO
OGD+VBIT-4
OGD+si-NC
OGD+si-VDAC1
OGD+VBIT-4+si-NC
OGD+VBIT-4+si-VDAC1
VDAC1
34 kDa
GAPDH
36 kDa

Figure 3H

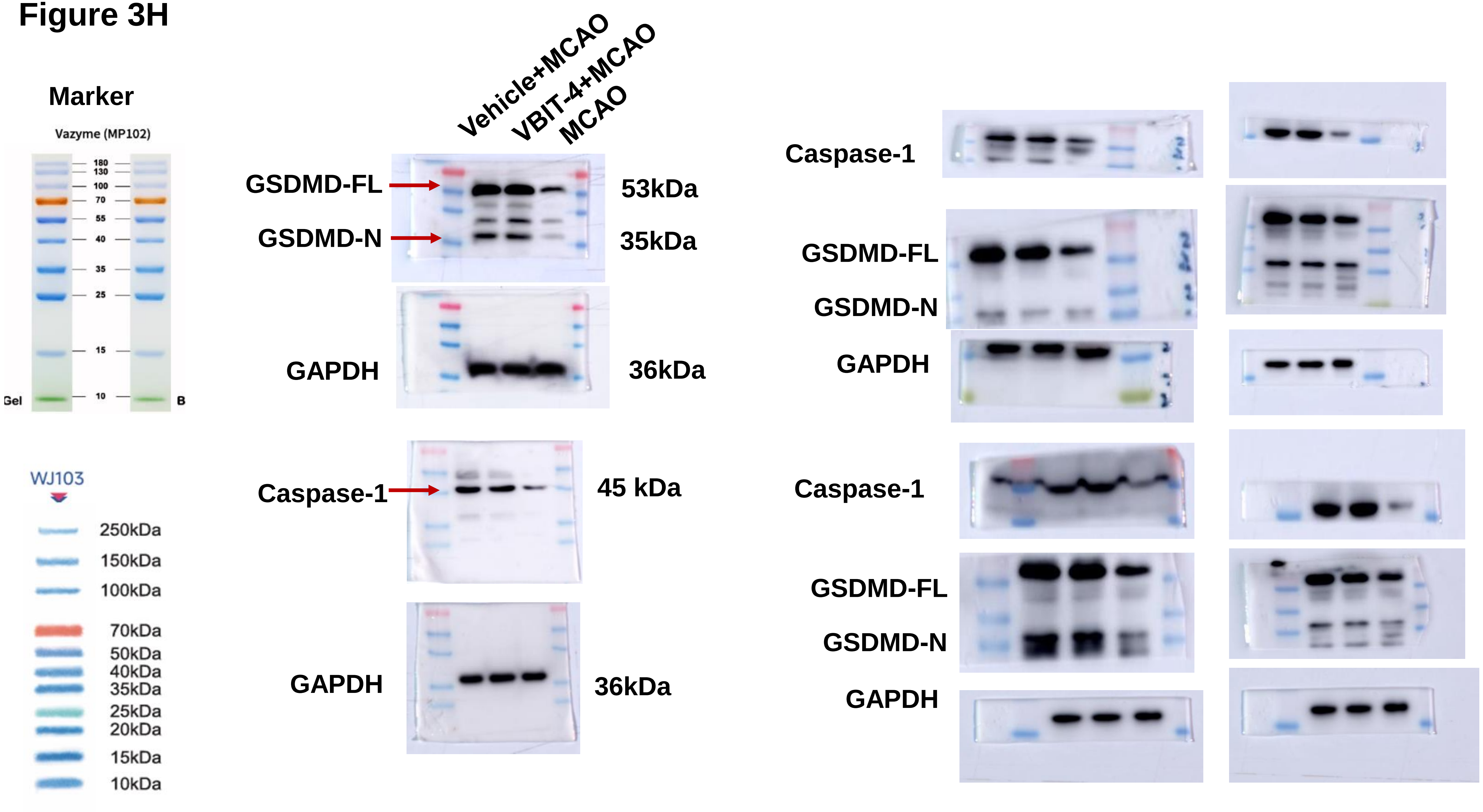
Marker
Vazyme (MP102)
180
130
100
70
55
40
35
25
15
10
Gel
B
WJ103
250kDa
150kDa
100kDa
70kDa
50kDa
40kDa
35kDa
25kDa
20kDa
15kDa
10kDa
Vehicle+MCAO
VBIT-4+MCAO
MCAO
GSDMD-FL
GSDMD-N
53kDa
35kDa
GAPDH
36kDa
Caspase-1
45 kDa
GAPDH
36kDa
Caspase-1
GSDMD-FL
GSDMD-N
GAPDH
Caspase-1
GSDMD-FL
GSDMD-N
GAPDH

Figure 3H

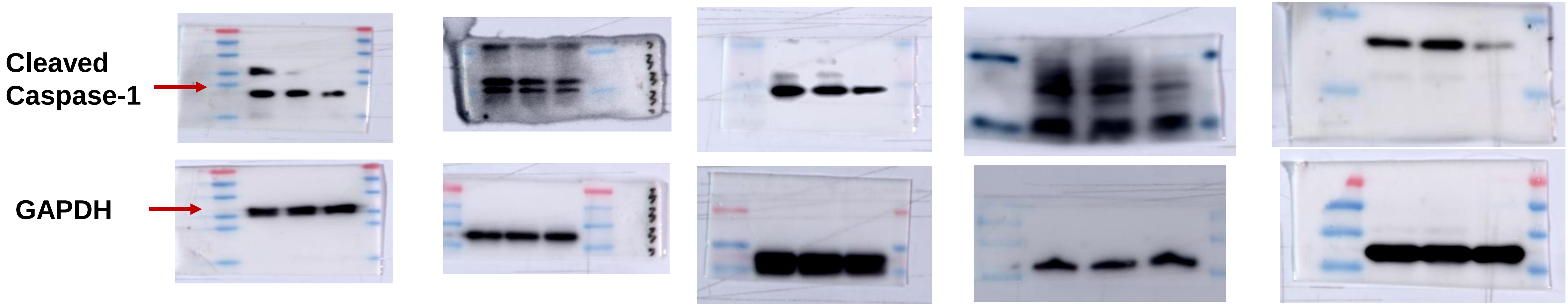

Figure5A

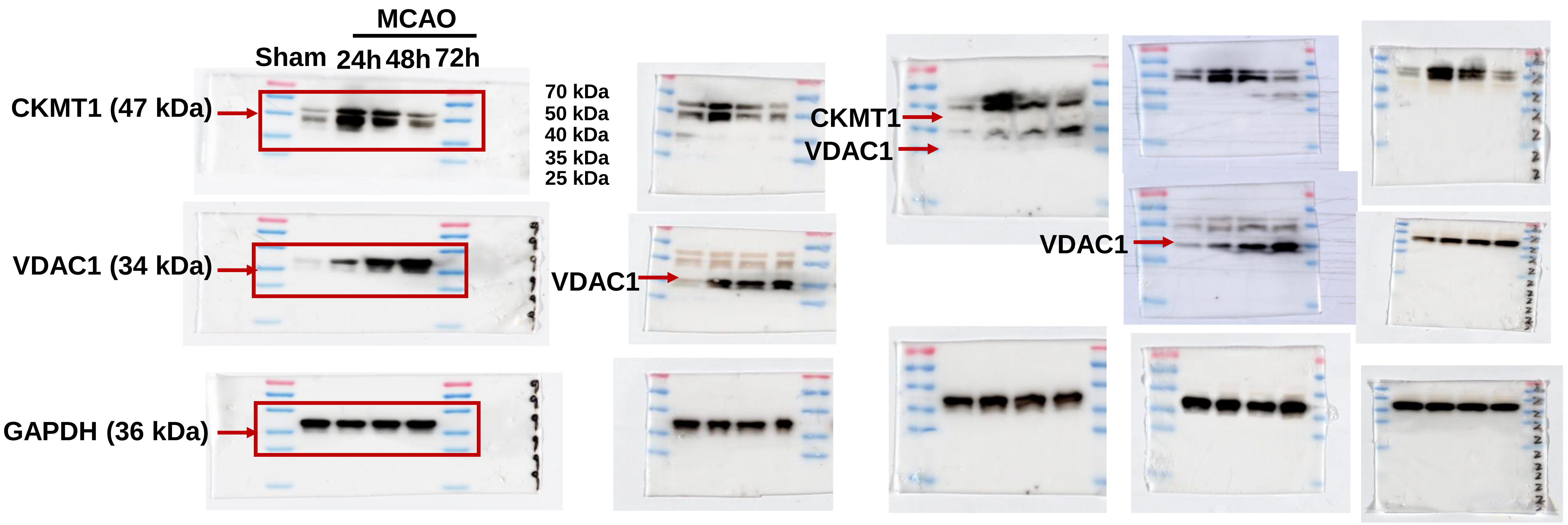
MCAO
Sham
24h
48h
72h
CKMT1 (47 kDa)
VDAC1 (34 kDa)
GAPDH (36 kDa)
70 kDa
50 kDa
40 kDa
35 kDa
25 kDa
VDAC1
CKMT1
VDAC1
VDAC1

Figure5A

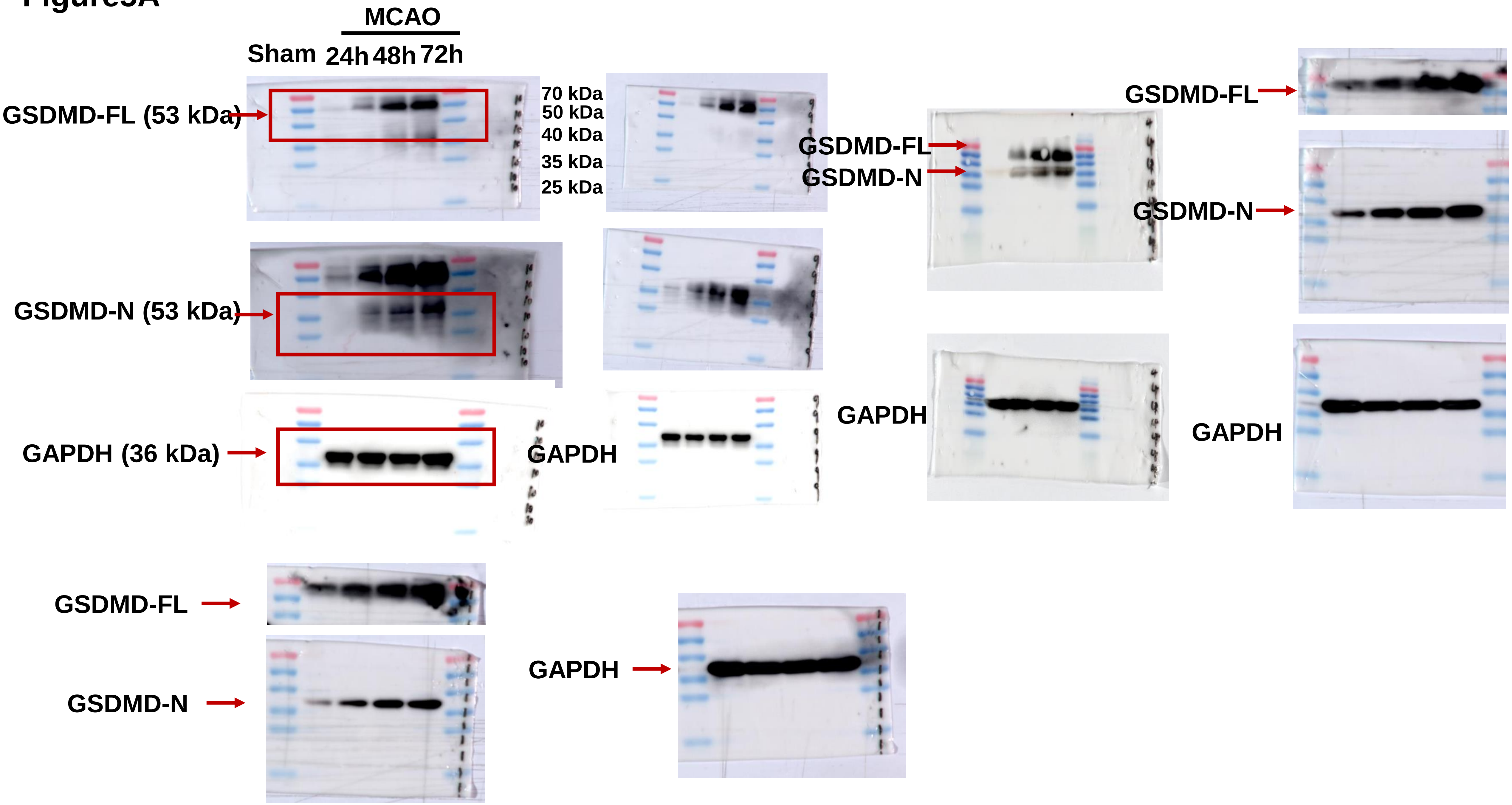
MCAO
Sham
24h
48h
72h
GSDMD-FL (53 kDa)
GSDMD-N (53 kDa)
GAPDH (36 kDa)
70 kDa
50 kDa
40 kDa
35 kDa
25 kDa
GSDMD-FL
GSDMD-N
GAPDH
GSDMD-FL
GSDMD-N
GAPDH
GSDMD-FL
GSDMD-N
GAPDH
GSDMD-FL
GSDMD-N
GAPDH

Figure 5B

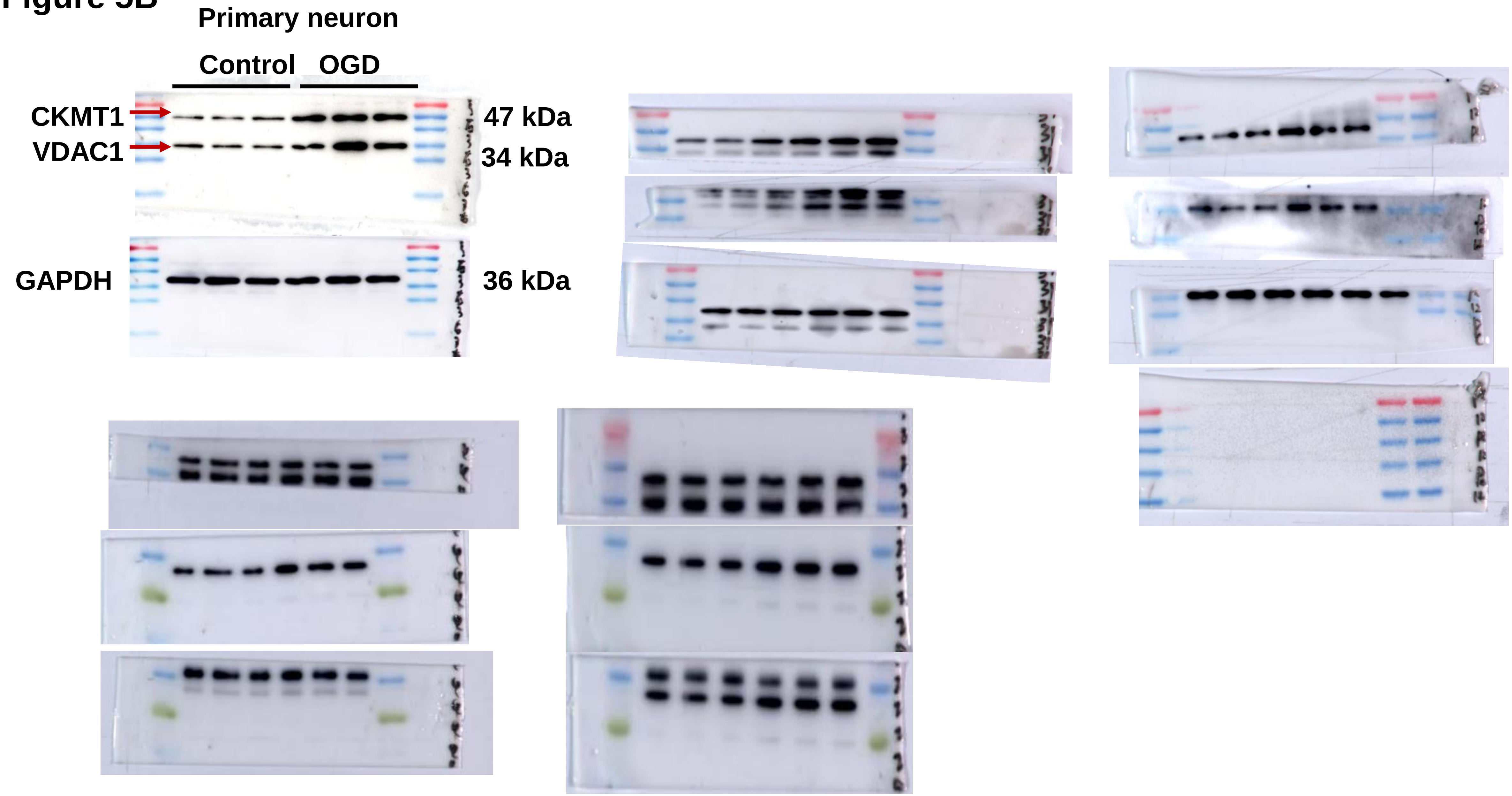
Primary neuron
Control
OGD
CKMT1
VDAC1
47 kDa
34 kDa
GAPDH
36 kDa

Figure 5C

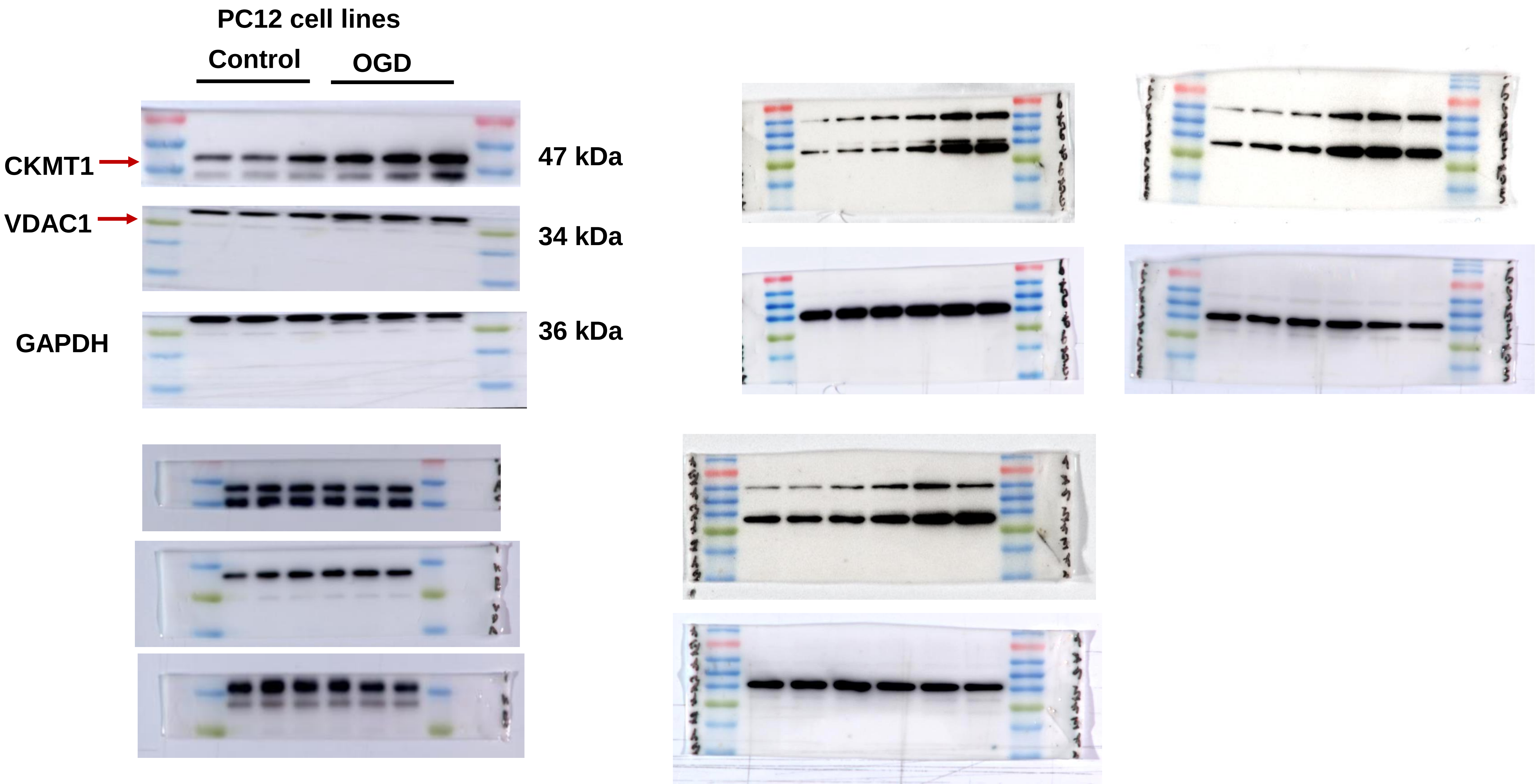
PC12 cell lines
Control
OGD
CKMT1
VDAC1
GAPDH
47 kDa
34 kDa
36 kDa

Figure 5 I

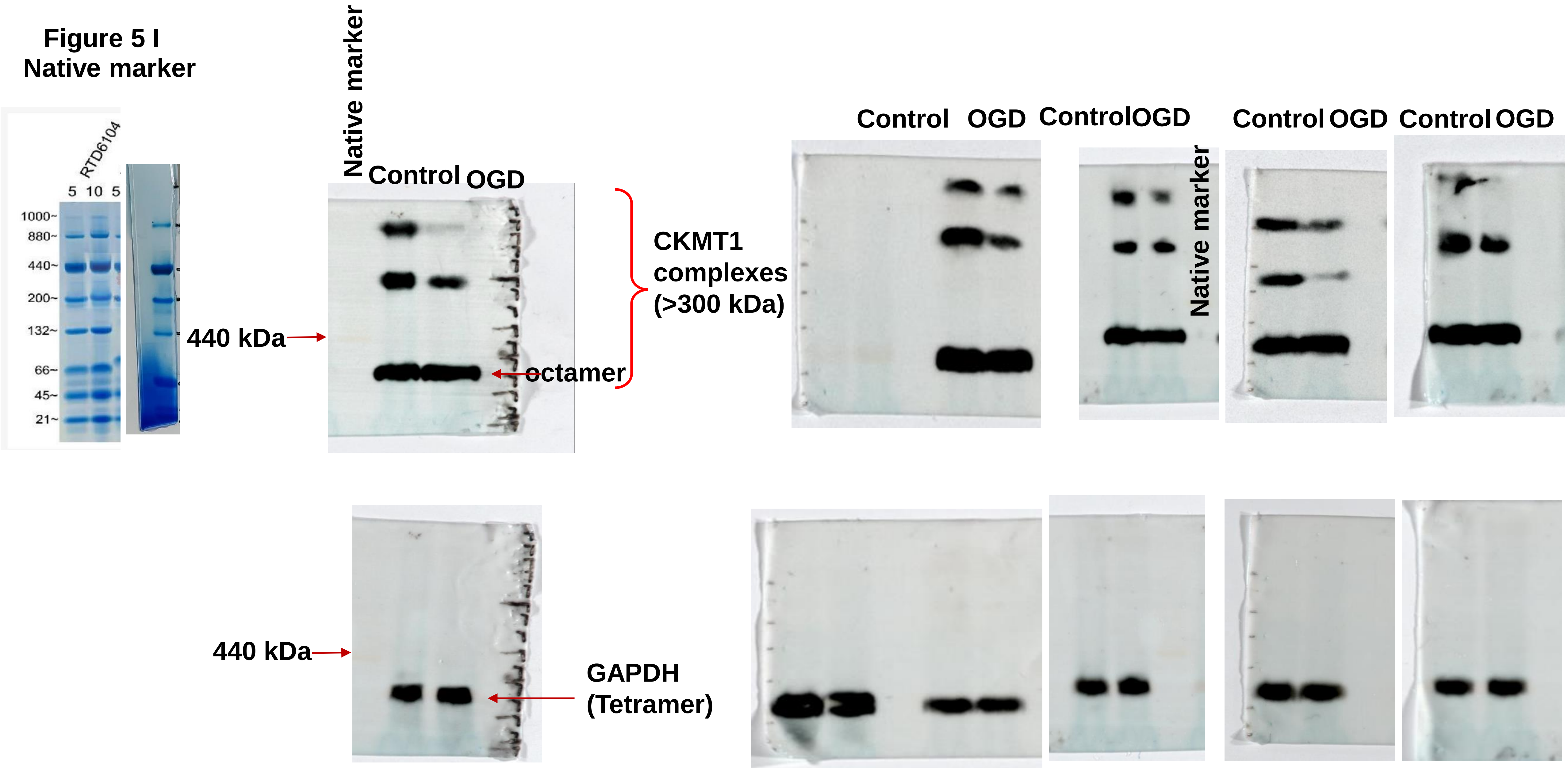
Native marker
RTD6104
5 10 5
1000~
880~
440~
200~
132~
66~
45~
21~
Native marker
Control OGD
440 kDa
octamer
CKMT1
complexes
(>300 kDa)
Control OGD Control OGD Control OGD Control OGD
Native marker
440 kDa
GAPDH
(Tetramer)

Figure 6A

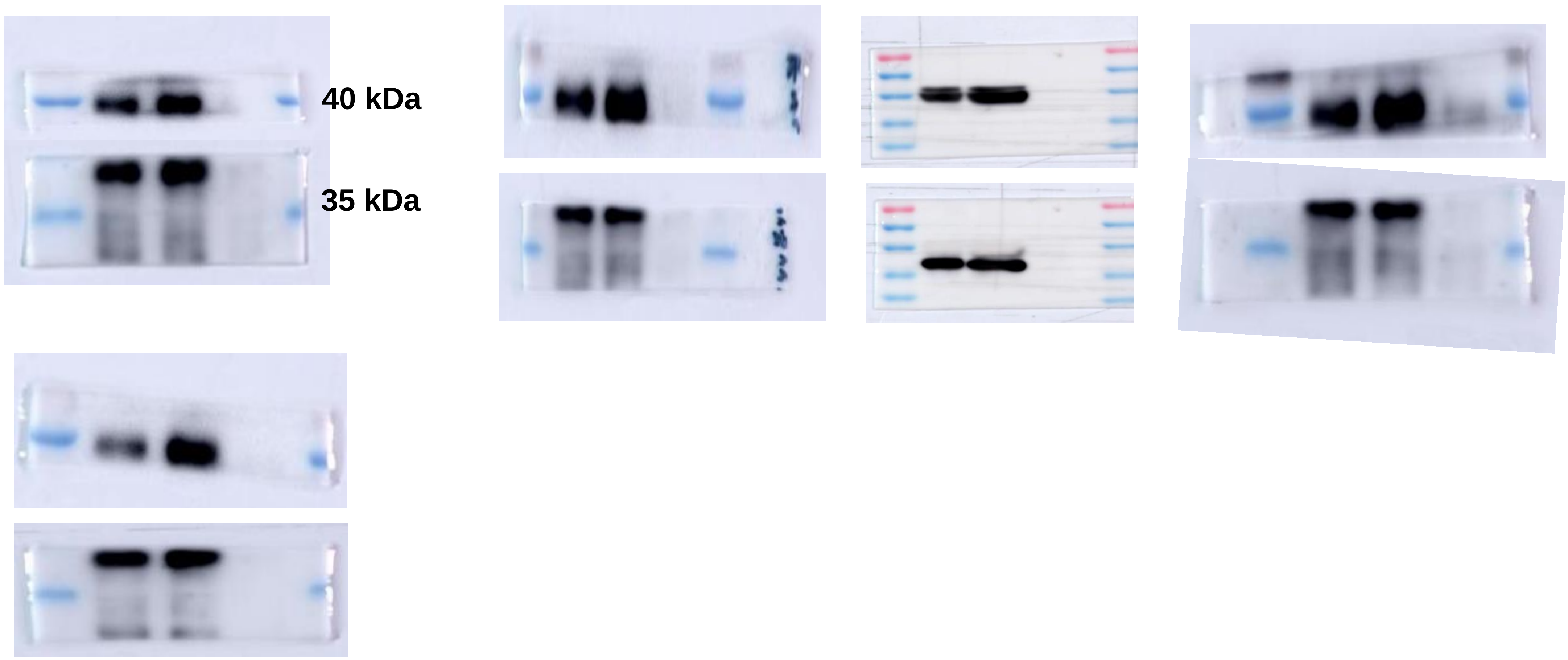

Figure 6B

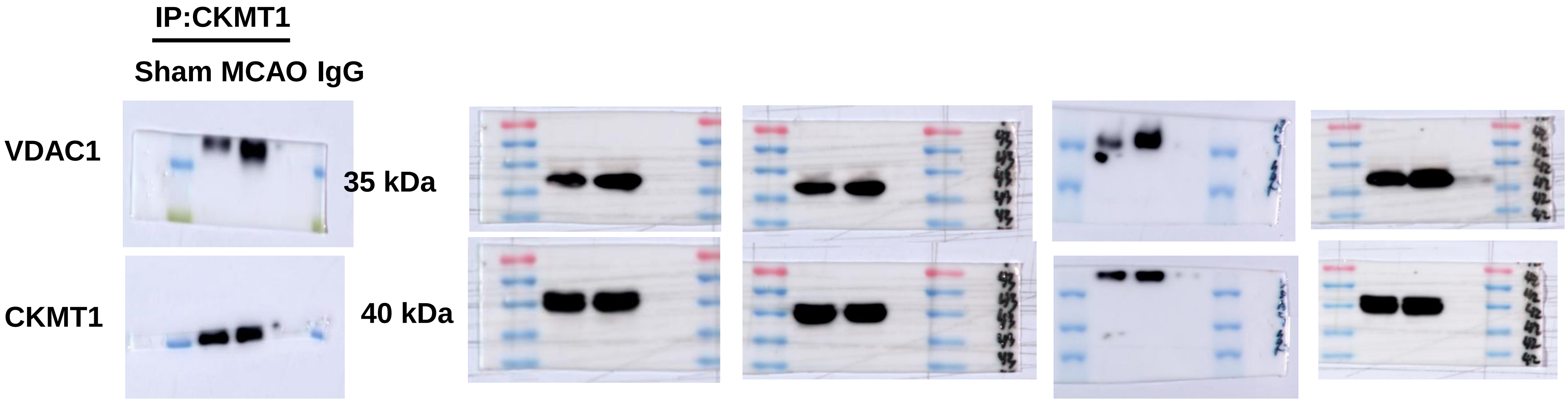

Figure 6A,B

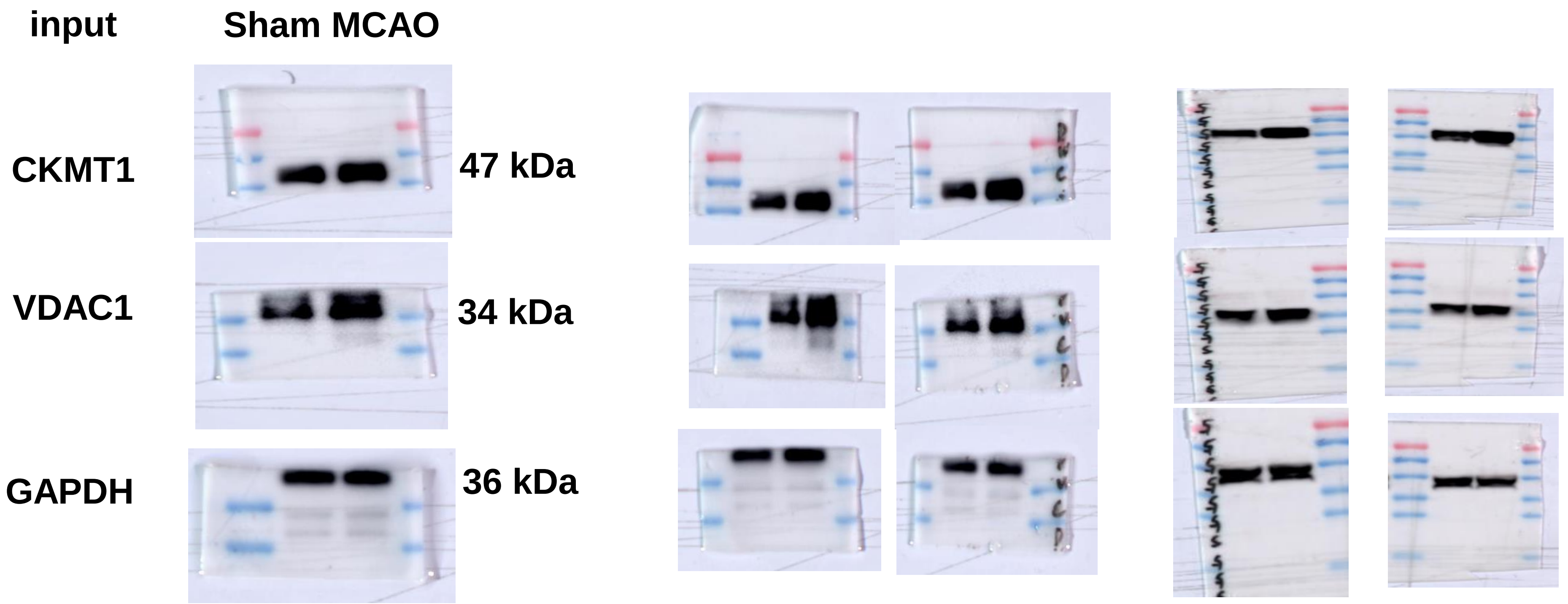

Figure7B

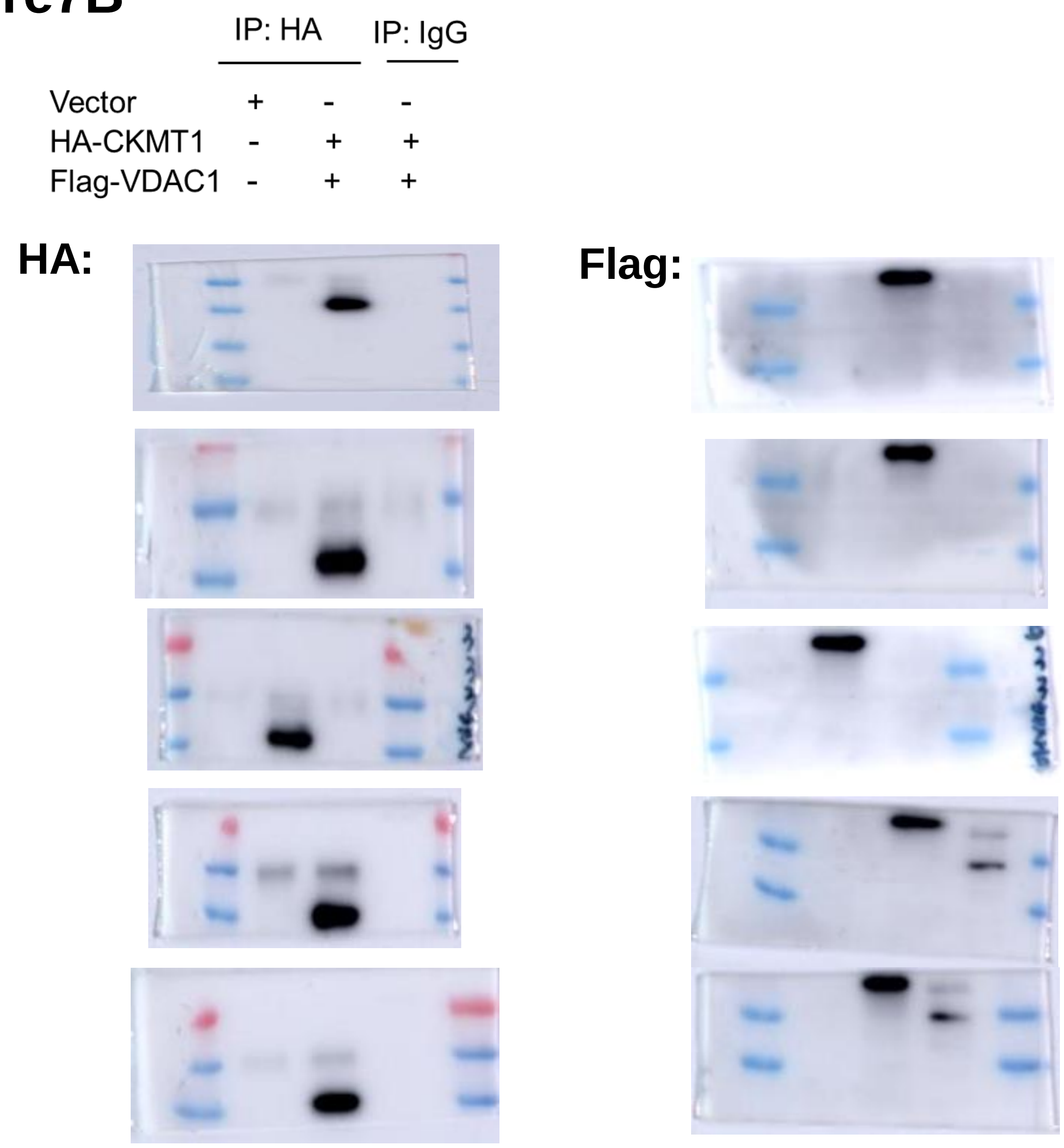

Figure7C

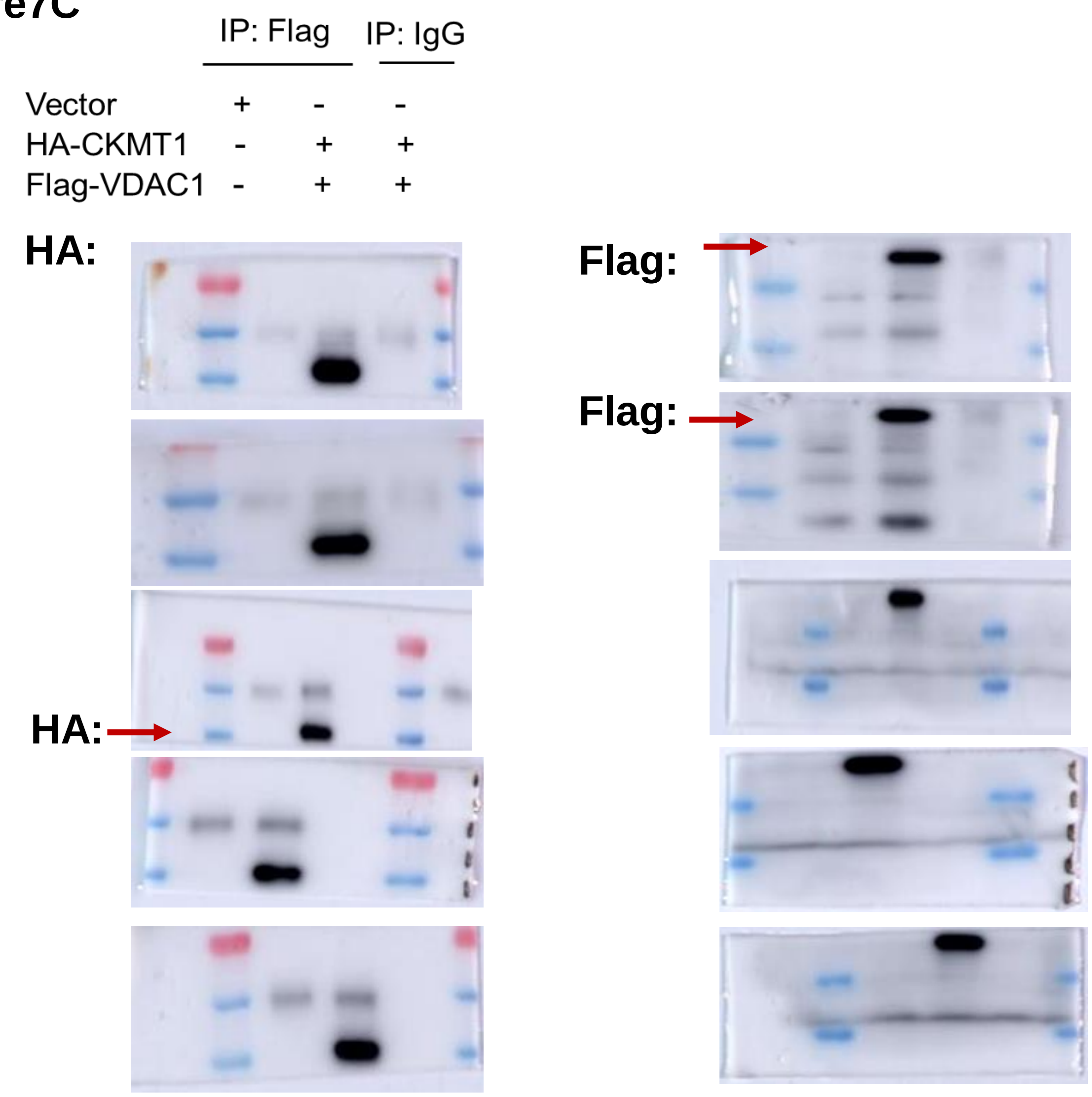

Figure 7B,C

Input

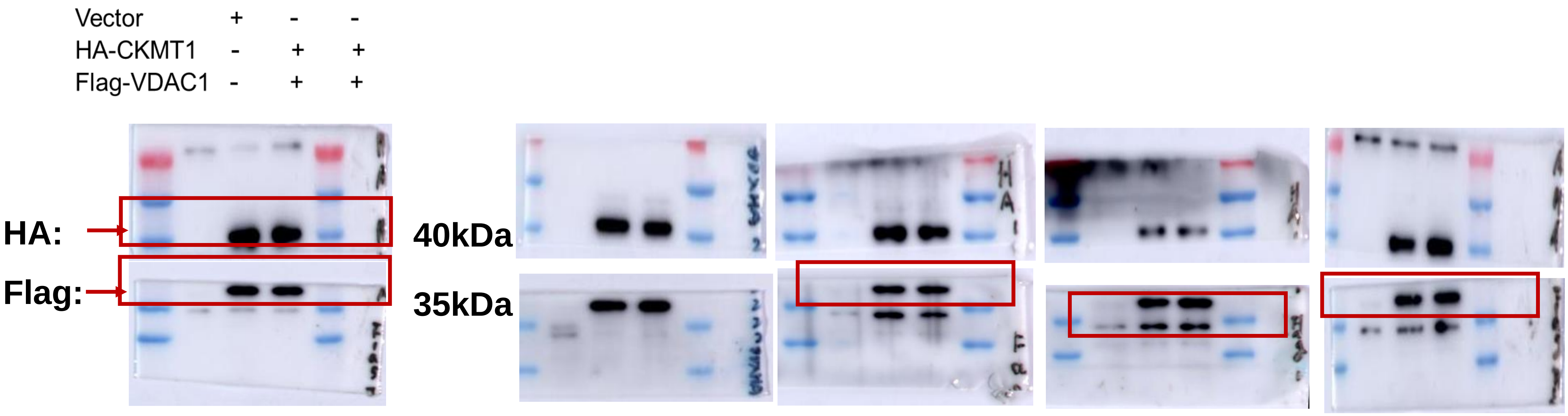

Figure 7 E

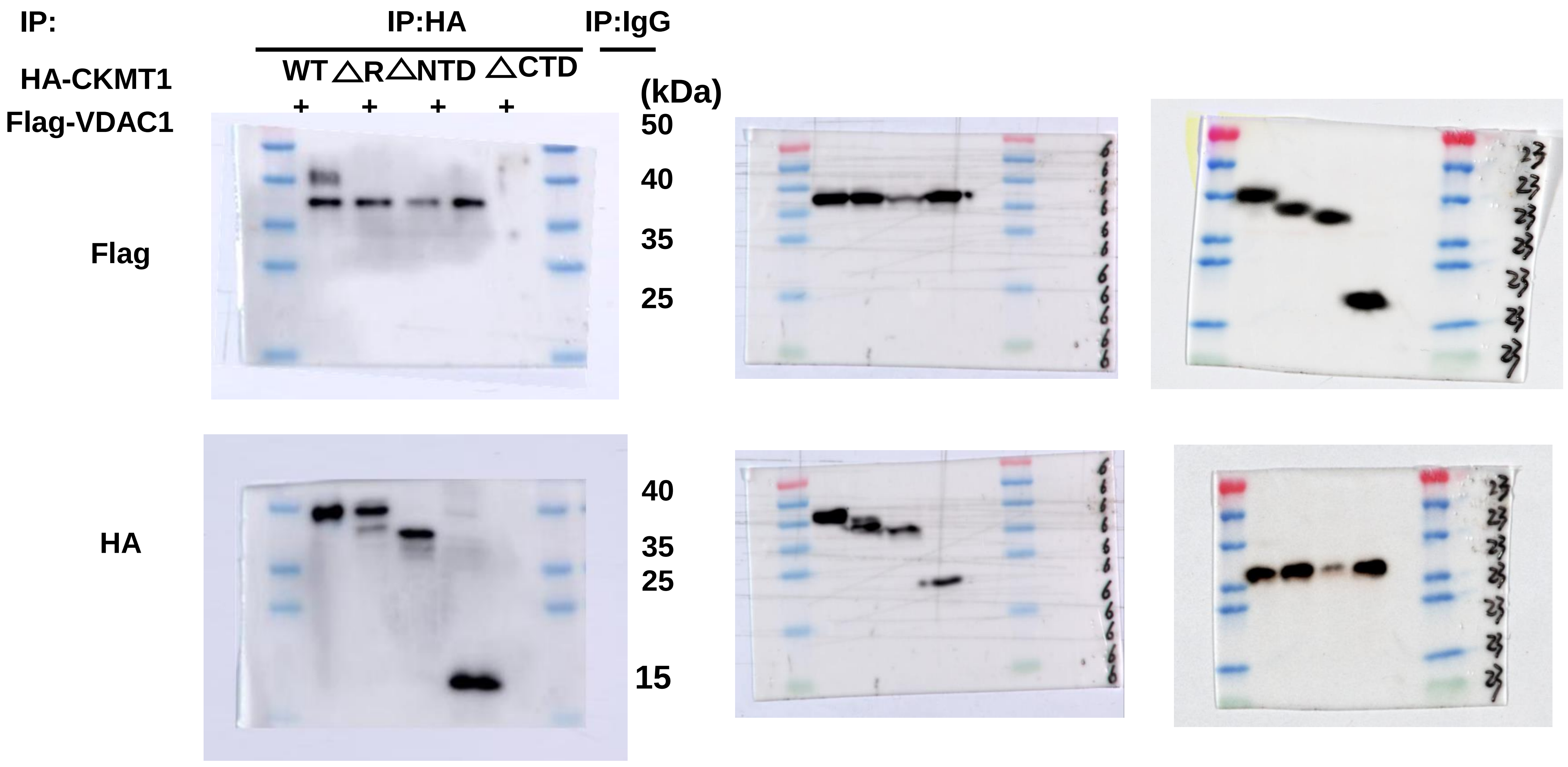
IP:
IP:HA
IP:IgG
HA-CKMT1
WT
△R
△NTD
△CTD
Flag-VDAC1
+ + + +
(kDa)
50
40
35
25
Flag
HA
40
35
25
15

Figure 7 E

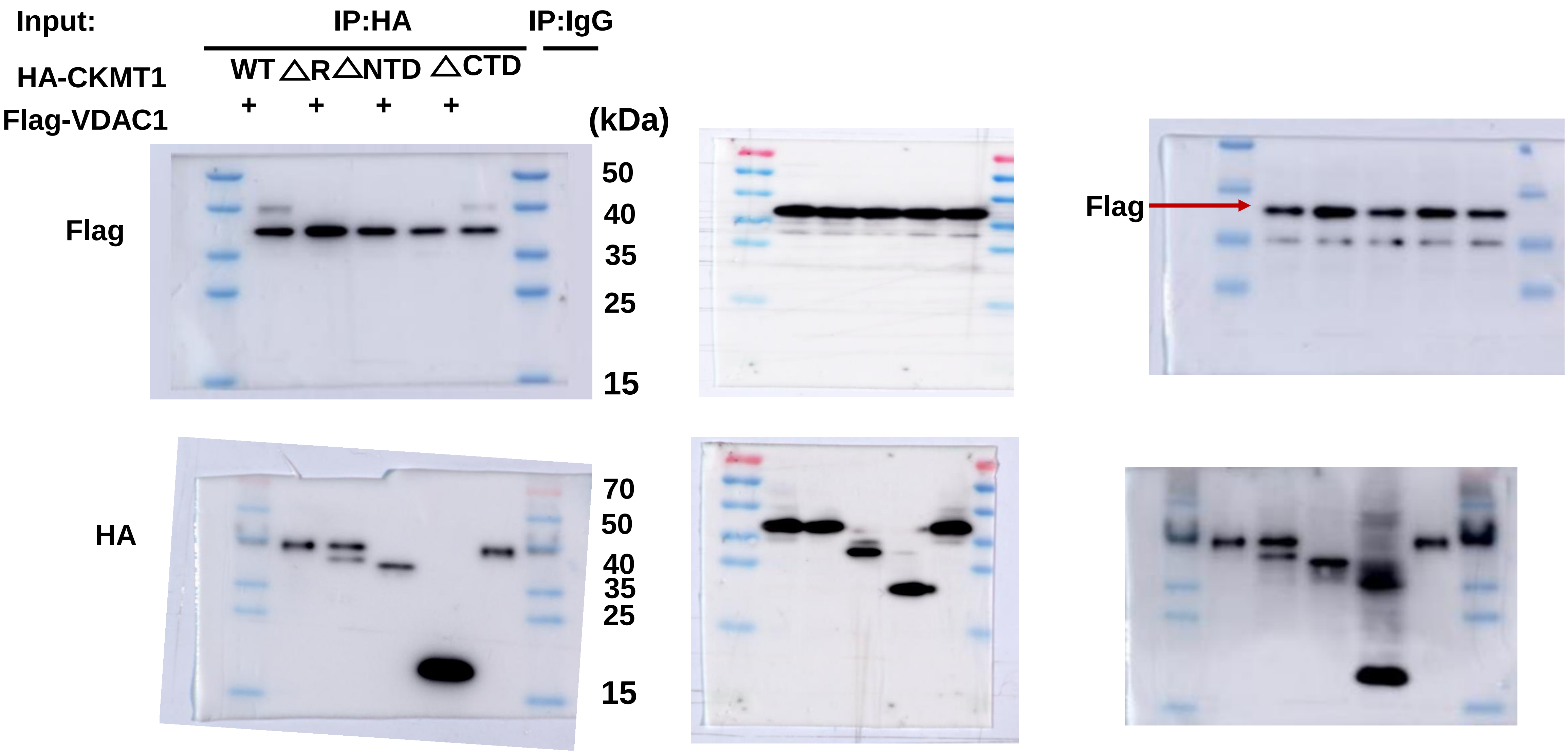

Input:
IP:HA
IP:IgG
HA-CKMT1
WT △R △NTD △CTD
Flag-VDAC1
+ + + +
(kDa)
Flag
50
40
35
25
15
Flag
HA
70
50
40
35
25
15

Figure 7 F

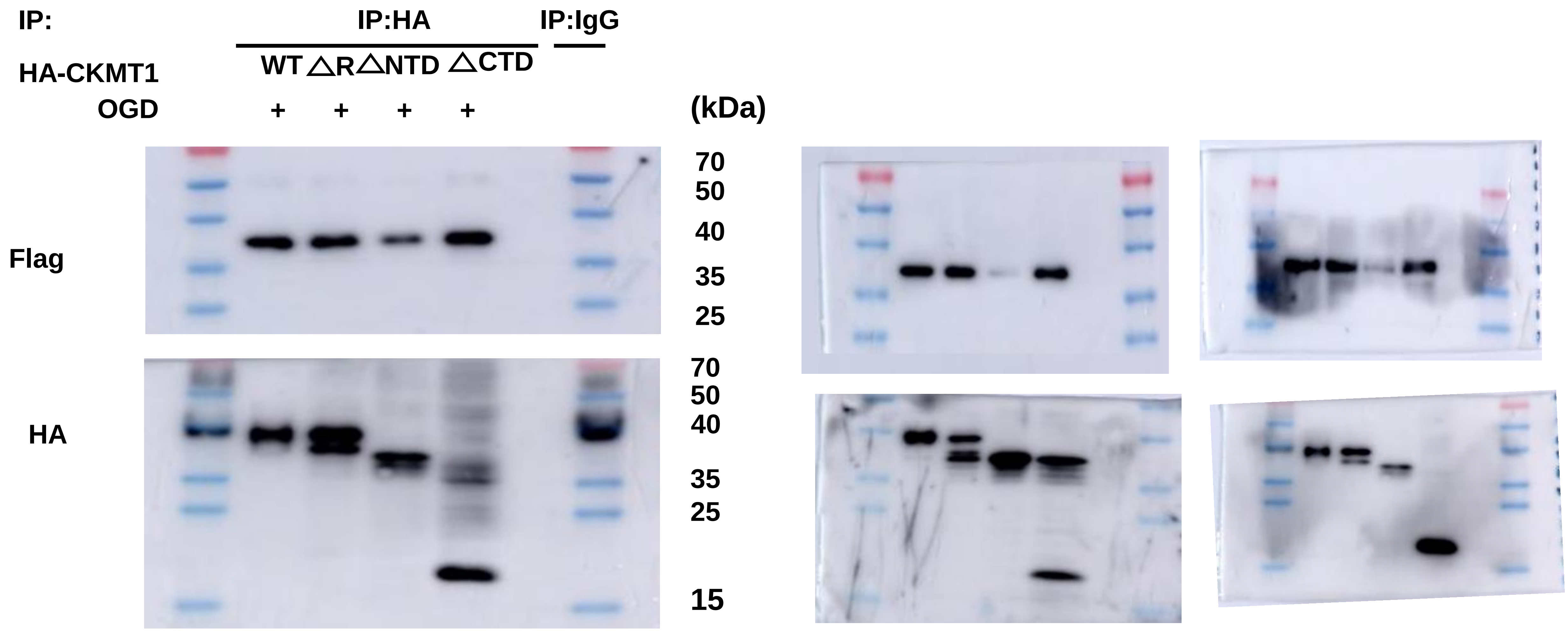
IP:
IP:HA
IP:IgG
HA-CKMT1
WT △R △NTD △CTD
OGD
+ + + +
(kDa)
70
50
40
35
25
Flag
70
50
40
35
25
15
HA

Figure 7 F

Input:

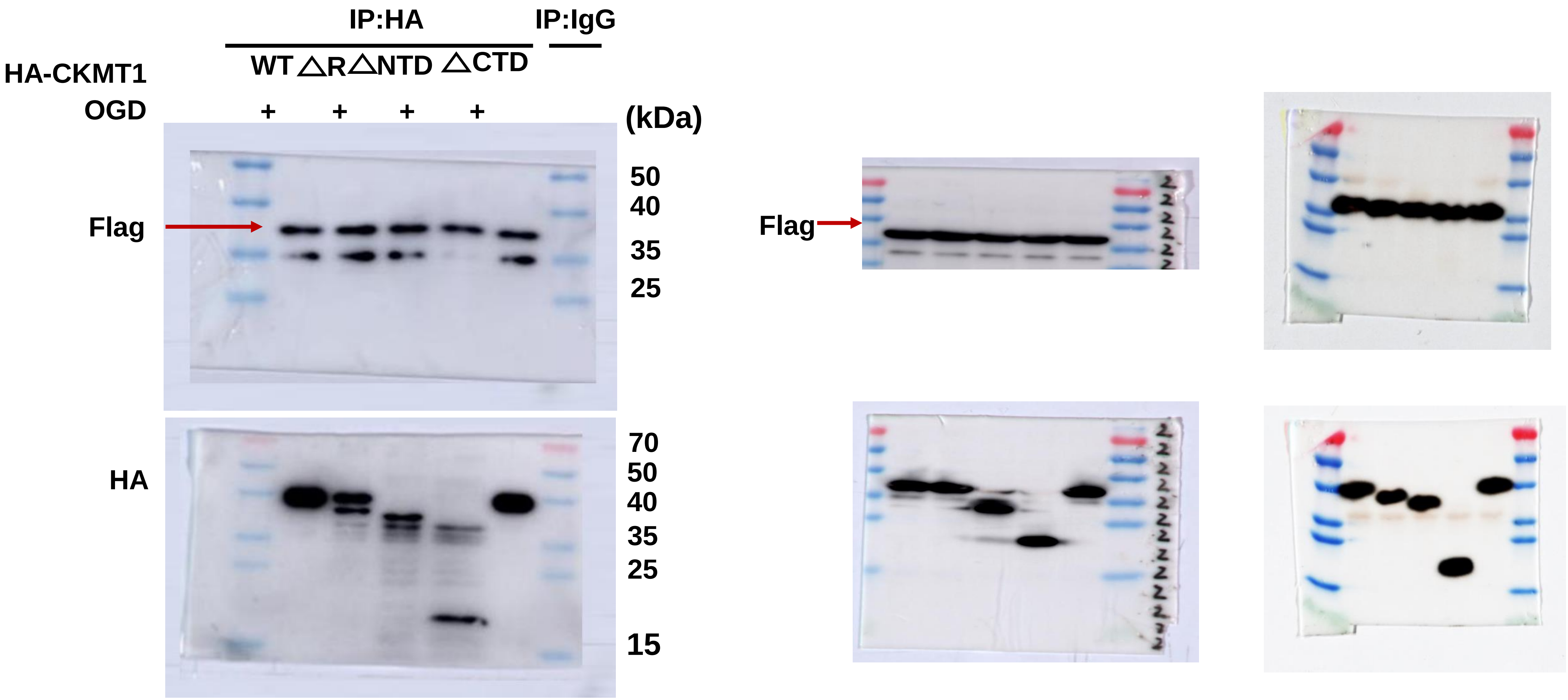

Figure7I

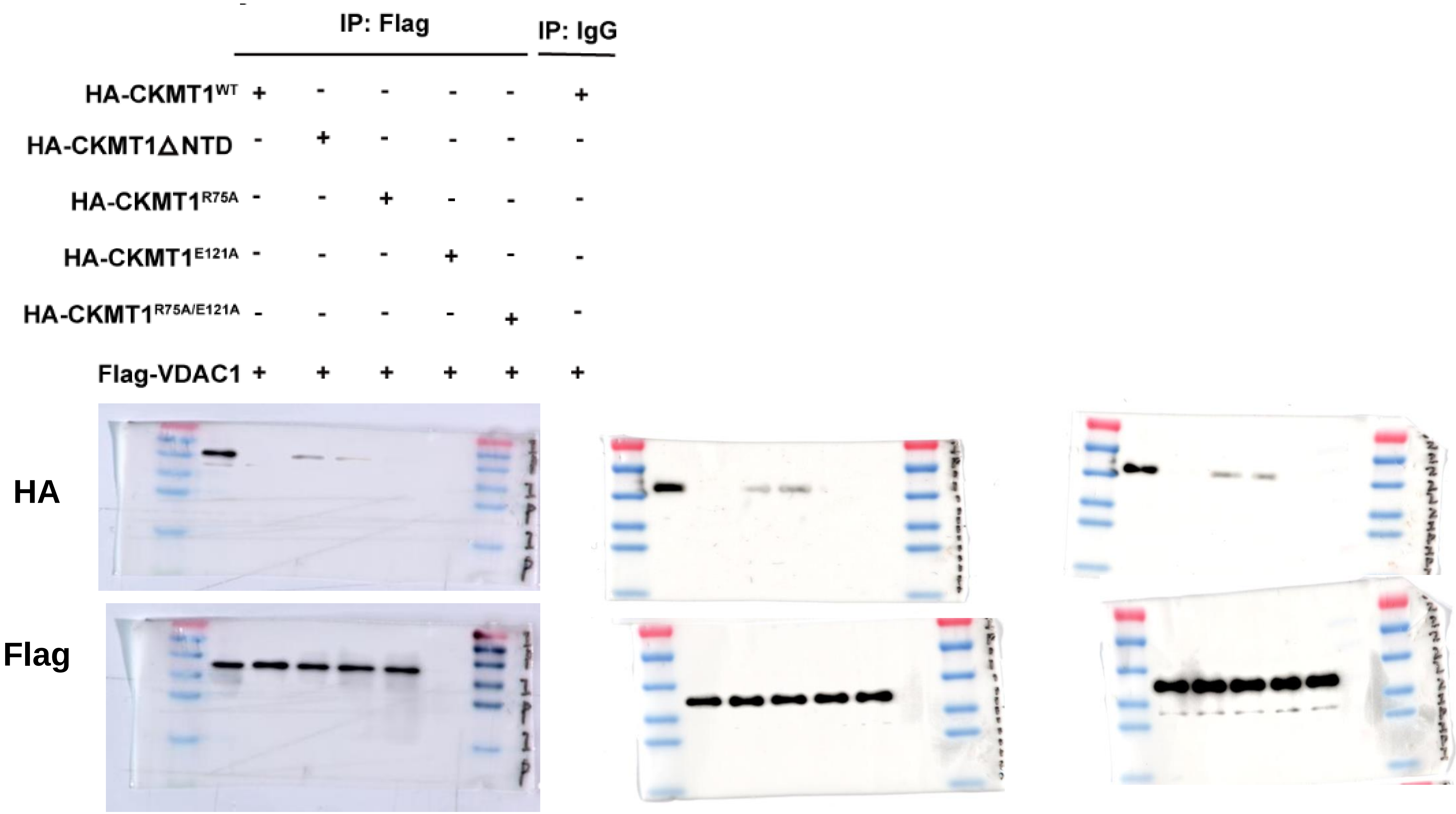
IP: Flag
IP: IgG
HA-CKMT1WT + - - - - +
HA-CKMT1ΔNTD - + - - - -
HA-CKMT1R75A - - + - - -
HA-CKMT1E121A - - - + - -
HA-CKMT1R75A/E121A - - - - + -
Flag-VDAC1 + + + + + +
HA
Flag

Figure 7J

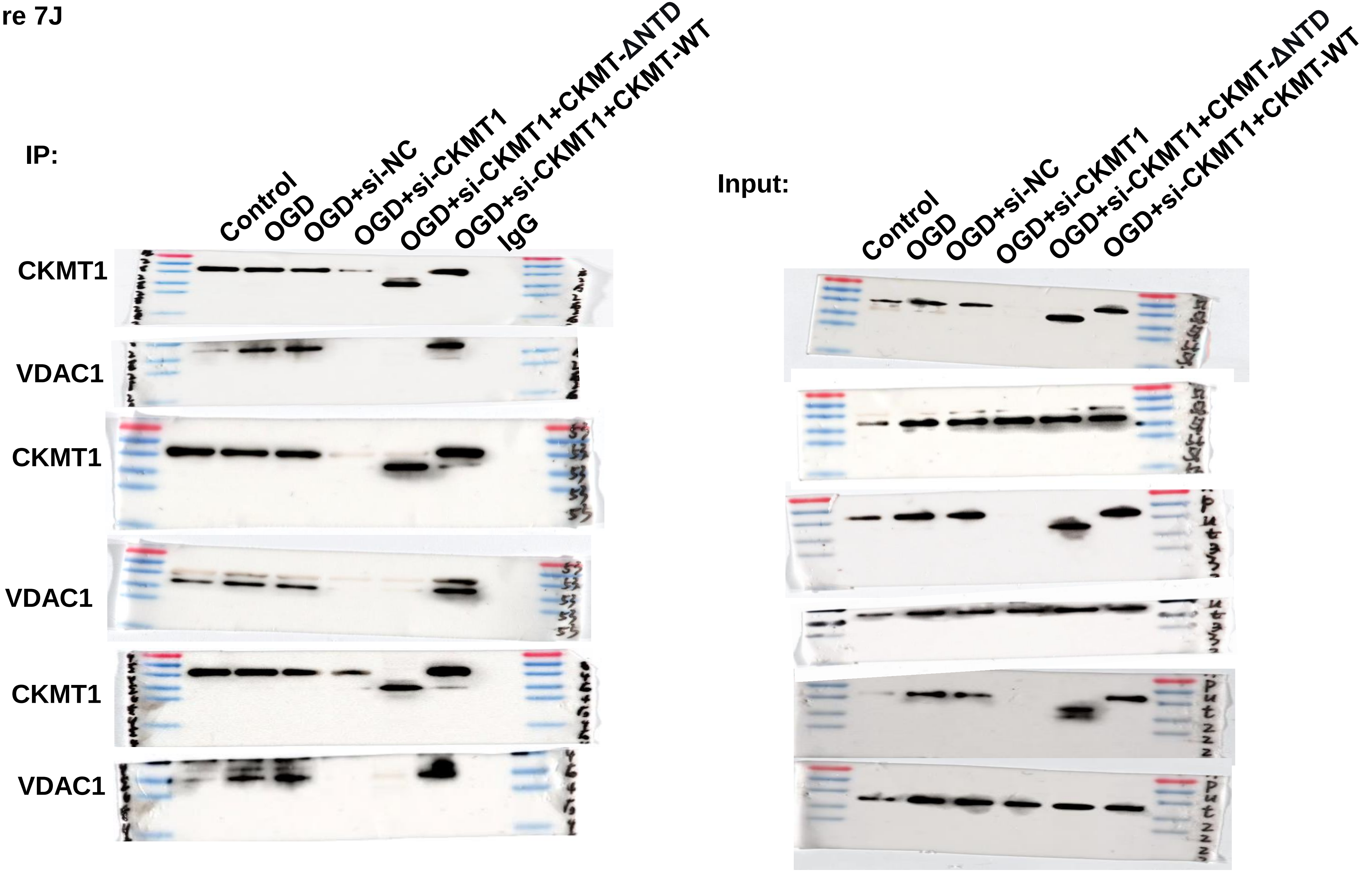
IP:
Control
OGD
OGD+si-NC
OGD+si-CKMT1
OGD+si-CKMT1+CKMT-ΔNTD
OGD+si-CKMT1+CKMT-WT
IgG
CKMT1
VDAC1
CKMT1
VDAC1
CKMT1
VDAC1
Input:
Control
OGD
OGD+si-NC
OGD+si-CKMT1
OGD+si-CKMT1+CKMT-ΔNTD
OGD+si-CKMT1+CKMT-WT

Figure 8C

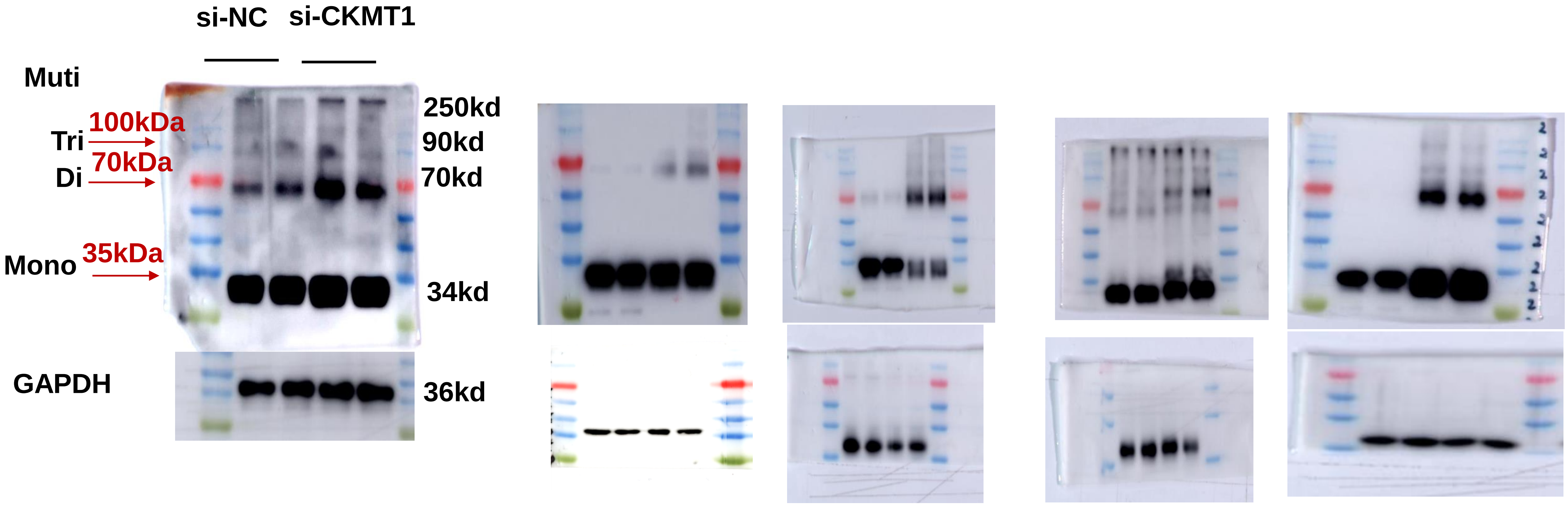

GAPDH was detected from the same sample without oligomerization treatment, as oligomerization impaired GAPDH signal.

Figure 8H

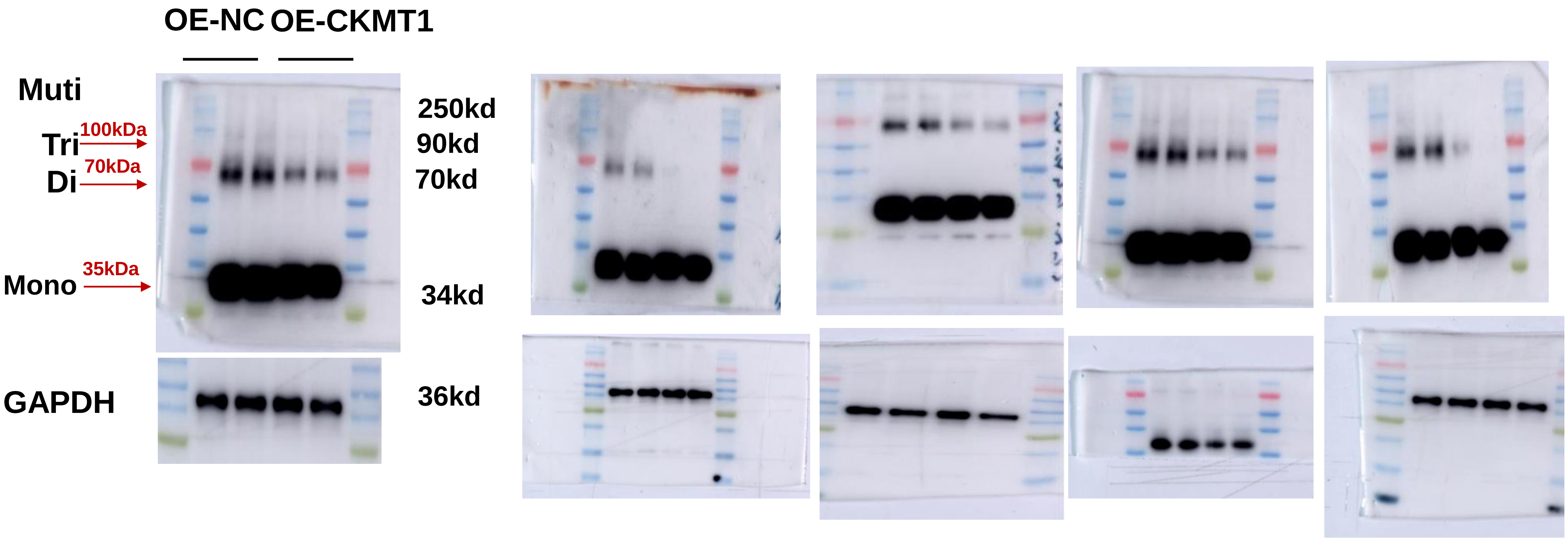

GAPDH was detected from the same sample without oligomerization treatment, as oligomerization impaired GAPDH signal.

Figure 8K

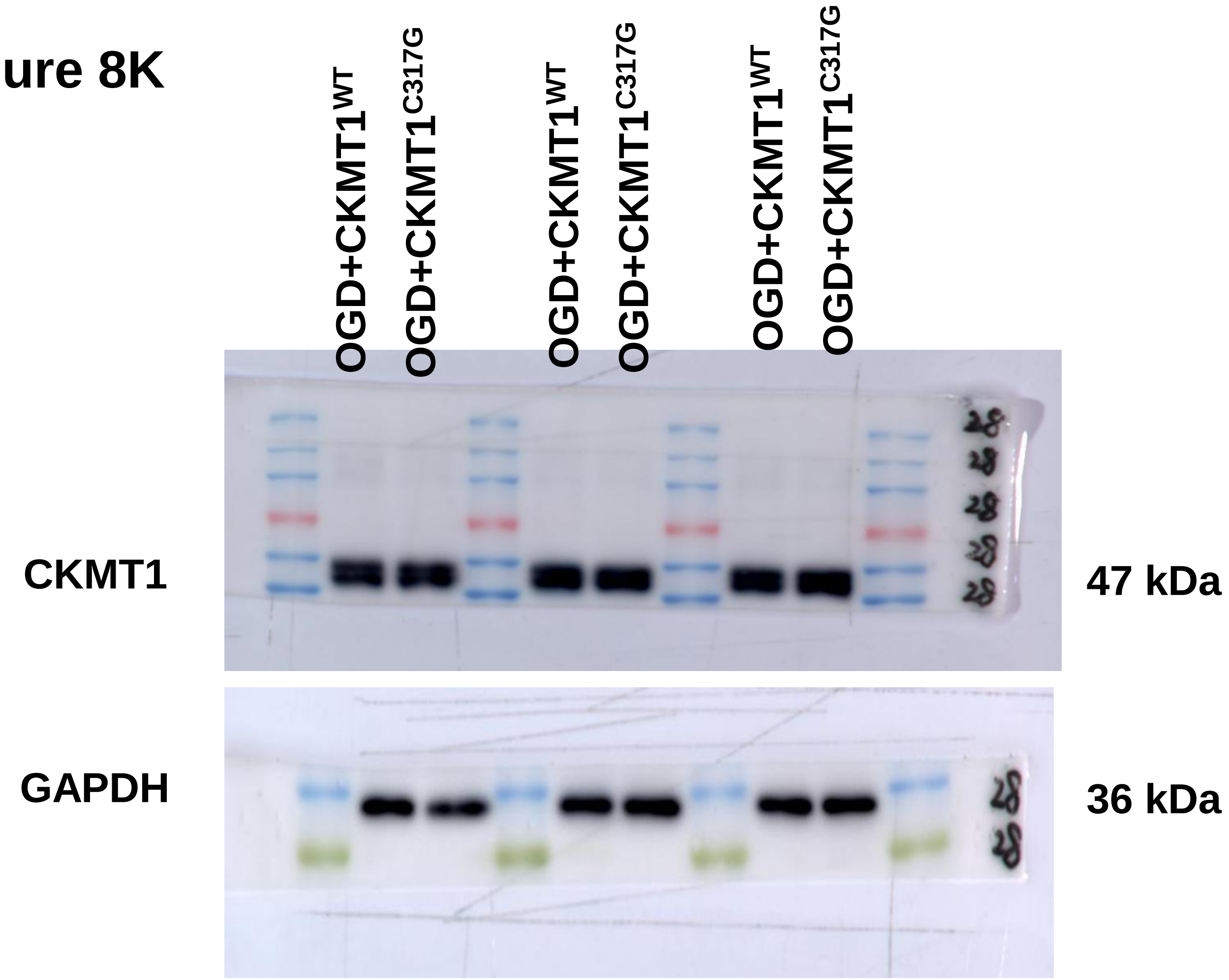
OGD+CKMT1WT
OGD+CKMT1C317G
OGD+CKMT1WT
OGD+CKMT1C317G
OGD+CKMT1WT
OGD+CKMT1C317G
CKMT1
47 kDa
GAPDH
36 kDa

Figure 8L

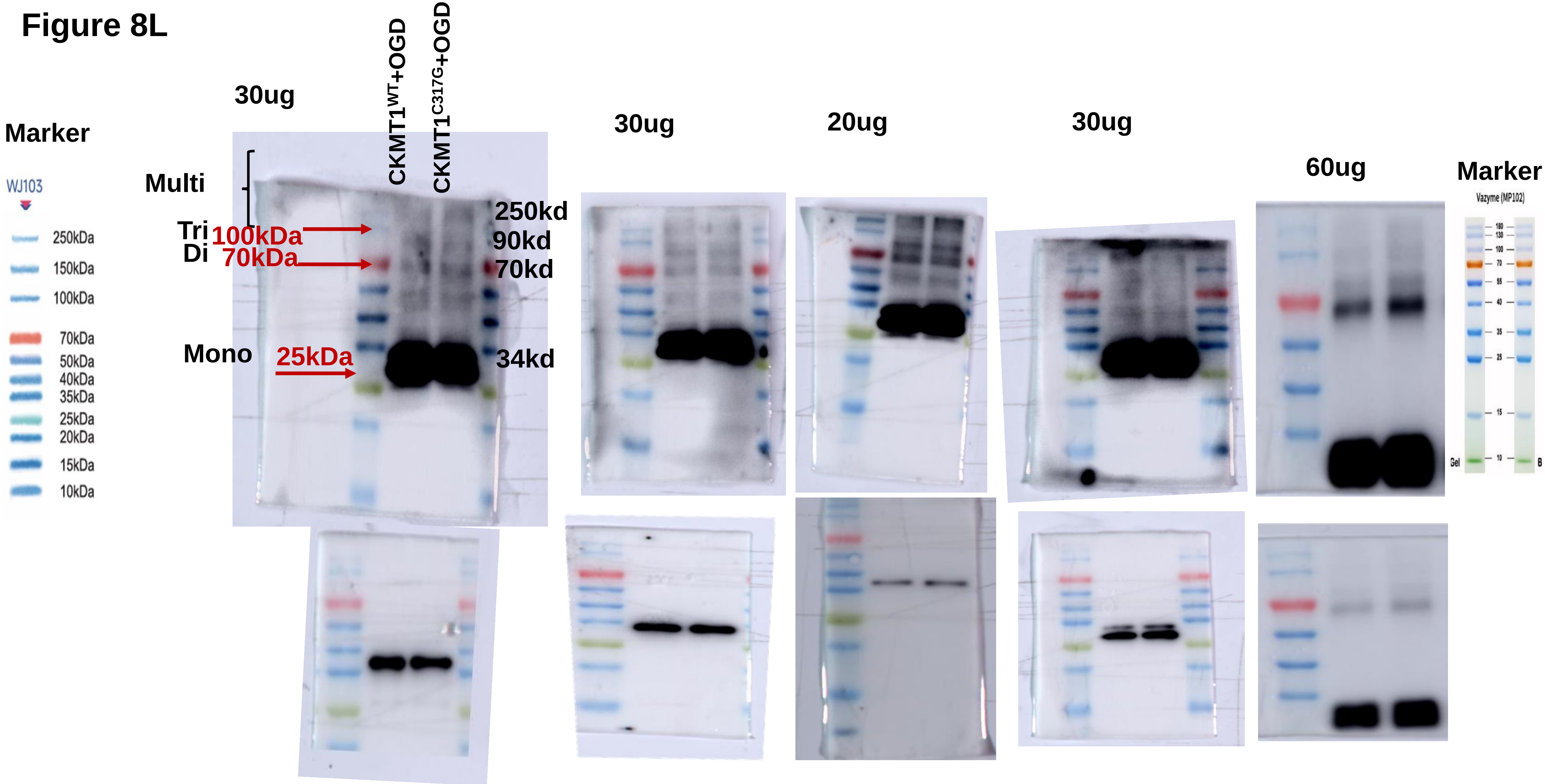

GAPDH was detected from the same sample without oligomerization treatment, as oligomerization impaired GAPDH signal.

Figure 8N

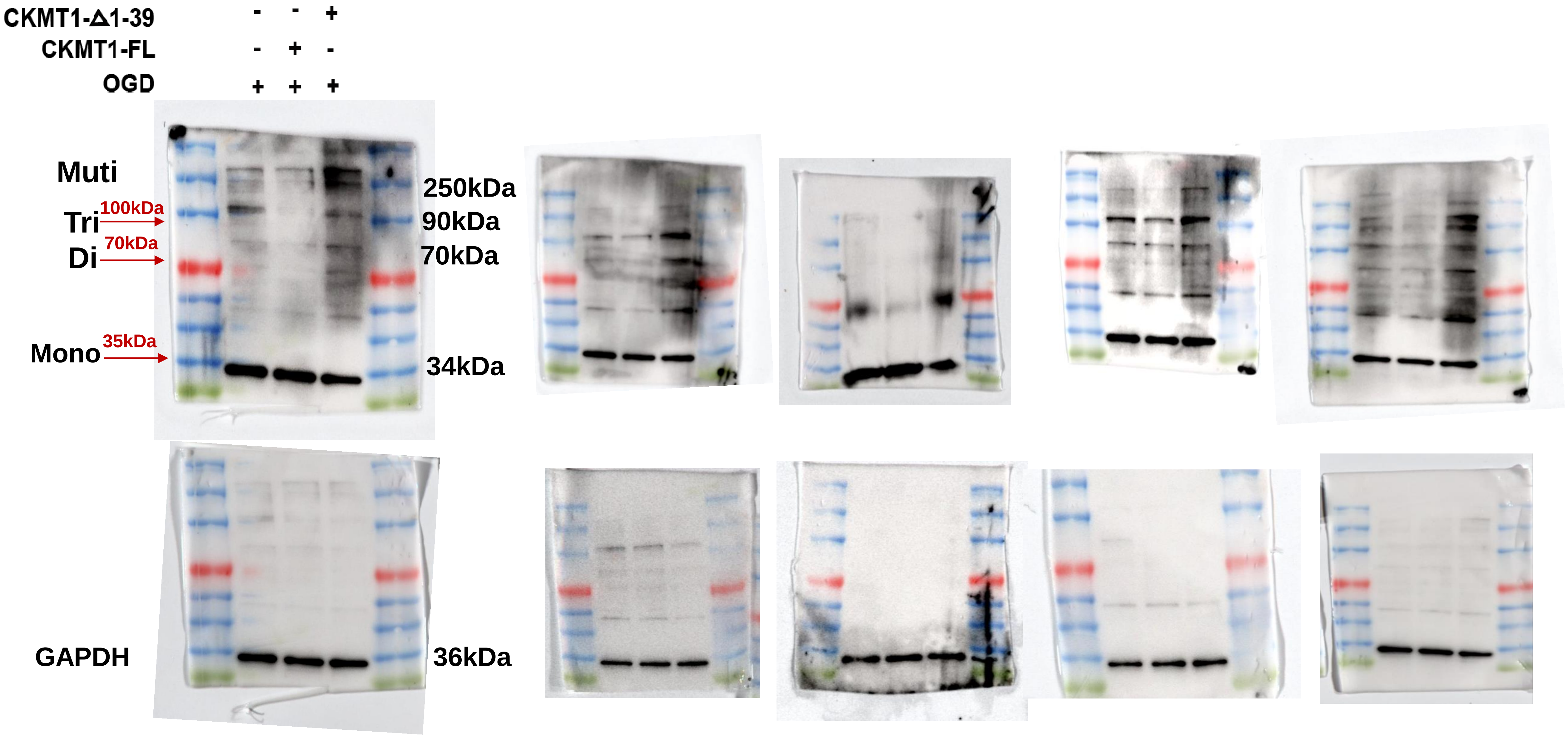
CKMT1-Δ1-39 - - +
CKMT1-FL - + -
OGD + + +
Muti
Tri
100kDa
Di
70kDa
Mono
35kDa
250kDa
90kDa
70kDa
34kDa
GAPDH
36kDa

Figure 9C

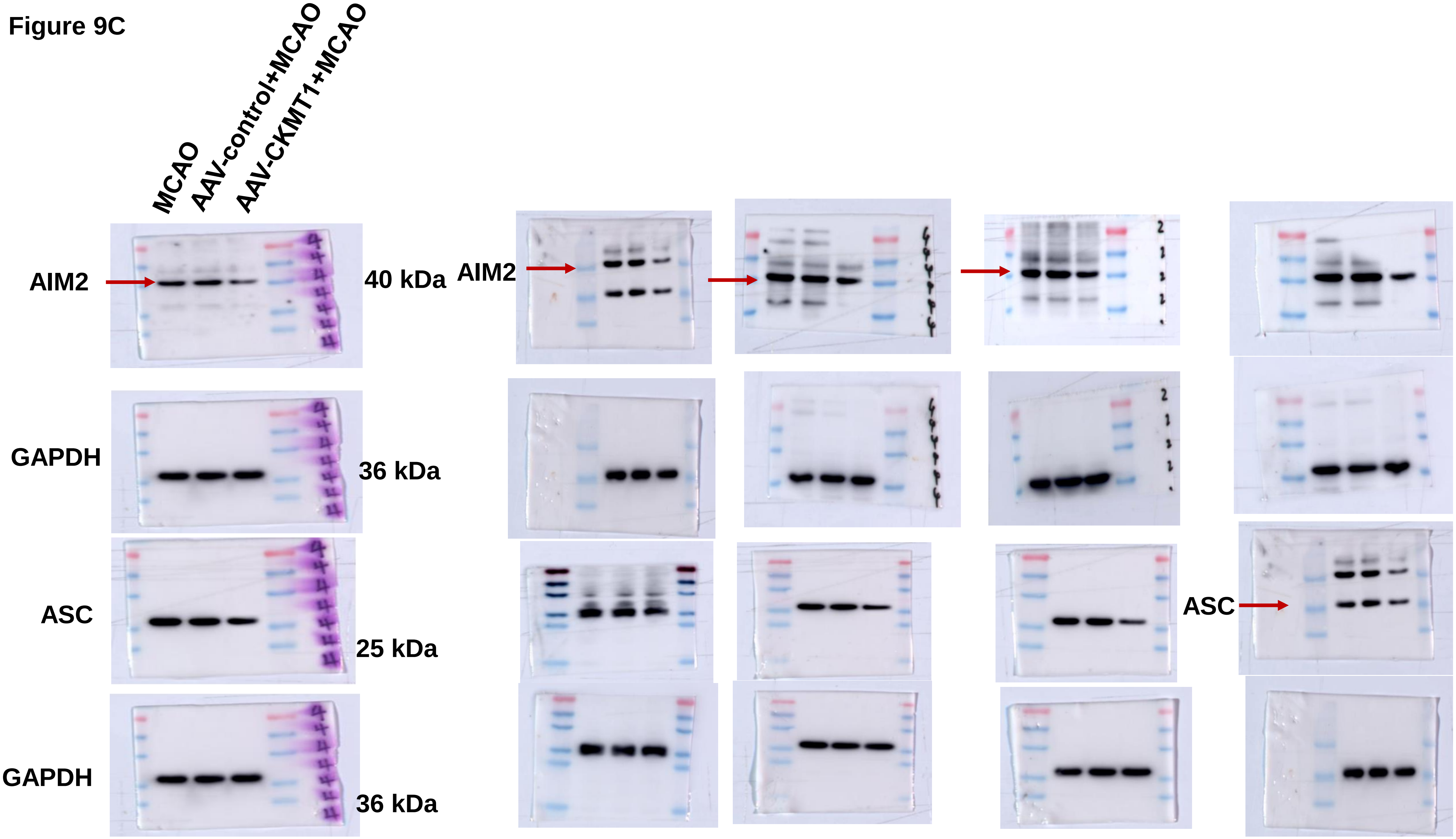

MCAO
AAV-control+MCAO
AAV-CKMT1+MCAO
AIM2
40 kDa
AIM2
GAPDH
36 kDa
ASC
25 kDa
ASC
GAPDH
36 kDa

Figure 9C

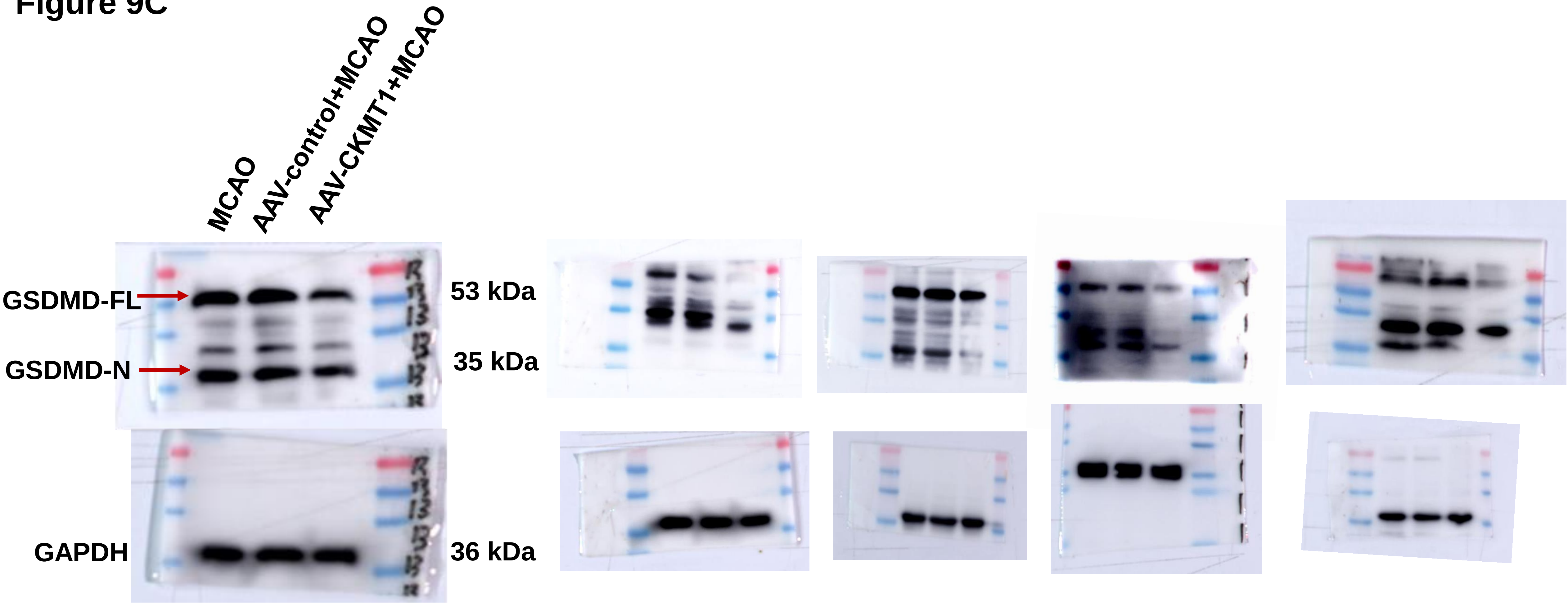

MCAO
AAV-control+MCAO
AAV-CKMT1+MCAO
GSDMD-FL
GSDMD-N
GAPDH
53 kDa
35 kDa
36 kDa

Figure 9C

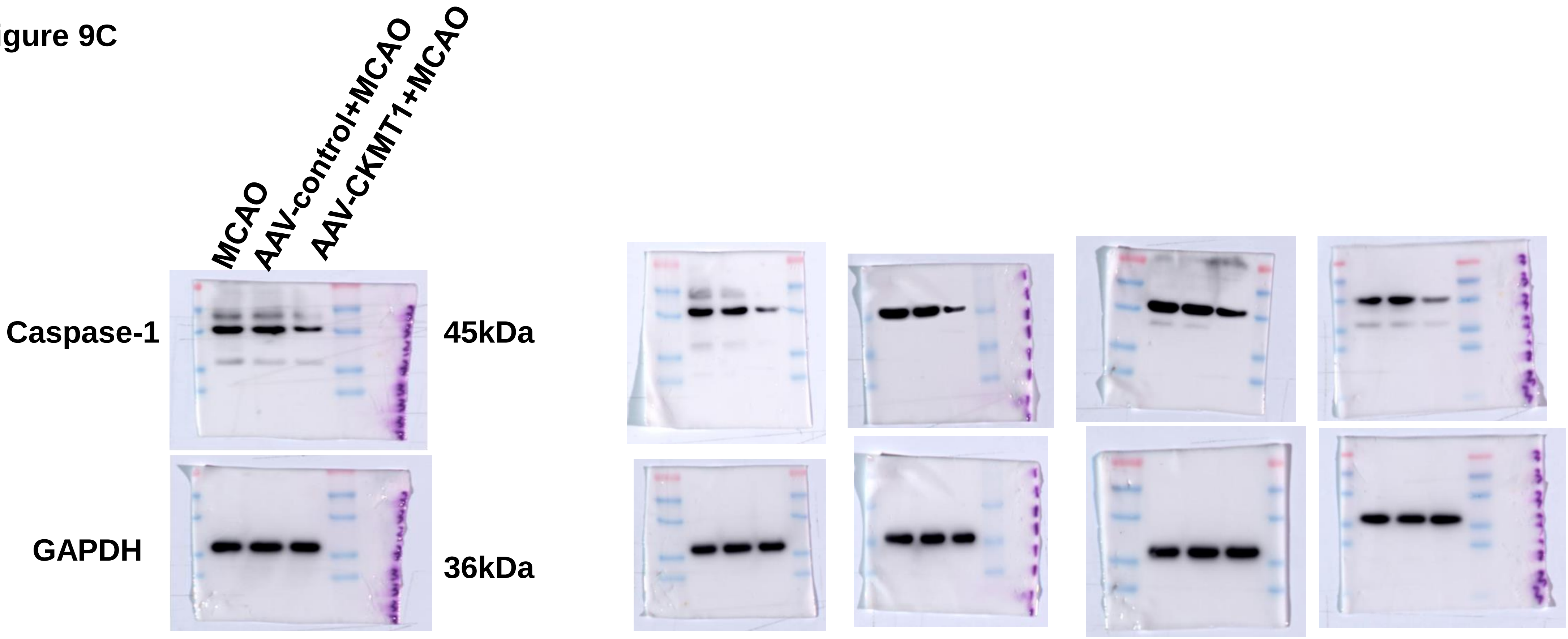
MCAO
AAV-control+MCAO
AAV-CKMT1+MCAO
Caspase-1
45kDa
GAPDH
36kDa

Figure 9C

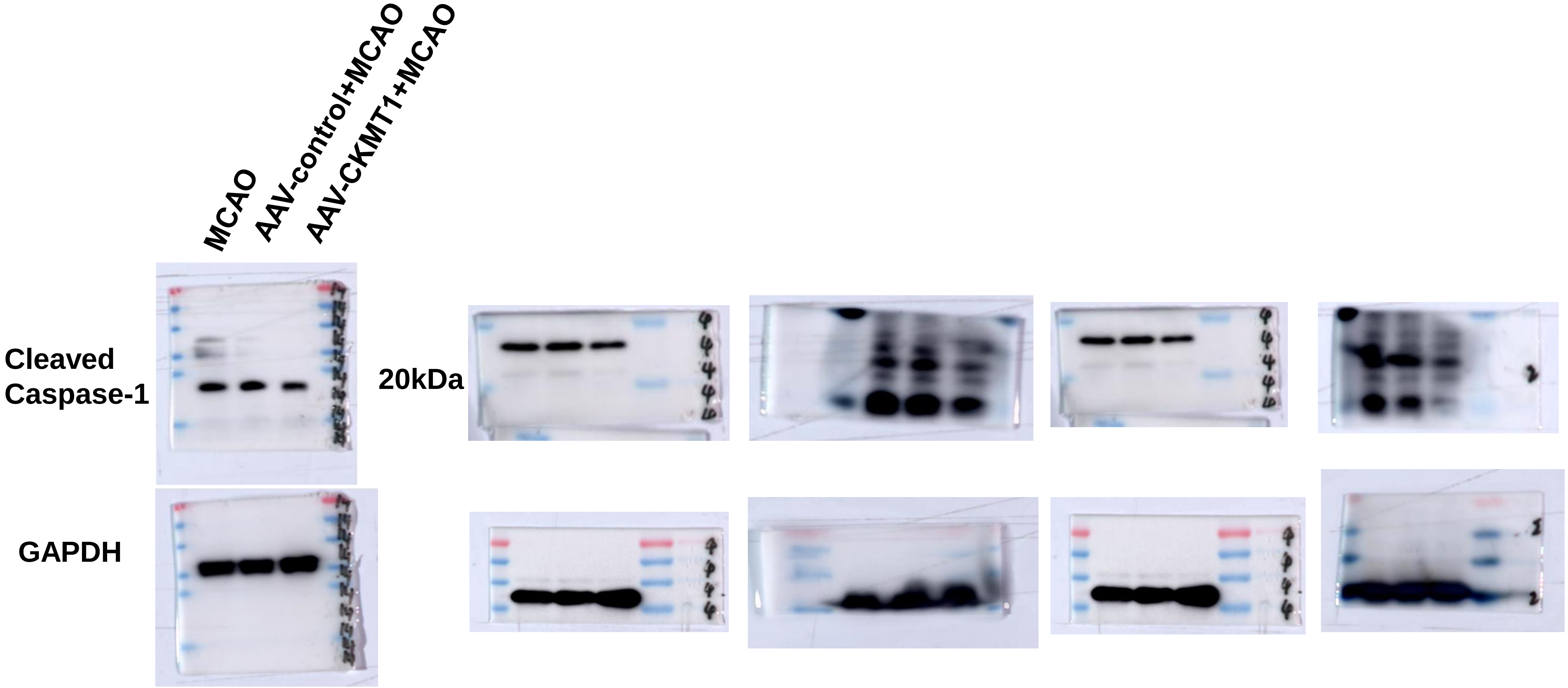
MCAO
AAV-control+MCAO
AAV-CKMT1+MCAO
Cleaved Caspase-1
20kDa
GAPDH

Supplementary Figure 1 A

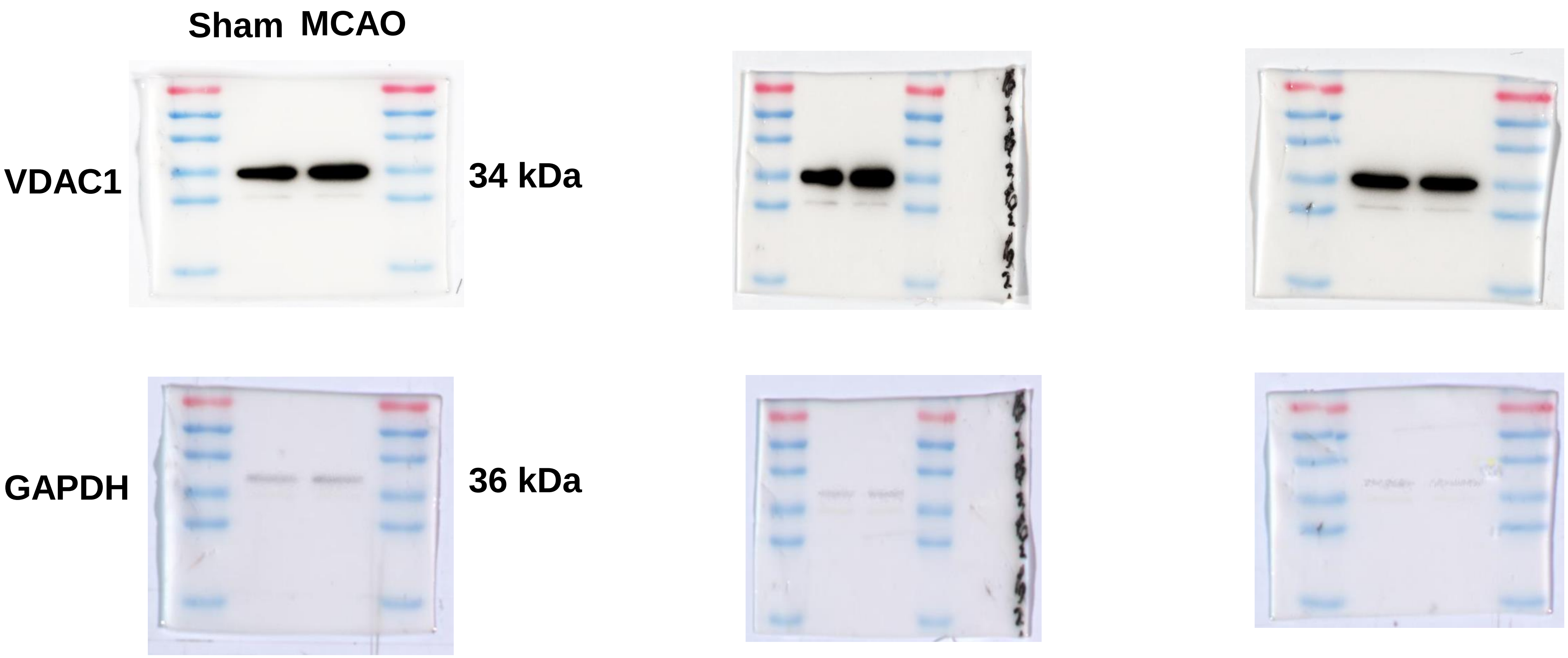

Supplementary Figure 3A

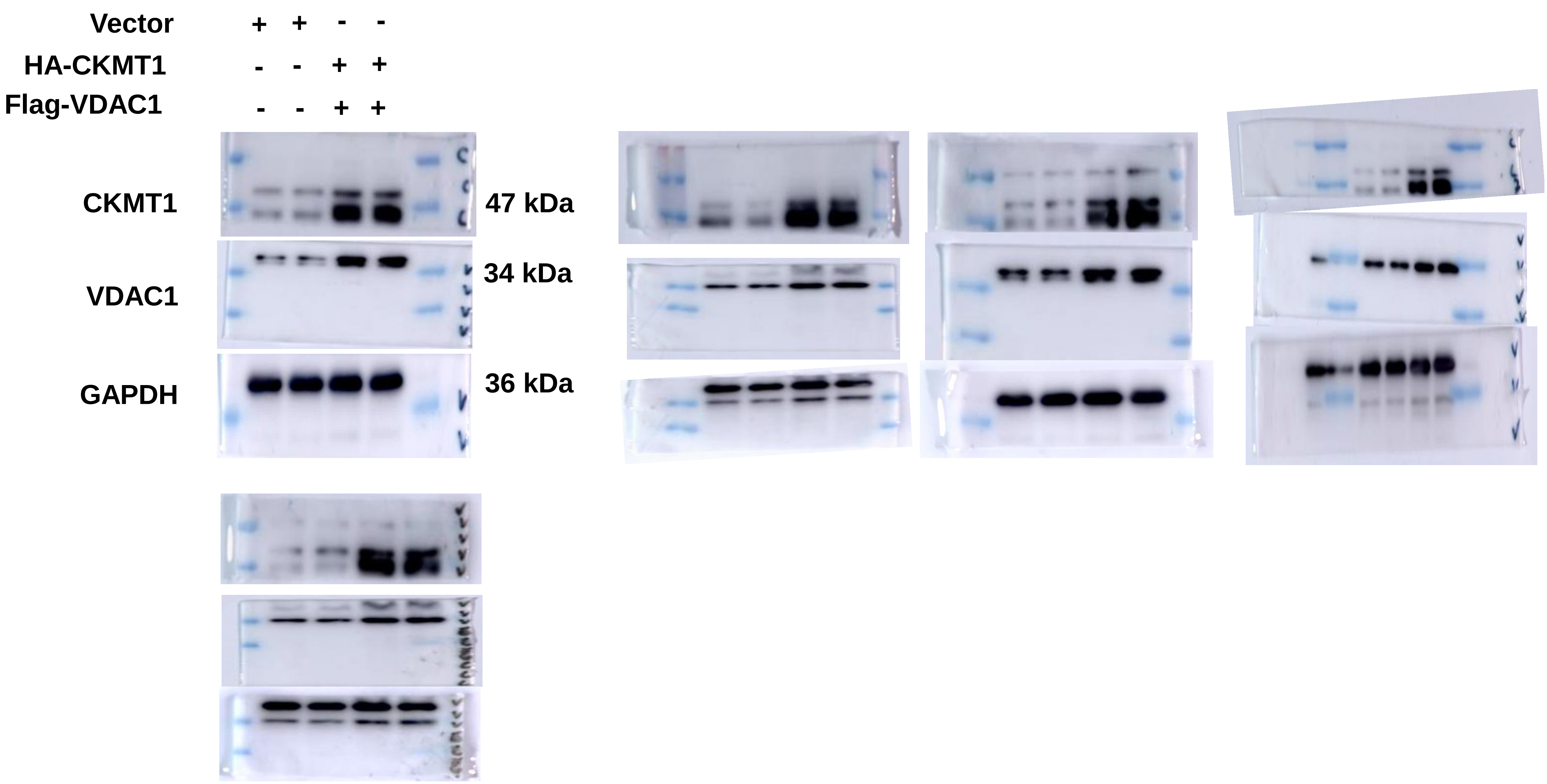

Supplementary Figure 4 B

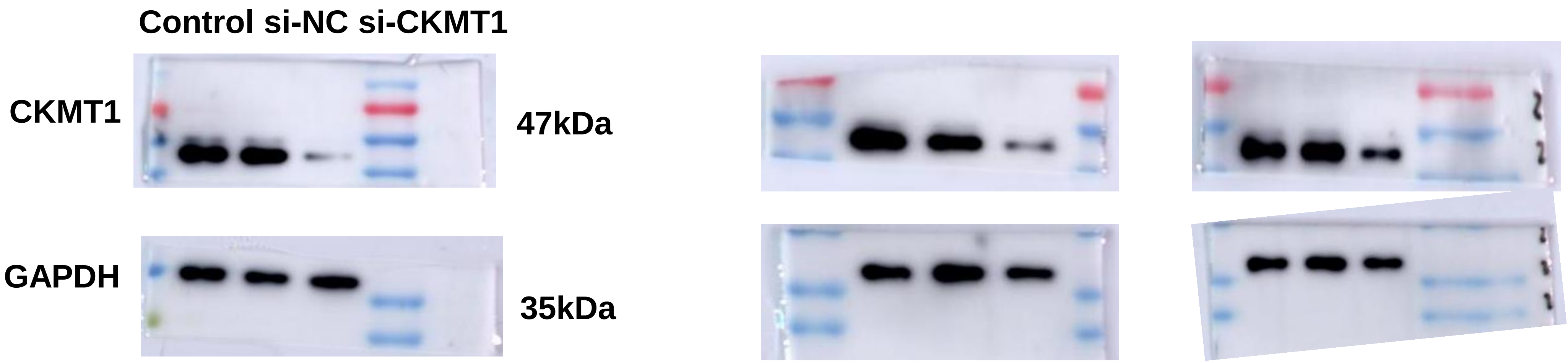

Supplementary Figure 4D

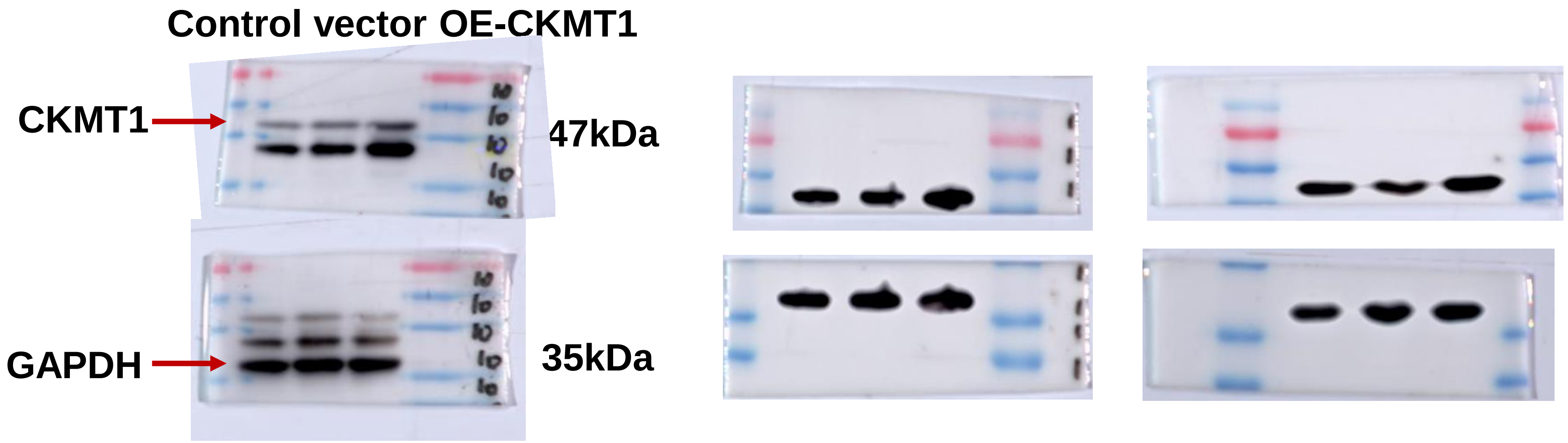

Supplementary Figure 5 A

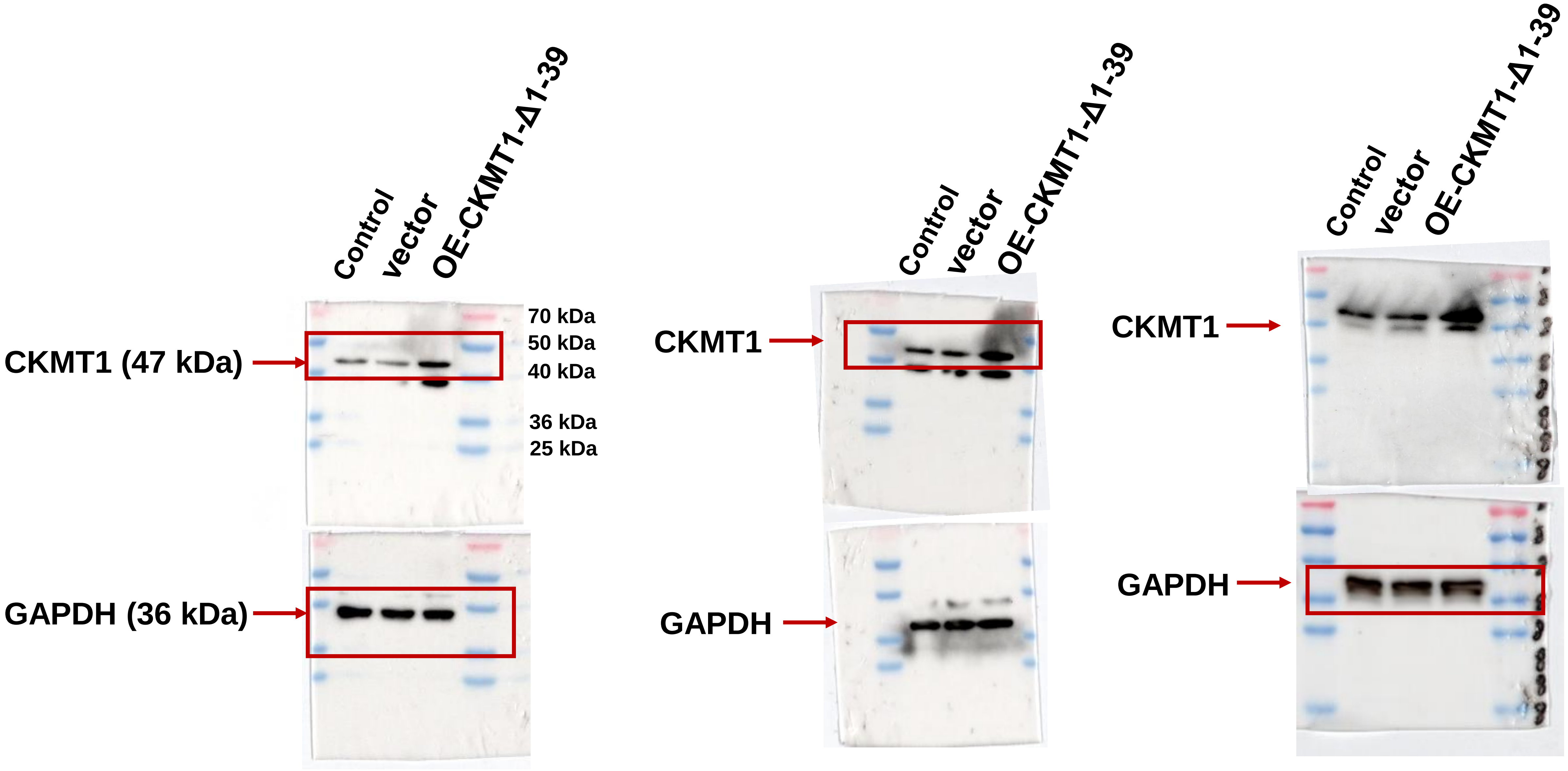

Supplementary Figure 7 B

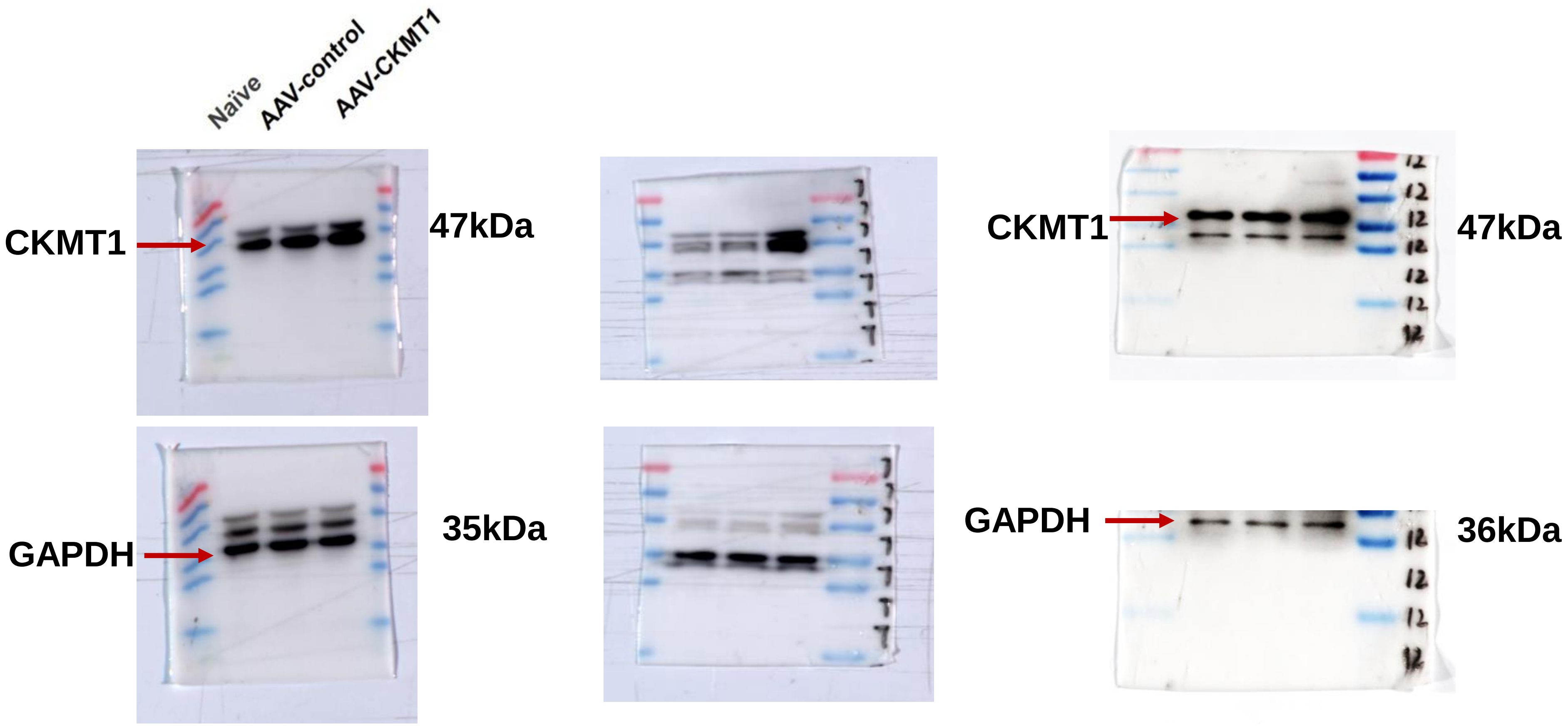

Supplement: Supplementary file 2 [file ADVS-9999-e78611-s005.pdf]
