# Supplementary material for: CKMT1 Counteracts VDAC1 Oligomerization‐Induced Neuronal Pyroptosis Through Steric Hindrance in Ischemic Stroke
Source: Adv Sci (Weinh). 2026 Nov 5:e78611. Online ahead of print. doi: 10.1002/advs.78611 (PMC13735431; doi:10.1002/advs.78611)
Supplement: Supplementary file 3 [file ADVS-9999-e78611-s007.docx]

**Supplementary Figure 1. Validation of mitochondrial purity.** (A) Western blot analysis of VDAC1 and GAPDH on the same membrane confirmed the purity of the isolated mitochondrial fraction, showing strong VDAC1 enrichment and minimal GAPDH signal. n = 3 independent experiments.

**Supplementary Figure 2. Determination of effective concentrations of VBIT-4 and EtBr in vitro.** (A) Cell viability of PC12 cells subjected to OGD with pretreatment of different concentrations of VBIT-4 (5, 10, and 20 μM), assessed by CCK-8 assay. (B) Cytosolic mtDNA levels in PC12 cells after OGD treated with the indicated concentrations of EtBr, quantified by qPCR. (C) Cell viability of PC12 cells after OGD treated with different concentrations of ethidium bromide (EtBr: 250, 500, and 1000 ng/mL), assessed by CCK-8 assay. Data are presented as mean ± SD. Statistical significance was determined using one-way ANOVA followed by Tukey's post hoc test. The number of biologically independent replicates (n) for each panel is as follows: n = 5 (A–C). Exact *P* values are shown in the figure panels.

**Supplementary Figure 3. Verification of VDAC1 and CKMT1 co-overexpression in vitro.** (A) Western blot analysis of VDAC1 and CKMT1 protein levels in HEK293T cells co-transfected with Flag-VDAC1 and HA-CKMT1 plasmids or empty vector control for 48 h. Protein levels were quantified and normalized to GAPDH. Data are presented as mean ± SD. Statistical significance was determined using a two-tailed unpaired Student's t-test. The number of biologically independent replicates (n) for (A) is 5. Exact *P* values are shown in the figure panels.

**Supplementary Figure 4. Efficiency of CKMT1 knockdown or overexpression in vitro.** (A) CKMT1 mRNA measured by qRT-PCR in PC12 cells at 48 h after transfection with CKMT1-targeting siRNA (si-CKMT1) or a non-targeting control (si-NC). (B) Western blot analysis of CKMT1 protein level in PC12 cells under the same conditions as in (A). Protein expression was normalized to GAPDH. (C) CKMT1 mRNA measured by qRT-PCR in PC12 cells at 48 h after transfection with a CKMT1 overexpression plasmid (CKMT1-OE) or an empty vector control. (D) Western blot analysis of CKMT1 protein level in PC12 cells under the same conditions as in (C). Protein level was normalized to GAPDH. Data are presented as mean ± SD. Statistical significance was determined by one-way ANOVA followed by Tukey's post-hoc test (A, B, D) or Kruskal–Wallis test followed by Dunn's multiple-comparisons test (C). Sample sizes (n) represent biologically independent cultures: n = 5 for A and C, n = 3 for B and D. Exact *P* values are shown in the figure panels.

**Supplementary Figure 5. Validation of CKMT1 Δ1-39 mutant expression.** (A) Western blot analysis of CKMT1 and GAPDH confirmed expression of full-length CKMT1 in the control and control+vector groups, and expression of the truncated protein in the CKMT1 Δ1-39 group. Protein levels were quantified and normalized to GAPDH. Data are presented as mean ± SD. Statistical significance was determined using one-way ANOVA followed by Tukey's post hoc test. The number of biologically independent replicates (n) for (A) is 5. Exact *P* values are shown in the figure panels.

**Supplementary Figure 6. Identification of a VDAC1-interacting signal peptide within CKMT1.** (A) LC-MS analysis identifying a peptide (sequence: QLELFGK, residues 18-24) derived from the CKMT1 signal peptide (amino acids 1-39) in VDAC1 immunoprecipitates from PC12 cells subjected to OGD. Representative spectra mapping this peptide are shown for control and OGD samples. Three independent cell cultures were pooled as one sample for each condition.

**Supplementary Figure 7. Validation of AAV delivery and CKMT1 overexpression in vivo.** (A) Western blot analysis of CKMT1 protein levels in cortical tissue from rats pre-injected with neuron-specific AAV-CKMT1 or control AAV. Protein levels were quantified and normalized to GAPDH. Data are presented as mean ± SD. Statistical significance was determined using one-way ANOVA followed by Tukey's post hoc test. The number of biologically independent replicates (n) for each panel is as follows: n = 3. Exact *P* values are shown in the figure panels.
